# Superoxide anion radicals induce IGF-1 resistance through concomitant activation of PTP1B and PTEN

**DOI:** 10.15252/emmm.201404082

**Published:** 2014-12-17

**Authors:** Karmveer Singh, Pallab Maity, Linda Krug, Patrick Meyer, Nicolai Treiber, Tanja Lucas, Abhijit Basu, Stefan Kochanek, Meinhard Wlaschek, Hartmut Geiger, Karin Scharffetter-Kochanek

**Affiliations:** 1 Department of Dermatology and Allergic Diseases, University of UlmUlm, Germany; 2 Aging Research Center (ARC)Ulm, Germany; 3 Department of Gene Therapy, University of UlmUlm, Germany; 4 Institute of Molecular Medicine and Stem Cell Aging, University of UlmUlm, Germany; 5 Division of Experimental Hematology and Cancer Biology, Cincinnati Children's Hospital Medical Center and University of CincinnatiCincinnati, OH, USA

**Keywords:** ageing, IGF-1, phosphatase, reactive oxygen species, superoxide anions

## Abstract

The evolutionarily conserved IGF-1 signalling pathway is associated with longevity, metabolism, tissue homeostasis, and cancer progression. Its regulation relies on the delicate balance between activating kinases and suppressing phosphatases and is still not very well understood. We report here that IGF-1 signalling *in vitro* and in a murine ageing model *in vivo* is suppressed in response to accumulation of superoxide anions (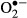) in mitochondria, either by chemical inhibition of complex I or by genetic silencing of 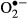-dismutating mitochondrial Sod2. The 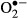-dependent suppression of IGF-1 signalling resulted in decreased proliferation of murine dermal fibroblasts, affected translation initiation factors and suppressed the expression of α1(I), α1(III), and α2(I) collagen, the hallmarks of skin ageing. Enhanced 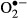 led to activation of the phosphatases PTP1B and PTEN, which via dephosphorylation of the IGF-1 receptor and phosphatidylinositol 3,4,5-triphosphate dampened IGF-1 signalling. Genetic and pharmacologic inhibition of PTP1B and PTEN abrogated 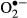-induced IGF-1 resistance and rescued the ageing skin phenotype. We thus identify previously unreported signature events with 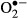, PTP1B, and PTEN as promising targets for drug development to prevent IGF-1 resistance-related pathologies.

## Introduction

Insulin-like growth factors (IGFs) play essential roles in the regulation of cell growth, proliferation, stem cell maintenance and synthesis of extracellular matrix proteins (Baker *et al*, 1993; Le Roith, 1997; Papaconstantinou, 2009; Piecewicz *et al*, 2012) and—if dysregulated—result in connective tissue and organ atrophy with enhanced ageing or cancer progression (Baker *et al*, 1993; Pollak *et al*, 2004; Govoni *et al*, 2007b; Laviola *et al*, 2008; Anisimov & Bartke, 2013). During ageing, a gradual decline in circulating insulin-like growth factor-1 (IGF-1) levels beginning in the third and fourth decade of life occurs in humans and in other mammals (Lamberts *et al*, 1997; Le Roith, 1997; Tatar *et al*, 2003; Parekh *et al*, 2010). Reduced IGF-1 levels and/or impaired IGF-1 signal transduction may at least in part be responsible for enhanced muscle atrophy (sarcopenia), bone resorption (osteoporosis), and skin atrophy consistently observed in elderly individuals (Gallagher & LeRoith, 2011). Given the significant clinical implications and the current demographic development, advanced knowledge on IGF-1 signalling and its control at different steps of the signalling cascade is particularly important. Regulation of IGF-1 signalling occurs rather in integrated signalling networks, which are governed at the level of phosphorylation and dephosphorylation, catalysed by protein kinases and phosphatases, respectively (Pollak *et al*, 2004; Taguchi & White, 2008). Similar to other growth factors, IGF-1 signalling is initiated by the autophosphorylation of the IGF-1 receptor β subunit (IGF-1Rβ), which is followed by a series of kinase-dependent phosphorylations of downstream effectors such as phosphoinositide-3-kinase (PI3K), AKT (protein kinase B), and p70S6 ribosomal protein kinase (p70S6K)—a prerequisite for synthesis of extracellular matrix—and other proteins. This in conjunction with the IGF-1-initiated Ras-dependent upregulation of cyclin D1 ultimately promotes cell cycle progression and growth essential for overall tissue homeostasis (Pollak *et al*, 2004; Samani *et al*, 2007). In addition to the PI3K–AKT axis, IGF-1 signalling was reported to activate the mitogen-activated protein kinase (MAPK) pathway, thus exerting its prosurvival effect in many though not all cell lines (Parrizas *et al*, 1997; Peruzzi *et al*, 1999; Subramaniam *et al*, 2005). In fact, IGF-1 can also inhibit ERK activation in some cell types, including neurons (Subramaniam *et al*, 2005). Therefore, the effect of IGF-1 on MAPK and cyclin D1 expression is not uniform for all cell types and most likely depends on the cell type, the nature, magnitude, and duration of the stimulus.

Protein tyrosine phosphatase 1B (PTP1B) was the first identified member of the classical tyrosine-specific protein phosphatase superfamily (Tonks *et al*, 1988), which dephosphorylates and thus inactivates the β chain of the IGF-1R (Buckley *et al*, 2002). PTP1B is localized at the cytoplasmic site of the endoplasmic reticulum (ER) (Frangioni *et al*, 1992). After translocation to the plasma membrane, it is endowed with the capacity to dephosphorylate plasma membrane-associated IGF-1R (Buckley *et al*, 2002; Yudushkin *et al*, 2007) and ligand-activated IGF-1R after endocytosis (Eden *et al*, 2010; Stuible *et al*, 2010). Similar to the activation of PTP1B, the lipid phosphatase and tensin homologue (PTEN) translocate from the cytosol to the plasma membrane, essential for its activation, and attache to the membrane through its C2 domain (Das *et al*, 2003; Leslie *et al*, 2003; Vazquez *et al*, 2006). Membrane-bound activated PTEN dephosphorylates membrane-anchored phosphatidylinositol 3,4,5-trisphosphate (PIP3), thus opposing PI3K action with subsequent attenuation of IGF-1 signalling (Maehama & Dixon, 1998).

Several lines of evidence suggest that reactive oxygen species (ROS), in particular H_2_O_2,_ interfere with insulin/IGF-1 signalling (Leslie *et al*, 2003; Houstis *et al*, 2006; Bashan *et al*, 2009; Loh *et al*, 2009; Finkel, 2011; Murphy *et al*, 2011). Both PTP1B and PTEN are regulated through reversible oxidation and inactivation (Leslie *et al*, 2003; Salmeen *et al*, 2003) and thus may be involved in enhanced IGF-1 signalling. PTP1B and PTEN contain a critical cysteine residue in their active site that exclusively in its reduced state is able to participate in substrate dephosphorylation. In fact, H_2_O_2_-mediated oxidation of cysteine inactivates these phosphatases *in vitro* (Gough & Cotter, 2011). In addition, it was reported that mice deficient of glutathione peroxidase (Gpx), the key enzyme responsible for H_2_O_2_ detoxification, were distinctly protected from high fat-induced insulin resistance (Loh *et al*, 2009). This study provides causal evidence for the enhancement of insulin signalling by H_2_O_2_ *in vivo,* suggesting that oxidative inactivation of PTP1B and PTEN stimulates IGF-1 signalling. In the physiological context, H_2_O_2_ is also generated following binding of growth factors to their receptors, thus promoting growth factor signalling by oxidative inactivation of phosphatases (Sundaresan *et al*, 1995).

By contrast to H_2_O_2_, superoxide anion radicals (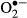) physiologically occur as a metabolic by-product during oxidative phosphorylation within mitochondria. Apart from a correlative report (Hoehn *et al*, 2009), their biology has not been studied in great detail under physiologic and pathologic conditions. An in-depth knowledge on the role of 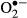 would, however, be particularly relevant as 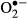 accumulates in a variety of cells during ageing (Treiber *et al*, 2011), neurodegeneration (Wu *et al*, 2010), and conditions such as the metabolic syndrome and diabetes (Kim *et al*, 2008). In addition, 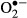 plays a central role in inflammatory conditions (Shishido *et al*, 1994; Naya *et al*, 1997; Cheng *et al*, 1999; MacArthur *et al*, 2000; Lu *et al*, 2003) and in chronic non-healing wounds (Sindrilaru *et al*, 2011).

Using complementary biochemical and genetic approaches to specifically dissect the effect of 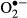 and H_2_O_2_ on IGF-1 signalling, we here found that by contrast to the H_2_O_2_-dependent inhibition of the key phosphatases PTP1B and PTEN, accumulation of mitochondrial 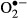 resulted in marked activation of PTP1B and PTEN, eventually dampening the IGF-1-induced signalling cascade at distinct steps of downstream effectors. This leads to subsequent inhibition of murine dermal fibroblast (MDFs) proliferation, changed expression of factors responsible for protein translation initiation, and reduced gene expression of α1 (I), α1 (III), and α2 (I) collagen chains, key features of skin ageing. The 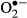-dependent IGF-1 resistance was significantly abrogated by pharmacologic and genetic inhibition of PTP1B and PTEN, suggesting a central role for these phosphatases in the 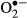-dependent IGF-1 resistance. This was confirmed *in vivo* by the rescue of the skin ageing phenotype and IGF-1 signalling in fibroblast-specific Sod2-deficient mice, where PTEN gene was heterozygously deleted in Sod2-deficient mice. Similarly, pharmacologic PTP1B inhibition in Sod2-deficient mice showed partial reversal of the impaired IGF-1 signalling. An advanced understanding of the involvement of distinct ROS species in the regulation of IGF-1 resistance holds substantial promise for prevention and therapy of age-related and other pathologies.

## Results

### Superoxide anions and hydrogen peroxide differentially regulate the IGF-1/AKT pathway

**Figure 1:**
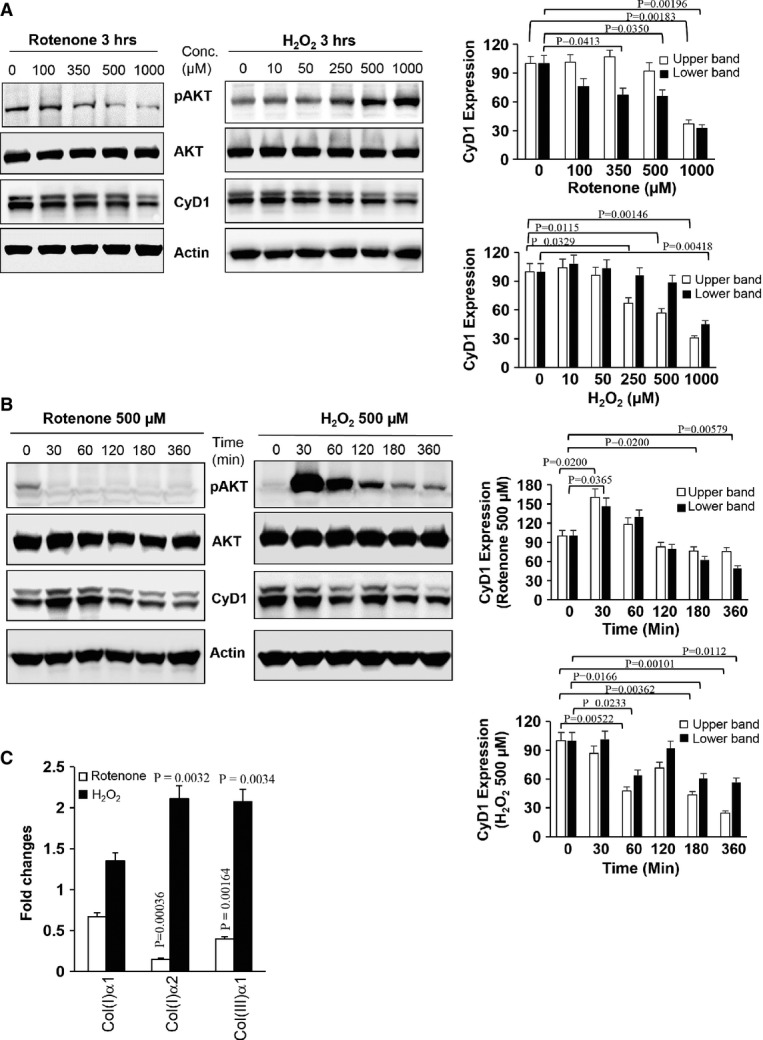
Superoxide anion radicals and H_2_O_2_ differentially regulate IGF-1 signalling To assess differential regulation of downstream key effector proteins of IGF-1 signalling such as activated (phosphorylated) AKT (pAKT), total AKT (AKT), and cyclin D1 (CyD1) levels in MDFs either treated with increasing rotenone concentrations (left panel) or with increasing H_2_O_2_ concentrations (right panel) for 3 h in serum-free DMEM, cell lysates were analysed by Western blotting equilibrated to actin expression levels. The graphs represent the densitometric analyses of cyclin D1 (CyD1) expression (mean ± SEM) in the presence of different concentrations of rotenone and H_2_O_2_. The upper band is the hyperphosphorylated cyclin D1, and the lower band represents the hypophosphorylated cyclin D1. Comparison was made with two-tailed *t*-test; respective comparison was indicated with line and *P*-value. The results are representative for three independent experiments (*n* = 3).MDFs were treated with 500 μM rotenone (left panel) or 500 μM H_2_O_2_ (right panel) for the indicated time periods, and the expression of IGF-1 downstream effector proteins (as in A) was analysed by Western blotting. The graphs represent the densitometric analyses of cyclin D1 (CyD1) expression in the presence of 500 μM rotenone and 500 μM H_2_O_2_ at the indicated time point. The values are presented as mean ± SEM in percentage, and comparison was made with two-tailed *t*-test; respective comparison was indicated with line and *P*-value. The results are representative for three independent experiments (*n* = 3).In order to dissect the effects of different reactive oxygen species on IGF-1 target genes in MDFs, mRNA expression of different type I and type III collagen chains (Col(I)α1, Col(I)α2, and Col(III)α1) was analysed by RT–qPCR in MDFs treated with either 500 μM rotenone or 500 μM H_2_O_2_ for 3 h (*n* = 6). Values are presented as mean ± SEM of fold change, and comparison was made with two-tailed *t*-test; *P*-value indicated the significance of difference, comparing the indicated collagen chain mRNA levels of either rotenone- or H_2_O_2_-treated MDFs with control. Source data are available online for this figure.

Murine dermal fibroblasts (MDFs) endogenously produce and release IGF-1, which via autocrine and paracrine mechanisms stimulate the IGF-1 signalling cascade (Yakar *et al*, 1999). To study the effect of different ROS species such as superoxide anion (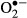) and hydrogen peroxide (H_2_O_2_) on the IGF-1/AKT axis, MDFs were treated with rotenone, an established inhibitor of complex I of the mitochondrial electron transport chain (Li *et al*, 2003) or with exogenous H_2_O_2_. Inhibition of complex I by rotenone increases electron leakage from this complex to the matrix side, finally leading to partial reduction of oxygen to 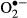 (Buetler *et al*, 2004) (Supplementary Fig S1A). As expected, rotenone concentration dependently increased mitochondrial 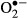 generation in MDFs assessed by MitoSOX, a specific indicator for mitochondrial 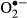 (Supplementary Fig S1B). We first analysed the phosphorylation of AKT as a critical major downstream signalling component following IGF-1R activation. Notably, 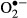 and H_2_O_2_ exerted opposite effects on AKT signalling in MDFs, irrespective of the rotenone and H_2_O_2_ concentration (Fig1A) and the time of exposure (Fig1B). H_2_O_2_ at moderate and high concentrations ranging from 10  to 1,000 μM markedly induced phosphorylation of AKT without exerting any effect on total AKT levels (Fig1A), a finding which is in agreement with published reports (Ushio-Fukai *et al*, 1999). Interestingly, rotenone resulted in a concentration-dependent reduction in AKT activation as shown by reduced phosphorylation of AKT at Ser 473 (pAKT) (Fig1A). At higher concentrations, both rotenone-induced 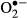 and H_2_O_2_ led to a marked reduction in the amount of cyclin D1 (Fig1A and B), which at physiological concentrations is required for cell cycle progression from the G1 phase (Winston *et al*, 1996). Collagen type I and III synthesis is dependent on IGF-1/AKT signalling and is mainly controlled at the mRNA level (Gillery *et al*, 1992). These collagen types produced by MDFs constitute the major extracellular matrix proteins of the skin, which significantly decrease during ageing of murine and human skin (Quan *et al*, 2010). Interestingly, enhanced 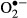 generation in MDFs at a rotenone concentration of 500 μM for 3 h inhibited the specific mRNA levels of the α1 and α2 chains of type I collagen and the α1 chain of type III collagen (Fig1C), while H_2_O_2_ at a concentration of 500 μM—parallel to enhancing IGF-1 signalling—increased α chains of type I and type III collagen-specific mRNA levels (Fig1C). Of note, more than 90% of MDFs were viable at rotenone and H_2_O_2_ concentrations of 500 μM, indicating that altered collagen synthesis was not related to toxicity (Supplementary Fig S1C). Collectively, these data demonstrate that 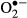 and H_2_O_2_ exert at least partly opposing effects on IGF-1/AKT signalling in MDFs.

### Enhanced superoxide anion concentrations induce partial IGF-1 resistance

**Figure 2:**
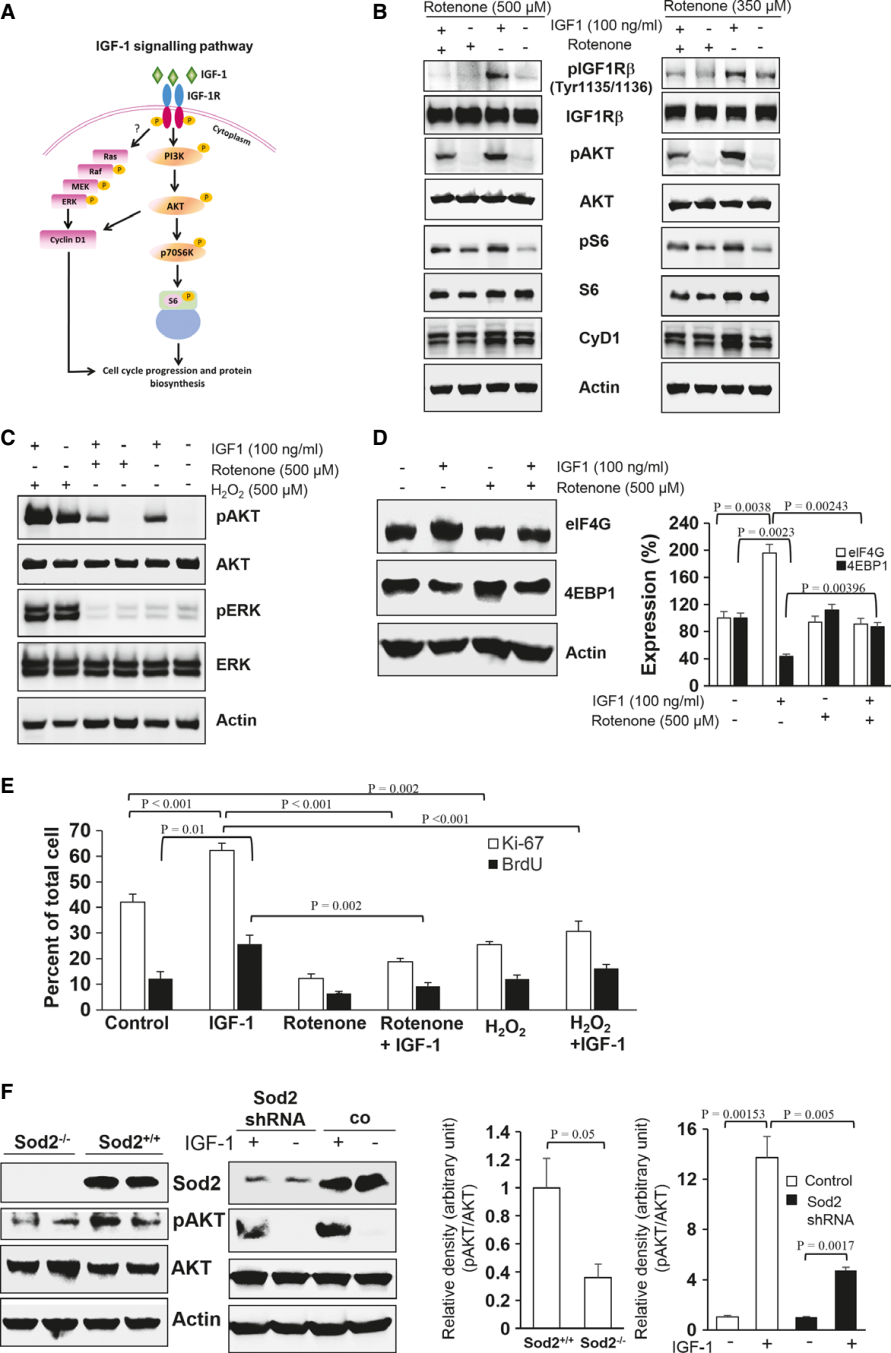
Enhanced superoxide anion concentrations suppress IGF-1 signalling and induce partial IGF-1 resistance Schematic representation of the IGF-1 signalling following IGF-1 binding to its receptor (IGF-1R). The Ras/Raf/MEK/ERK pathway regulates cyclin D1-dependent cell cycle progression, and the PI3K/AKT/p70S6K pathway regulates translation initiation, protein synthesis, and growth. Arrows (↓) indicate stepwise activation (phosphorylation) of IGF-1 downstream proteins as shown by “P” in the yellow circle. Currently, it is not resolved whether enhanced 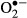 concentrations affect the Ras/MEK/ERK pathway and the PI3K/AKT pathway at the level of the IGF-1 receptor.MDFs in the presence or absence of 350 μM and 500 μM rotenone were either stimulated with 100 ng/ml IGF-1 or kept unstimulated for 3 h and were assessed for the expression of indicated IGF-1 downstream effector proteins in their phosphorylated (activated) states (pIGF-1Rβ (Tyr1135/1136), pAKT (S473), pS6 (S240/244)) and total (IGF-1Rβ, AKT, S6, and CyD1) by Western blotting.The effect of rotenone-induced 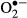 and H_2_O_2_ on IGF-1-stimulated AKT and ERK activation was studied by Western blot analyses with actin serving as a loading control.To dissect whether enhanced 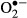 concentrations affect IGF-1-dependent effectors involved in the initiation of protein translation, in non-treated MDFs or MDFs treated with 100 ng/ml IGF-1 in the presence or absence of 500 μM rotenone for 3 h, protein levels of eIF4G, the gate keeper of protein translation initiation, and 4EBP1, the inhibitor of protein translation initiation, were studied by Western blot analyses with actin serving as a loading control. The graph represents densitometric analyses of eIF4G and 4EBP1 corrected with respective actin (loading control). Comparison was made with two-tailed *t*-test; respective comparison was indicated with a line and *P*-value.To study the role of 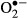 and H_2_O_2_ on cell proliferation, quantification of Ki-67 (white bars) and BrdU incorporation (black bars) was performed in non-treated MDFs (control), IGF-1- (100 ng/ml), rotenone- (100 μM), H_2_O_2_- (100 μM) and combined IGF-1/rotenone- or IGF-1/H_2_O_2_-treated MDFs for 12 h. Values are mean ± SEM of percent positive cells, and comparison was made with one-way ANOVA followed by Bonferroni correction.The suppressive action of enhanced 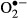 concentrations on IGF-1 signalling was confirmed in Sod2*-*deficient MDFs (left panel) and in shRNA-mediated Sod2-silenced MDFs (right panel). Western blot analyses revealed expression levels of the indicated IGF-1 downstream effector proteins in lysates obtained from Sod2*-*deficient and wild-type MDFs as well as MDFs transduced with lentiviral particles containing either the Sod2 shRNA or non-targeting shRNA plasmid. In Sod2 shRNA and non-targeting shRNA plasmid, experimental MDF groups were stimulated either with 100 ng/ml IGF-1 for 3 h or with vehicle. Graphs represent densitometric analyses of the ratio of pAKT and AKT corrected with respective actin (loading control) in Sod2-deficient cells (left graph) and shRNA-mediated Sod2-silenced MDFs (right graph). Comparison was made with two-tailed *t*-test; respective comparison was indicated with a line and *P*-value. Data information: In (B–D, F), representative Western blots out of three independent experiments (*n* = 3) are shown. Source data are available online for this figure.

Next, we analysed the IGF-1R sensitivity to its exogenous ligand in the presence of high 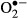 concentrations (Fig2A). Recombinant IGF-1 (IGF-1) was used at a concentration of 100 ng/ml to activate the IGF-1/AKT pathway in MDFs in the presence or absence of rotenone. Addition of IGF-1 resulted in the activation (phosphorylation) of IGF-1Rβ, AKT, ribosomal protein S6 (S6), and increased cyclin D1 levels in MDFs (Fig2B). Notably, rotenone at concentrations of 350 and 500 μM led to a significant inhibition of IGF-1-induced activation (phosphorylation) of AKT (pAKT) (Fig2B), without any alteration of total AKT and IGF-1Rβ levels. Rotenone treatment markedly inhibited IGF-1-mediated IGF-1Rβ phosphorylation (Fig2B), suggesting that enhanced 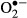 concentrations suppressed the IGF-1/AKT axis at the initial step of IGF-1-mediated IGF-1Rβ activation. IGF-1 has no effect on activation of ERK in MDFs at least at 60 min post-stimulation (Fig2C), although there are reports of ERK activation by IGF-1 in other cell types (Parrizas *et al*, 1997; Peruzzi *et al*, 1999). In fact, IGF-1 induces the expression of cyclin D1 (Fig2B), but does not induce the activation of ERK (Fig2C). The expression of cyclin D1, which is suppressed after IGF-1 treatment in the presence of rotenone (Fig2B) very much, suggests that cyclin D1 is not regulated by the IGF-1R-induced ERK phosphorylation, but rather by IGF-1-induced AKT phosphorylation. The observed rotenone (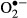)-mediated reduction of cyclin D1 expression is possibly due to the inhibition of other pathways or effectors downstream of IGF-1R (Winston *et al*, 1996). Of note, eIF4G, the protein essential for initiation of translation, was decreased, and 4EBP1, the counteracting inhibitor of the initiation process of translation (Kong & Lasko, 2012), was markedly increased in the presence of high 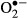 concentrations despite IGF-1 stimulation (Fig2D). These data imply that steps critically required for translation initiation and protein synthesis might be suppressed by 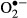 (Fig2D). In addition, rotenone-induced 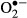 was found to suppress IGF-1-mediated cell proliferation, as indicated by reduced Ki67 and BrdU labelling (Fig2E and Supplementary Fig S2). Rotenone alone reduced basal cell proliferation, and additional supplementation with IGF-1 could only slightly enhance Ki67-positive cells and BrdU incorporation, indicating that 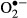 at higher concentrations overwhelmed the cell proliferation stimulatory effect of IGF-1 (Fig2E and Supplementary Fig S2). H_2_O_2_ at a concentration of 100 μM suppressed cell proliferation (Fig2E and Supplementary Fig S2), and IGF-1 only partly attenuated the inhibitory effect of H_2_O_2_ on basal cell proliferation (Fig2E and Supplementary Fig S2). MDFs isolated from Sod2 knockout mice, due to the lack of 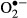 detoxification, displayed high intracellular 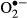 concentrations (Treiber *et al*, 2011). These MDFs revealed a marked reduction in pAKT compared to Sod2 competent murine MDFs (Fig2F, left panel). Lentivirus vector-based silencing of Sod2 in MDFs (Sod2 shRNA) also showed higher MitoSox fluorescence indicative of increased 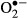 concentrations (Supplementary Fig S1D). Following IGF-1 treatment, Sod2-silenced MDFs showed partial IGF-1 resistance with lower pAKT levels when compared to IGF-1-treated Sod2 competent control MDFs (co) (Fig2F, right panel). Rotenone also induced partial IGF-1 resistance in murine keratinocytes (epidermal skin cells) (Supplementary Fig S3), suggesting that 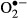-induced IGF-1 resistance is not restricted to MDFs. These data suggest that enhanced 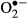 concentrations impair IGF-1 signalling eventually resulting in IGF-1 resistance.

### *In vivo* evidence for superoxide anion-induced partial IGF-1 resistance

**Figure 3:**
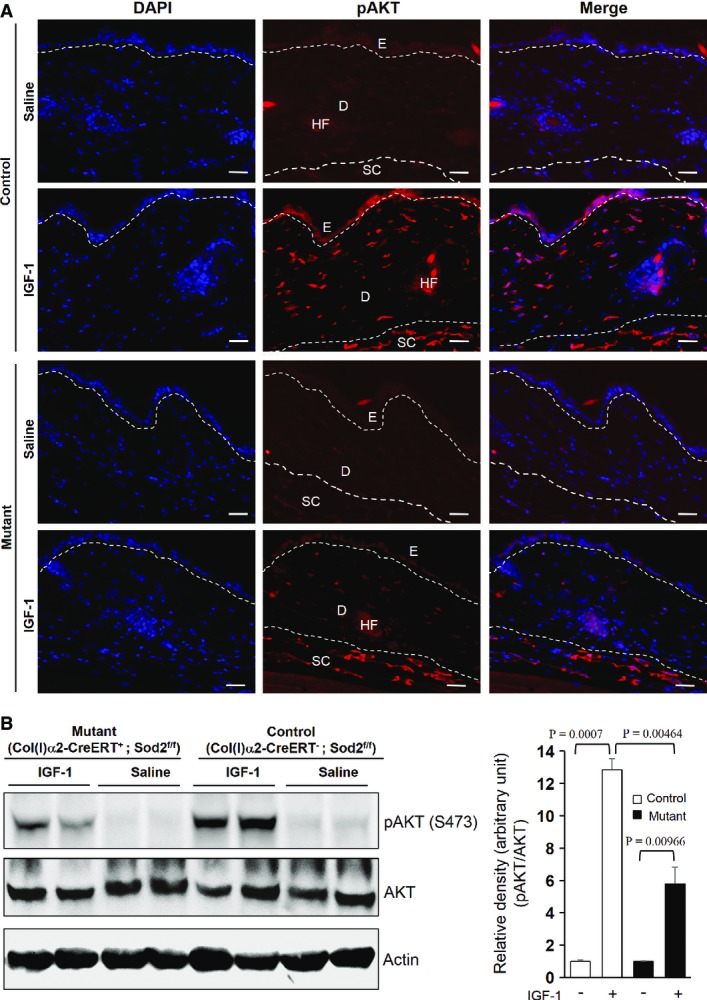
Impaired IGF-1 signalling in the skin of fibroblast-specific Sod2-deficient mice To study the impact of enhanced 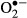 concentrations on IGF-1 signalling *in vivo*, representative skin sections of either saline- or IGF-1-treated Sod2 wild-type control (with normal 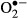 concentrations) and mutant mice (with enhanced 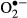 concentrations in fibroblasts) were stained for pAKT (S473) (red) and DAPI (blue, nuclei). Skin samples were collected 15 min after i.p. injection of 1 mg/ml IGF-1 or saline. Scale bars, 30 μm. E, epithelium; D, dermis; HF, hair follicle; SC, subcutaneous tissue, *n* = 5.To further confirm the suppressive effect of enhanced 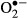 concentrations on IGF-1 signalling *in vivo*, skin lysates from control mice (with normal 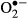 concentrations) and mutant mice (with enhanced 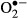 concentrations in fibroblasts) were prepared 15 min or after i.p. injection of 1 mg/ml IGF-1 or saline, and the expression of pAKT (S473) and AKT was analysed by Western blotting and equilibrated to actin expression levels. A representative Western blot out of three independent experiments is shown here. The graph (right panel) depicts densitometric analyses of pAKT/AKT ratio after correction with actin (loading control) for each of the four groups. Comparison was made with two-tailed *t*-test; respective comparison was indicated with a line and *P*-value. Source data are available online for this figure.

Connective tissue-specific inducible Sod2-deficient mice were used to study whether 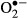-mediated partial IGF-1 resistance also occurs *in vivo*. *Sod2* floxed mice (Strassburger *et al*, 2005; Treiber *et al*, 2011) were crossed with transgenic mice expressing a fusion protein of Cre recombinase and a tamoxifen responsive element under the control of the fibroblast-specific collagen type I α2 promoter (Col(I)α2*-*CreERT mice) (Zheng *et al*, 2002) to generate Col(I)α2-CreERT^+^;Sod2^f/f^ mice (mutant) and Col(I)α2*-*CreERT^−^;Sod2^f/f^ mice (control with competent *Sod2* gene). Deletion of *Sod2* in the dermal compartment (connective tissue and fibroblast-rich part) of the skin was performed by administration of 4-OH tamoxifen (Supplementary Fig S4A and B). Both control and mutant mice were intraperitoneally (i.p.) injected either with 1 mg/kg recombinant IGF-1 or with normal saline. Indeed, IGF-1 treatment significantly induced the AKT phosphorylation (Ser 473) in the skin of control mice compared to saline-treated control mice as shown by immunofluorescence staining of skin sections and Western blot analyses of skin lysates (Fig3A and B). Strikingly, injection of IGF-1 only slightly induced AKT phosphorylation in the skin of mutant mice (with 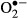 at higher concentrations) compared to saline-treated mutant mice. Of note, the extent of AKT phosphorylation was markedly decreased in the fibroblast-rich dermis but not in the adipocyte-containing subcutaneous layer of IGF-1-injected mutant mice compared to IGF-1-treated control mice (Fig3A). The maximum stimulatory effect of IGF-1 was found 15 min after injection, which was no longer detectable at 60 min after IGF-1 injection (Fig3B and Supplementary Fig S4C). As higher 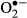 concentrations were selectively attained by fibroblast-specific deletion of *Sod2* (Treiber *et al*, 2011), these data underscore the specificity of our model and a previously unreported critical role of increased 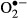 concentrations in suppressing the IGF-1/AKT axis *in vivo*.

### IGF-1 resistance is due to enhanced PTP1B and PTEN phosphatase activity

**Figure 4:**
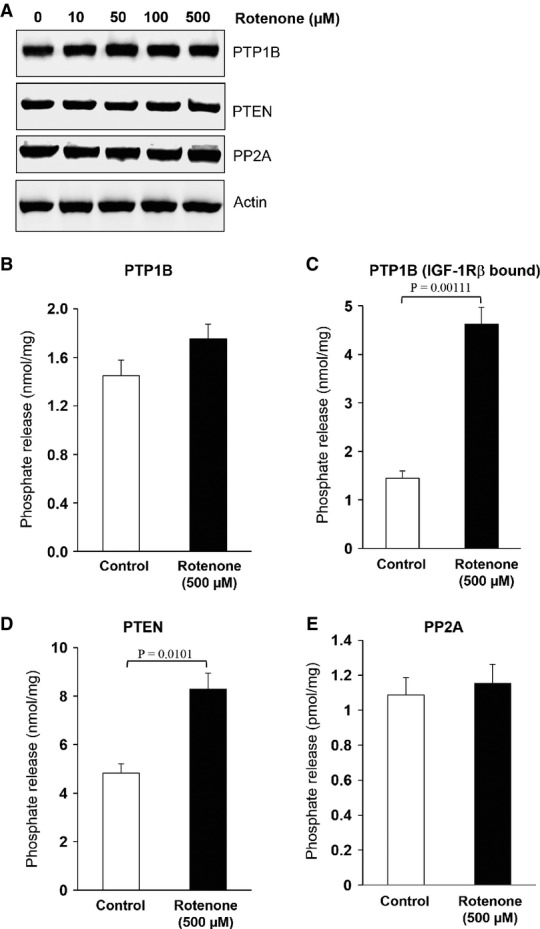
Enhanced superoxide anion flux inhibits tyrosine phosphorylation of IGF-1Rβ through PTP1B and PTEN activation A Protein levels of PTP1B, PTEN, and PP2A were examined by immunoblotting of lysates derived from MDFs treated with the indicated concentrations of rotenone for 3 h. A representative Western blot is shown out of three independent experiments with similar results.B–E Phosphatase activity of (B) PTP1B, (C) IGF-1Rβ-associated PTP1B, (D) PTEN, and (E) PP2A were determined in lysates of MDFs treated with 500 μM rotenone for 3 h. Only PTP1B bound to IGF-1Rβ in the plasma membrane and PTEN, but not total PTP1B and PP2A, revealed significant dephosphorylating activity. Values are mean ± SEM of nmol phosphate release per mg protein, and comparison was made with two-tailed *t*-test (*n* = 6); respective comparison was indicated with a line and significance with *P*-value.

**Figure 5:**
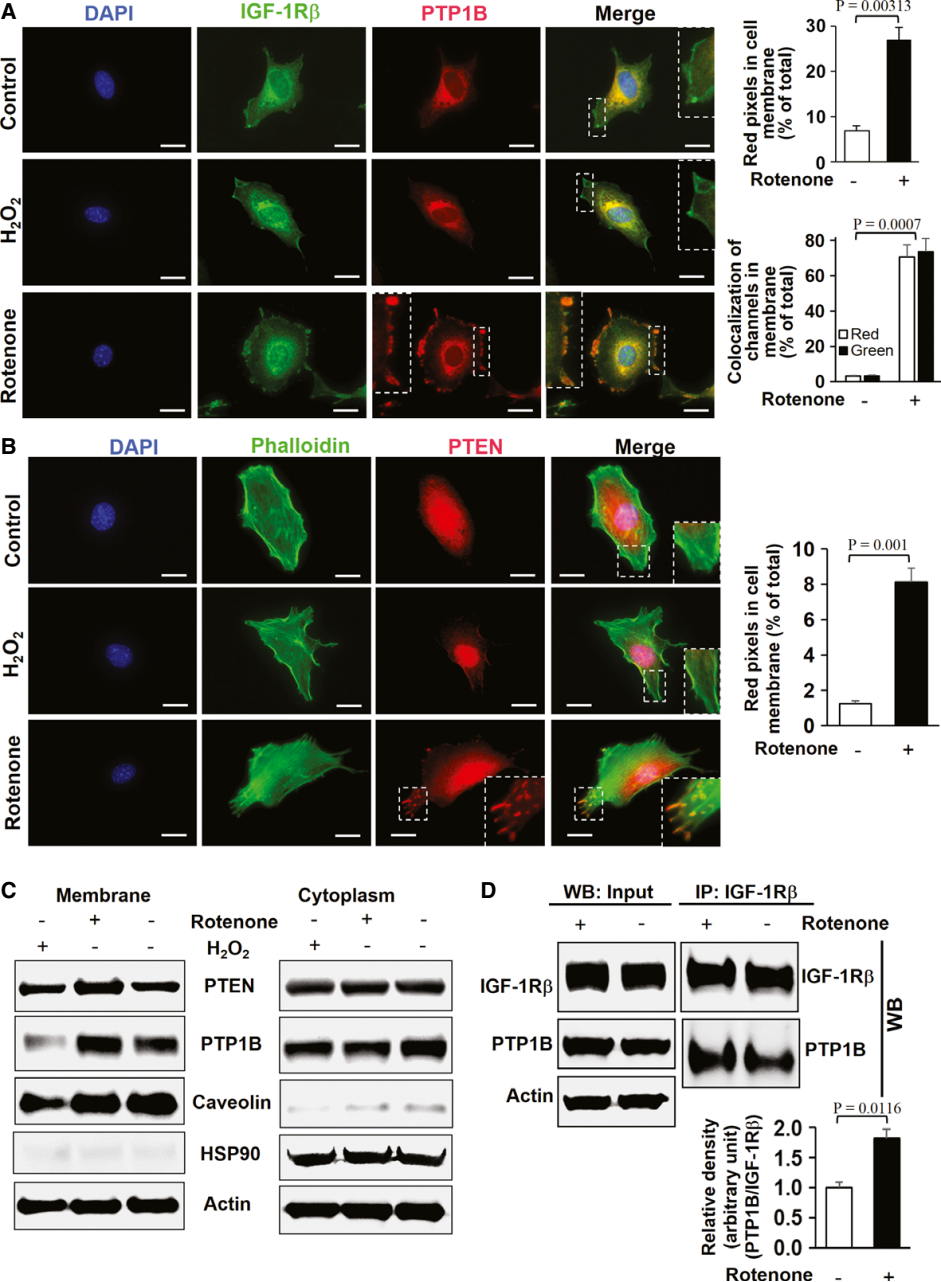
Superoxide anions specifically promote enrichment of PTP1B and PTEN in the plasma membrane fraction Representative immunostaining of IGF-1R (green) and PTP1B (red) in MDFs treated with 500 μM H_2_O_2_ and 500 μM rotenone for 3 h. Nuclei were stained with DAPI (blue). The insets show an enlarged view of the area indicated by the dotted rectangles. Scale bars, 50 μm. The upper graph shows the amount of red pixels that represent PTP1B in the cell membrane regions of both control and rotenone-treated murine dermal fibroblast (MDFs) presented as percentage of total red pixels of total cells. The lower graph depicts the amount of colocalized red (PTP1B) and green (IGF-1R) pixels at the cell membrane in control and rotenone-treated MDFs presented as percentage of total red pixel or green pixel. Values are mean ± SEM in percent, and comparison was made with two-tailed *t*-test (*n* = 5); respective comparison was indicated with line and *P*-value.Representative immunostaining of phalloidin (green) and PTEN (red) in MDFs treated with 500 μM H_2_O_2_ and 500 μM rotenone for 3 h. Nuclei were stained with DAPI (blue). The insets show an enlarged view of the area indicated by the dotted rectangle. Scale bars, 50 μm. The graph depicts the amount of red pixels (PTEN) in the cell membrane regions of both control and rotenone-treated MDFs presented as percent of total red pixels of total cell. Values are mean ± SEM in percent, and comparison was made with two-tailed *t*-test (*n* = 5); respective comparison was indicated with a line and *P*-value.Assessment of the PTEN and PTP1B expression in the membrane (left panel) and cytoplasmic fractions (right panel) from MDFs treated with 500 μM H_2_O_2_ and 500 μM rotenone for 3 h by Western blotting equilibrated to actin expression levels. The purity of the membrane fractions was confirmed by the enrichment of the membrane-associated protein caveolin in the absence of the cytoplasmic HSP90 protein, with the vice versa distribution in the cytoplasmic fractions. Unlike treatment with H_2_O_2_, rotenone led to an enrichment of PTEN and PTP1B in the membrane fraction. Representative experiments out of three independent experiments are shown.To further confirm the direct physical interaction of IGF-1Rβ and PTP1B, MDFs were treated with 500 μM rotenone for 3 h; thereafter, the cell lysates were immunoprecipitated (IP) with anti-IGF-1Rβ antibodies followed by immunoblotting with anti-IGF-1Rβ and anti-PTP1B antibodies. Densitometric analysis depicts relative ratios of PTP1B/IGF-1Rβ for control and rotenone-treated groups. Comparison was made with two-tailed *t*-test (*n* = 3), and respective comparison between the groups is indicated by a line and *P*-value. Source data are available online for this figure.

**Figure 6:**
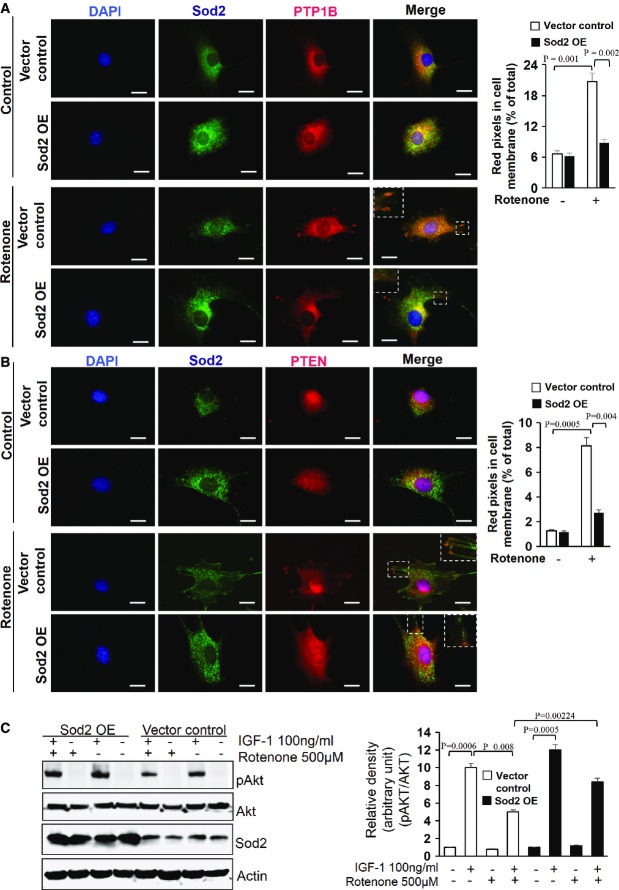
Decreased membrane localization of PTP1B and PTEN in rotenone-treated Sod2-overexpressing murine fibroblasts To assess whether Sod2 overexpression with enhanced 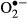-dismutating capacity can suppress PTP1B membrane translocation, MDFs transfected with either the vector control or the Sod2 overexpression vector (Sod2 OE) were cultured in the absence (Control) and presence of 500 μM rotenone (Rotenone) for 3 h. Representative immunostainings of Sod2 (green) and PTP1B (red) are depicted in the differently treated experimental groups. Nuclei were stained with DAPI (blue). Scale bars, 50 μm. The graph shows the quantification of red pixels that represent PTP1B in the cell membrane regions of both control and rotenone-treated murine dermal fibroblast (MDFs) with either vector control or Sod2 overexpression vector (SOD2 OE). Data are presented as percent of total red pixels of total cells. The data were analysed by two-tailed *t*-test (*n* = 5), and respective comparison was indicated by a line and *P*-value.The identical experimental setting as described in (A) was used, and the PTEN membrane translocation was studied under high 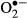-dismutating capacity of Sod2-overexpressing MDFs (Sod2 OE) and MDFs with normal 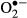-dismutating capacity (vector control) in the absence (Control) and presence of rotenone (Rotenone). Representative microphotographs from immunostainings for Sod2 (green) and PTEN (red) in differently treated MDF groups. Nuclei were stained with DAPI (blue). Scale bars, 50 μm. The graph shows the quantification of red pixels that represent PTEN in the cell membrane regions of both control and rotenone-treated murine dermal fibroblast (MDFs) with either vector control or Sod2 overexpression vector (SOD2 OE) presented as percent of total red pixels of total cells. In both, the data were analysed by two-tailed *t*-test (*n* = 5) and respective comparison was indicated by a line and *P*-value.To further confirm that Sod2 overexpression with enhanced 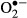-dismutating capacity can rescue the IGF-1 resistance at high 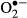 concentrations, MDFs transfected with either the vector control or the Sod2 overexpression vector (Sod2 OE) were cultured in the absence or presence of 500 μM rotenone for 3 h. Activation (phosphorylation) of the IGF-1 downstream effector protein pAKT (S473) compared to total AKT expression levels and the expression of Sod2 in lysates from MDFs of the differentially treated groups were analysed by Western blotting equilibrated to actin levels. The shown Western blot is representative of three independent experiments. Right panel shows the respective densitometric analyses, and the data were analysed by two-tailed *t*-test; respective comparison was indicated with a line and *P*-value. Source data are available online for this figure.

As we have observed that rotenone inhibited IGF-1-induced autophosphorylation of tyrosine residues (Tyr 1135 and 1136) of IGF-1Rβ (Fig2B), we further explored the possibility whether the activation of specific tyrosine phosphatases is responsible for the observed IGF-1Rβ dephosphorylation. To achieve this, we investigated the role of key phosphatases including PTP1B, PTEN, and PP2A involved in the regulation of IGF-1 signalling (Maehama & Dixon, 1998; Millward *et al*, 1999; Buckley *et al*, 2002). No significant change was observed in basal expression levels of PTP1B, PTEN, and PP2A phosphatases of MDFs subjected to different rotenone concentrations compared with non-treated MDFs (Fig4A). Also PTP1B activity was not enhanced in whole-cell lysates prepared from rotenone-treated MDFs (Fig4B). Interestingly, increased activities of PTP1B bound to its substrate (IGF-1Rβ) and PTEN occurred in rotenone-treated MDFs compared with non-treated MDFs (Fig4C and D), while no change was observed in PP2A activity (Fig4E). This suggests an important role for PTP1B and PTEN in the 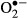-mediated suppression of the IGF-1/AKT axis. As membrane localization of these phosphatases is an essential prerequisite for the inactivation of IGF-1Rβ and downstream effectors of the IGF-1 signalling pathway (Buckley *et al*, 2002; Yudushkin *et al*, 2007), we analysed whether upon rotenone or H_2_O_2_ treatment, PTP1B and PTEN translocate to the plasma membrane from the endoplasmic reticulum and cytoplasm, respectively. Notably, PTP1B and PTEN were recruited to the plasma membrane when MDFs were subjected to rotenone (Fig5A and B and corresponding inserts), whereas no such translocation to the plasma membrane was observed in non-treated control and H_2_O_2_-treated cells (Fig5A and B) Up to fourfold more PTP1B and PTEN were translocated to the membrane of rotenone-treated cells (Fig5A and B, right panels). Western immunoblot analyses further confirmed the enrichment of PTEN and PTP1B in the membrane fraction of rotenone-treated MDFs compared to non-treated controls and H_2_O_2_-treated MDFs (Fig5C and Supplementary Fig S5A and B). Using co-immunoprecipitation (Co-IP) experiments with IGF-1Rβ antibodies, a physical interaction of PTP1B with IGF-1Rβ was found after rotenone-induced 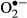 stress (Fig5D), and, as earlier shown, IGF-1Rβ-bound PTP1B displayed higher phosphatase activity in rotenone-treated MDFs (Fig4D). Furthermore, transient overexpression of Sod2 with increased 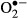 scavenging capacity (Supplementary Fig S1E) significantly inhibited rotenone-induced membrane translocation of PTP1B and PTEN in MDFs (Fig6A and B), suggesting a causal contribution of enhanced 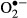 concentrations in the translocation of these two phosphatases to the plasma membrane. The fact that overexpression of Sod2 also improved IGF-1 sensitivity in rotenone-treated MDFs further supports a critical role of enhanced 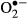 concentrations in the activation of these phosphatases with subsequent IGF-1 suppression (Fig6C). Stimulated by earlier reports that the phosphorylation of serine and threonine residues in the C-terminal domain of PTEN restricts it to the cytoplasm, while dephosphorylation favours its membrane localization and activation (Vazquez *et al*, 2006; Rahdar *et al*, 2009), we studied whether high 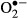 concentrations had any impact on the phosphorylation status of PTEN. For this purpose, PTEN was immunoprecipitated from the cytoplasmic and membrane fractions of vehicle or rotenone-treated MDFs followed by immunoblotting with antibodies against phospho-serine, phospho-threonine, and phospho-tyrosine. Interestingly, a membrane translocation phosphorylation pattern was found with reduced serine phosphorylation in cytoplasmic and membrane PTEN in rotenone-treated MDFs compared to vehicle-treated MDFs, while no threonine phosphorylation was detected in the membrane fraction of PTEN in both rotenone-treated and control MDFs (Supplementary Fig S6). In the cytoplasmic PTEN fraction, rotenone-treated MDFs showed lower threonine phosphorylation (Supplementary Fig S6). Of note, enhanced tyrosine phosphorylation in the membrane PTEN pool and reduced tyrosine phosphorylation in the cytosolic PTEN were found in rotenone-treated compared to vehicle-treated MDFs (Supplementary Fig S6). These data indicate that the 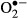-dependent PTEN translocation from the cytoplasm to the membrane correlates with specific post-translational modifications, the precise nature of which awaits elucidation. Taken together, the 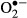-dependent PTP1B activation mediates IGF-1/AKT suppression at the step of IGF-1Rβ dephosphorylation, while PTEN recruitment and activation at the plasma membrane enhance the dephosphorylation of its phospholipid substrate PIP3 to PIP2, thus suppressing the phosphorylation (activation) of AKT.

### Inhibition of PTP1B and PTEN rescues the superoxide anion-induced IGF-1 resistance

**Figure 7:**
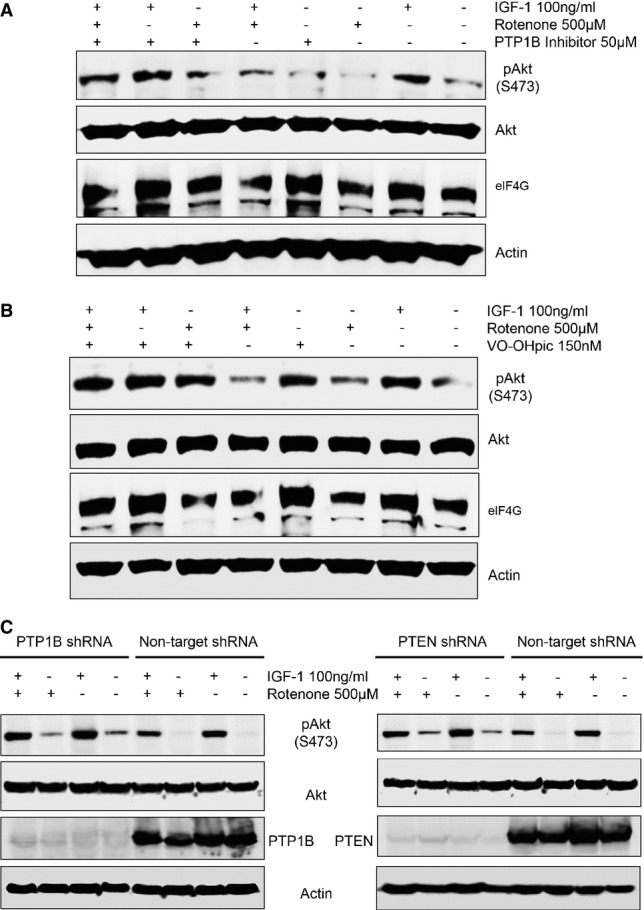
Pharmacologic and genetic inhibition of PTEN and PTP1B rescue IGF-1 sensitivity in enhanced superoxide anion-exposed cells To assess the specificity of 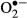-dependent PTP1B activation with subsequently suppressed IGF-1 signalling, the levels of IGF-1 downstream effectors, such as pAKT (S473) and AKT, and eIF4G, the translation initiation protein, were determined by Western blotting of lysates from MDFs treated either individually or in combination with 100 ng/ml IGF-1, 50 μM PTP1B inhibitor, and 500 μM rotenone as indicated for 3 h. The representative experiment shown here is one of two independent experiments performed.The identical experimental setting was used as described in (A) apart from using the specific PTEN inhibitor VO-OHpic (150 nM) instead of the PTP1B inhibitor. Similar to the PTP1B inhibitor in (A), the PTEN inhibitor almost completely alleviates the 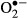-dependent suppression of the IGF-1 signalling. The representative experiment shown here is one of two independent experiments performed.To further confirm the role of 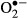-dependent PTP1B and PTEN activation on subsequent suppression of IGF-1 signalling, Western blot analyses for pAKT (S473), AKT, PTEN, and PTP1B expression in lysates from cells lentivirally transduced with the PTP1B-silencing shRNA (left panel), PTEN silencing shRNA (right panel), or with a scrambled non-targeting shRNA plasmid (control) treated with 100 ng/ml IGF-1 in the absence or presence of 500 μM rotenone for 3 h were performed with actin serving as a loading control. This representative Western blot is one of three independent experiments (*n* = 3) with similar results. Source data are available online for this figure.

**Figure 8:**
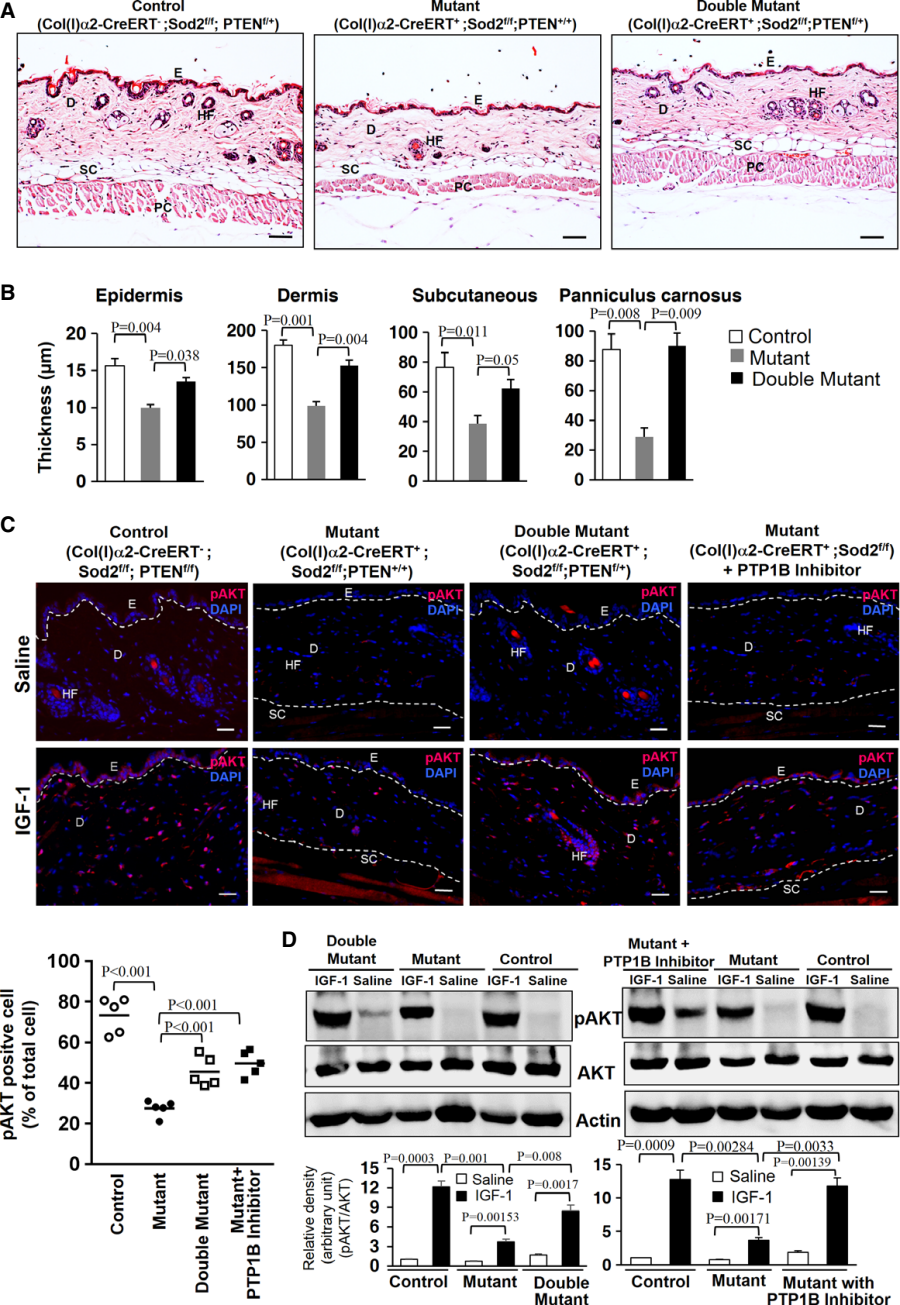
Inhibition of PTEN and PTP1B rescues skin atrophy and attenuates impaired IGF-1 signalling in fibroblast-specific Sod2-deficient mice To assess the long-term effect of partial PTEN attenuation on the skin atrophy phenotype, representative skin sections from control (Col(I)α2-CreERT^−^;Sod2^f/f^;PTEN^f/f^), mutant (Col(I)α2-CreERT^+^;Sod2^f/f^; PTEN^+/+^), and double mutant (Col(I)α2-CreERT^+^;Sod2^f/f^; PTEN^f/+^) mice were stained with haematoxylin–eosin and analysed for skin architecture. Of note, skin atrophy of fibroblast-specific Sod2-deficient mutant mice (Col(I)α2-CreERT^+^;Sod2^f/f^; PTEN^+/+^) was partially rescued in the double mutant with heterozygous PTEN deficiency and Sod2 deficiency (Col(I)α2-CreERT^+^;Sod2^f/f^; PTEN^f/+^). E, epidermis; D, dermis; HF, hair follicle; SC, subcutaneous fat; PC, panniculus carnosus. Dashed line indicates the epidermal–dermal junction. Scale bars, 30 μm.The vertical thickness of different skin layers, such as epidermis, dermis, subcutaneous fat, and panniculus carnosus, of mice with different genotypes (control, mutant, and double mutant) were measured from skin histologies of five mice per group (*n* = 5) and depicted as shown. Values are mean ± SEM of thickness in μm, and comparison was made with one-way ANOVA, followed by multiple comparison Bonferroni *t*-test; respective comparison was indicated by a line and *P*-value.To study the causal relationship of PTEN and PTP1B in 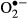-mediated IGF-1 resistance *in vivo*, representative skin sections of either saline or IGF-1-treated Sod2 control (with normal 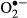 concentrations), mutant mice (with enhanced 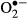 concentrations in fibroblasts, due to lack of Sod2), double mutant mice (with enhanced 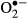 concentrations due to lack of Sod2 and heterozygous deficiency of PTEN in fibroblasts), and mutant mice with PTP1B inhibition (mice with Sod2 deficiency were treated with PTP1B inhibitor) were stained for pAKT (S473) (red) and DAPI (blue, nuclei). Skin samples were collected 15 min after i.p. injection of 1 mg/ml IGF-1 or saline. E, epithelium; D, dermis; HF, hair follicle; SC, subcutaneous tissue. Scale bars, 30 μm. The graph (left lower panel) represents the quantification of pAKT-positive cells (percent of total cells) in the dermal skin compartment of respective experimental groups (five mice per group, *n* = 5). Values are mean ± SEM of thickness in μM, and comparison was made with one-way ANOVA, followed by multiple comparison Bonferroni *t*-test; respective comparison was indicated with a line and *P*-value.To further substantiate the protective effect of PTEN and PTP1B suppression on 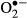-dependent impaired IGF-1 signalling *in vivo*, skin lysates from control mice and mutant mice and either double mutant mice (Sod2 homozygous and PTEN heterozygous deficient) or mutant mice (Sod2 homozygous deficient) treated with PTP1B inhibitor were prepared 15 min after i.p. injection of 1 mg/ml IGF-1 or saline, and the expression of pAKT (S473) and AKT was analysed by Western blotting. A representative Western blot out of three independent experiments (*n* = 3) is shown here. Densitometric analyses of pAKT/AKT ratio after correction with actin (loading control) for each of the groups. The comparison between two respective groups was performed by two-tailed *t*-test; respective comparison was indicated with a line and *P*-value. Source data are available online for this figure.

To further strengthen the causal role of PTP1B and PTEN in the 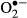-dependent IGF-1 resistance, we used pharmacological inhibitors and specific gene silencing and studied the phosphorylation state of AKT, a key step in IGF-1 downstream signalling. Notably, the PTP1B inhibitor (3-(3,5-Dibromo-4-hydroxy-benzoyl)-2-ethyl-benzofuran-6-sulfonicacid-(4-(thiazol-2-ylsulfamyl)-phenyl)-amide) at a concentration of 50 μM in part rescued the suppression of AKT phosphorylation and reduced eIF4G levels in MDFs exposed to recombinant IGF-1 in the presence of rotenone (Fig7A). In addition, the PTP1B inhibitor opposed rotenone-induced suppression of IGF-1Rβ phosphorylation in MDFs stimulated with IGF-1 (Supplementary Fig S7). Similarly, the suppressive action of high 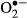 concentrations on IGF-1-induced AKT phosphorylation was rescued by 150 nM VO-OHpic, a vanadium-based specific PTEN inhibitor (Rosivatz *et al*, 2006) (Fig7B). In addition, silencing of PTP1B or PTEN using lentivirus-mediated shRNA expression significantly alleviated the suppressed activation state (phosphorylation) of AKT in MDFs treated with IGF-1 in the presence of rotenone (Fig7C). These data strongly suggest that enhanced activation of PTP1B and PTEN is causally involved in the 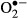-dependent IGF-1 resistance. To further explore whether our *in vitro* observations may also have relevance *in vivo* for tissue homeostasis, we used a genetic approach. Deletion of Sod2 in fibroblasts of the connective tissue-rich skin resulted in skin atrophy, a key feature of ageing, with reduced thickness of all the skin layers (Fig8A and B). Heterozygous deficiency of PTEN in the Sod2-deficient fibroblasts as achieved by crossing PTEN floxed mice with Col(I)α2-CreERT transgenic and Sod2 floxed mice (Col(I)α2CreERT^+^;Sod2^f/f^) followed by activation of CreERT with tamoxifen treatment (Supplementary Fig S8A and B) rescued skin atrophy/ageing in the double mutant mice (Col(I)α2-CreERT^+^;Sod2^f/f^;PTEN^f/+^) compared with Sod2 mutant mice (Col(I)α2CreERT^+^;Sod2^f/f^;PTEN^+/+^) (Fig8A and B). Of note, genetic heterozygous deficiency of PTEN with reduced PTEN expression or inhibition of PTP1B by specific inhibitor also attenuated the IGF-1 resistance *in vivo* in a Sod2-deficient murine ageing model (Fig8C and D). Accordingly, heterozygous PTEN deletion rescued IGF-1-stimulated phosphorylation of AKT in the skin of fibroblast-specific Sod2-deficient mice (Fig8C and D). Similarly, continuous inhibition of PTP1B with its specific inhibitor at a dose of 15 mg/kg for 5 days also rescued IGF-1-induced phosphorylation of AKT in the skin of fibroblast-specific Sod2-deficient mice (Fig8C and D). These data show that enhanced 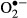
- concentrations in dermal fibroblasts in the skin *in situ*—via PTEN and PTP1B activation—dampen the IGF-1 signalling also *in vivo* and most likely contribute to skin ageing/atrophy.

## Discussion

**Figure 9:**
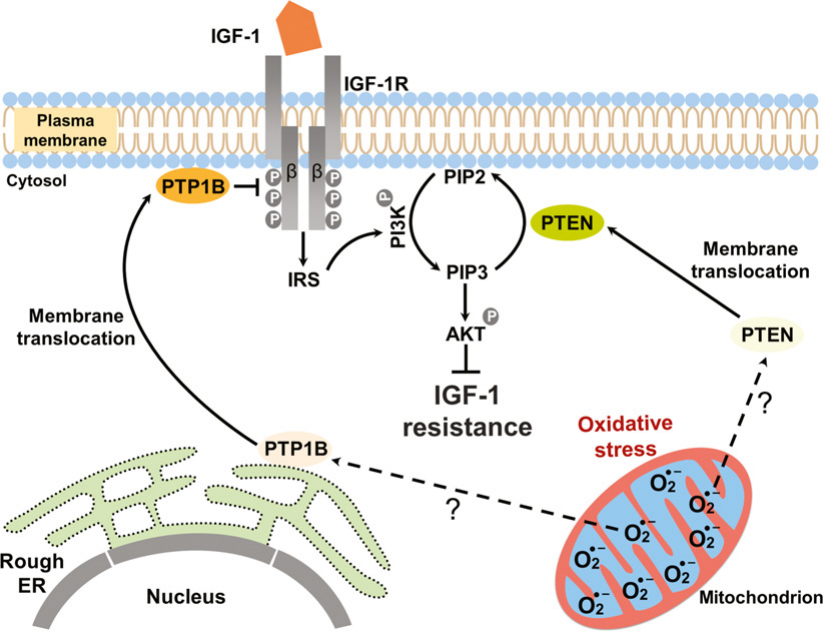
Enhanced mitochondrial superoxide anion concentrations repress IGF-1 signalling through concomitant PTP1B and PTEN activation Enhanced 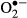 generation in mitochondria and possibly intermediates thereof are responsible for the translocation of PTP1B and PTEN from the ER or cytoplasm, respectively, to their membrane-anchored substrates, a prerequisite for their enzymatic action. Enhanced PTP1B activity dephosphorylates the IGF-1 receptor β chain at the plasma membrane, thus reducing its kinase activity. Similarly, enhanced 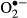 concentrations induce PTEN translocation and activation with subsequent reduction of PIP3 levels at the plasma membrane, which in turn inhibits the phosphorylation (activation) of the downstream effector AKT. Taken together, enhanced 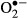 concentrations suppress IGF-1 signalling, thus promoting IGF-1 resistance.

The major finding of this report is that accumulation of mitochondrial 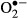 led to IGF-1 resistance by tilting the delicate balance of regulatory kinases and phosphatases towards enhanced activities of PTP1B and PTEN, key phosphatases of the IGF-1 signalling pathway. Enhanced activities of PTEN and PTP1B were associated with their membrane translocation. To the best of our knowledge, this finding identifies a previously unreported role of PTP1B and PTEN as molecular links mediating repression of IGF-1 signalling at high superoxide anion (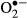) concentrations in the mammalian system (Fig9). The resulting IGF-1 resistance led to reduced fibroblast proliferation, marked suppression of specific α1 (I), α2 (I), and α1 (III) collagen chain mRNA levels, and changed expression of key factors responsible for translation initiation, collectively representing signature events of ageing and organ atrophy. Notably, genetic and pharmacological inhibition of PTP1B and PTEN alleviated IGF-1 resistance under conditions of enhanced 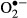 concentrations underlining their causal role and making PTP1B and PTEN potential targets for drug development. The finding that ImpL2, the *Drosophila* ortholog of insulin-like growth factor-binding protein 7 (IGFBP7), antagonizes insulin signalling in response to mitochondrial complex I perturbation (Owusu-Ansah *et al*, 2013) further underscores an evolutionarily conserved linkage between mitochondrial dysfunction and aberrant insulin signalling.

Enhanced 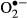 concentrations have been reported in several physiological and pathological inflammatory conditions (Shishido *et al*, 1994; Naya *et al*, 1997; Cheng *et al*, 1999; MacArthur *et al*, 2000; Lu *et al*, 2003; Liu *et al*, 2004; Sindrilaru *et al*, 2011). In addition, enhanced 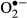 concentrations were also detected in senescent dermal fibroblasts *in vitro* and in the fibroblast from connective tissue-specific Sod2-deficient mice (Treiber *et al*, 2011) and—via dampening IGF-1 signalling—may contribute to ageing phenotypes of connective tissue-rich organs such as skin, bone, and muscle. This notion is consistent with the finding that these organs are particularly dependent on balanced IGF-1 signalling to maintain tissue homeostasis (Govoni *et al*, 2007a,b). Enhanced 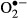 concentrations, as shown herein, suppressed fibroblast proliferation and the synthesis of interstitial collagen chains, the major extracellular matrix proteins of the skin. In fact, suppression of collagen synthesis and deposition as well as reduced proliferation constitute key molecular events in skin ageing (Fisher *et al*, 2008; Quan *et al*, 2010). Of note, IGF-1 was earlier reported to stimulate collagen synthesis and fibroblast growth (Gillery *et al*, 1992), and—if IGF-1 is suppressed by 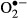-induced oxidative stress—results in skin ageing and atrophy. In addition, chronic venous leg ulcers and diabetic ulcers reveal a persistent inflammation with enhanced activation of pro-inflammatory macrophages, and an unrestrained release of 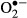, which at persistent high concentrations, overruns the 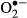-dismutating capacity of non-enzymatic and enzymatic antioxidants (Sindrilaru *et al*, 2011). Hence, 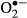 is the prevailing reactive oxygen species in chronic wounds and according to the herein reported data may dampen the signalling of IGF-1 and other growth factors and impair tissue repair processes. This notion is consistent with previous reports that many clinical trials with recombinant growth factors for the treatment of chronic wounds either failed or required extremely high concentrations of recombinant growth factors for substitution, indicating growth factor resistance of chronic wounds (Bitar & Al-Mulla, 2012). This poor outcome may at least in part be due to an 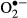-dependent enhanced phosphatase activation with subsequent attenuation of growth factor signalling.

In this study, we were interested in the role of distinct ROS such as mitochondria-derived 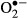 and H_2_O_2_ on IGF-1 sensitivity and downstream signalling. Strikingly, as opposed to the established H_2_O_2_-mediated enhanced insulin/IGF-1 sensitivity (Loh *et al*, 2009), we found that high 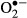 concentrations following rotenone-mediated inhibition of mitochondrial complex I or by silencing of 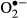-dismutating mitochondrial Sod2 resulted in IGF-1 resistance. Indeed, we identified enhanced activities and membrane localization of PTP1B and PTEN following high 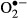 load, and interestingly, this effect was abolished by overexpression of Sod2. IGF-1 resistance was also observed in Sod2-deficient fibroblasts with significant accumulation of mitochondrial 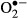, but not of H_2_O_2_ as proven by ROS-specific quantitative techniques (Treiber *et al*, 2011). Moreover, scavenging of mitochondrial 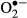 by the overexpression of Sod2 in the mitochondria of rotenone-treated murine dermal fibroblasts attenuated the observed IGF-1 resistance. Rotenone may not only suppress complex I of the electron transfer chain to enhance intramitochondrial 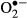 concentrations. From a previous publication, rotenone has been implicated in enhancing the NADPH oxidase in microglial cells of the brain with enhanced 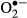 release into the extracellular space with subsequent neuron degeneration (Gao *et al*, 2003). Even though we cannot exclude this or other rotenone effects in our study, we provide consistent evidence that the rotenone-mediated inhibition of complex I with accumulation of high 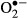 concentrations or 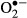 accumulation by silencing of 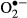-dismutating mitochondrial Sod2 resulted in IGF-1 resistance.

Our conclusion to have identified a novel route of 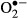 signalling with an 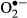-dependent increase of PTP1B and PTEN activity is based on several lines of evidence. First, in the case of PTP1B, we found a translocation of PTP1B from the ER to plasma membranes and co-localization with IGF-1Rβ. This translocation is indicative of PTP1B activation (Buckley *et al*, 2002; Yudushkin *et al*, 2007). Secondly, using co-immunoprecipitation, a physical interaction of PTP1B with the membrane-associated IGF-1Rβ and higher phosphatase activity of the IGF-1Rβ-bound PTP1B fraction was observed further supporting our hypothesis and suggesting a key role of PTP1B in 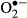-driven repression of IGF-1 signalling. Finally, inhibition of PTP1B either by a pharmacological inhibitor or through shRNA-mediated knockdown reversed the 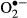-induced IGF-1 resistance. The conclusion that PTEN activity was also increased at high 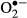 concentrations is supported by the following results. First, translocation of PTEN from the cytosol to the plasma membrane indicating PTEN activation (Das *et al*, 2003; Vazquez *et al*, 2006; Rahdar *et al*, 2009) was consistently observed in fibroblasts exposed to enhanced 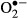 but not to enhanced H_2_O_2_ concentrations. Second, higher activity of PTEN was found in rotenone-treated MDFs. Third, the PTEN-specific inhibitor, VO-OHpic, significantly attenuated the 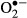-dependent suppression of IGF-1 signalling. VO-OHpic is a highly selective inhibitor for PTEN and only inhibits other PTPs at concentrations within the high micromolar range (Rosivatz *et al*, 2006). Fourth, similar results were also observed upon shRNA-mediated PTEN silencing. Importantly, partial deletion of PTEN and inhibition of PTP1B in Sod2-deficient mice also rescued the IGF-1 resistance and the atrophy/ageing skin phenotype. Thus, repression of IGF-1 signalling by a high 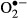 load is predominantly mediated by the active participation of PTP1B and PTEN. Taken together, our results unify and explain earlier reports on increased IGF-1 resistance upon ROS induction (Tiganis, 2011; Johnson & Olefsky, 2013) by specifying that enhanced 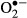 concentrations are largely responsible for repression of IGF-1 signalling and that the 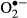-mediated IGF-1 resistance is mainly due to aberrant PTP1B and PTEN translocation and activation. The precise (Vazquez *et al*, 2006; Rahdar *et al*, 2009) mechanism though of PTP1B and PTEN translocation in response elevated 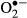 concentrations from the cytoplasm to the plasma membrane needs to be further elucidated.

As to the question how increased 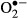 concentrations in the mitochondria transfer the signal to the cytoplasm, it is possible that enhanced mitochondrial 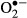 concentrations lead to modifications of kinases, which shuttle between the mitochondria and the plasma membrane exerting phosphorylation and activation of PTP1B and PTEN—the nature of these anticipated kinases remains, however, elusive. Alternatively, longer-lived lipid peroxidation intermediates may transmit the 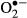-induced signals from the mitochondria to cytosolic and/or membrane-bound PTP1B/PTEN. Notably, 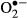 is able to initiate lipid peroxidation by itself, or after reacting with nitric oxide (NO) to form peroxynitrite (ONOO^−^) (Buetler *et al*, 2004; Bashan *et al*, 2009). A recent report suggests that 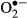 can escape mitochondria and enter the cytosol through mitochondrial voltage-dependent anion channel (Lustgarten *et al*, 2012).

Regarding the question why evolution has promoted the development of an 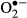-dependent partial IGF-1 resistance, it is important to consider that increased 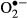
concentrations are extremely noxious to cells. 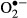-mediated repression of IGF-1 signalling through PTP1B and PTEN activation might have salutary effects on cells with perturbed mitochondria as is the case in type 2 diabetes mellitus, ageing, impaired wound healing, and neurodegenerative disease. In this context, it can act as an oxidant defence mechanism that protects cells by attenuating further ROS generation from increased metabolic or anabolic activity. Also, it can help to promote clearance of damaged cells by apoptosis or by other mechanisms. In fact, lower levels of phosphorylated AKT enhance apoptosis (Franke *et al*, 1997). Thus, 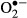-dependent PTP1B and PTEN activation with the induction of IGF-1 resistance may serve as a reliable guardian to protect cells and tissues from 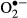-driven oxidative stress and its deleterious consequences.

In summary, we uncovered a mechanism by which enhanced 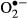 released from dysfunctional mitochondria causes IGF-1 resistance, thereby repressing IGF-1 signalling in mammalian cells. Our studies provide insight into a previously undescribed 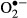-dependent activation of PTP1B and PTEN with subsequent repression of IGF-1 signalling. Targeting these key players may hold promise for the development of novel therapies for age-related pathologies with aberrant IGF-1 signalling such as type 2 diabetes, skin atrophy, osteoporosis, impaired wound healing, and neurodegenerative disease. Unquestionably, future studies should address detailed molecular interactions and identify additional intermediate signalling molecules engaged in 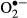-dependent repression of IGF-1 signalling. In addition, it will be exciting to further explore whether—in addition to the IGF-1 dampening—enhanced 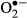 concentrations may affect other receptor tyrosine kinases involved in tissue homeostasis.

## Materials and Methods

### Animal experiments

An inducible connective tissue-specific Sod2-deficient mouse model was used for *in vivo* experiments. This mouse line (Col(I)α2-CreERT^+^;Sod2^f/f^) was generated by crossing Col(I)α2-CreERT transgenic mice (Zheng *et al*, 2002) with Sod2 floxed mice (Strassburger *et al*, 2005; Treiber *et al*, 2011). The generation of Sod2 and PTEN double-mutant mice (Col(I)α2-CreERT^+^;Sod2^f/f^;PTEN^f/+^) was performed by crossing Sod2-deficient mice (Col(I)α2-CreERT^+^;Sod2^f/f^) with PTEN flox mice (PTEN^f/f^) (Groszer *et al*, 2001). All these mice were backcrossed to C57BL/6J for at least 10 generations and were maintained in the Animal facility of University of Ulm with 12 h light–dark cycle and SPF condition. All the animal experiments were approved by the animal ethical committee (Regierungspräsidium Tübingen, Germany). The genotyping of the mice was performed using standard PCR techniques. The sequences of the primers used in PCR-based genotyping are summarized in Supplementary Table S1. In brief, DNA from the tail tip of an individual mouse was purified using a commercial kit (Easy DNA kit, Invitrogen). The purified DNA was later dissolved in TE and used for PCR amplification. The PCR products were run in QIAxcel Advance system (Qiagen) using the program AM320 and then documented digitally. To initiate the activation of CreERT and deletion of Sod2 and PTEN, newborn mice (10 days old) were orally administered with 50 μl of 1 mg/ml of 4-OH tamoxifen (Sigma) suspended in 0.5% methylcellulose and 0.1% Tween-80 (Sigma) for five alternate days. When the mice (both male and female) were 21–25 days old, the mice were subcutaneously implanted with single 60-day release 4-OH tamoxifen pellets (Innovative Research of America). At the age of 65–70 days, mice of both sexes were intraperitoneally (i.p.) injected either with 100 μl of 1 mg/ml recombinant IGF-1 in normal saline or with equal volume of normal saline. Mice were sacrificed 15 or 60 min post-injection, and skin tissues were collected and processed for paraffin embedding and protein lysate preparation.

### Isolation and culture of murine dermal fibroblasts

Murine dermal fibroblasts (MDFs) were isolated from ear skin of young mice and cultured as previously described (Treiber *et al*, 2011).

### Silencing by shRNA

Silencing of *Sod2, PTEN,* and *PTP1B* was performed using lentivirus vector-based shRNA clones from the RNAi consortium (TRC, Broad Institute) (details in Supplementary Methods).

### Serum starvation and IGF-1 stimulation

Murine dermal fibroblasts were washed with PBS and serum-starved for 14 h before stimulation. Stimulation with recombinant mouse IGF-1 (ProSpec) was performed at 100 ng/ml concentration for indicated time points.

### Membrane, cytosolic, and nuclear fractionation

Murine dermal fibroblasts were harvested, and then the membrane and cytosolic extracts were prepared using the Mem-PER extraction reagents according to the manufacturer's instructions (Thermo Scientific). Thereafter, 30 μg of cytosolic lysate or membrane extracts was subjected to Western blot analyses.

### Activity assay of phosphatases

Phosphatases activity assays were performed in immunoprecipitated protein samples (details in Supplementary Methods).

### Immunofluorescence staining and analyses

Immunofluorescence staining was performed as previously described (Treiber *et al*, 2011).

### Quantitative PCR

Quantitative PCR was performed as previously described (Treiber *et al*, 2011) with slight modification (details in Supplementary Methods).

### Cloning and overexpression

Open reading frame (ORF) of mouse *Sod2* was PCR-amplified from its cDNA IMAGE clones (BC010548), and cloned in pcDNA3.1 (Invitrogen) (details in Supplementary Methods).

### Cytotoxicity assay

The viability of murine dermal fibroblasts was measured 3, 6, 12, and 24 h after incubation with rotenone, H_2_O_2_, PTP1B inhibitor, and VO-OHpic using 3-(4,5-Dimethylthiazol-2-yl)-2,5-diphenyltetrazolium bromide (MTT) (Sigma) (details in Supplementary Methods).

### Statistical calculations

All data were calculated using Graphpad Prism (GraphPad Software) or Sigmaplot (Systat Software Inc) and presented as mean ± standard error of mean (SEM). Statistical significance (*P*-value) was calculated using either one-way ANOVA, followed by Bonferroni correction for comparing the difference between more than two samples or by unpaired two-tailed Student's *t*-test for comparing the difference between two samples. For two-tailed *t*-test, the exact *P*-values and for ANOVA, in multiple comparison test (Bonferroni), either the exact *P*-value (when *P* > 0.001, but < 0.05) or the highest *P*-value (*P* < 0.001) were presented. The exact test which was used and the exact *P*-value of comparison are presented in the respective figure legends and figures, respectively.

**The paper explained**

**Problem**

The evolutionarily conserved IGF-1 signalling pathway is associated with longevity, metabolism, tissue homeostasis, and cancer progression. Its action is tightly regulated in multiple steps of activation and inhibition and, when dysregulated, results in organ atrophy with enhanced ageing or cancer progression. During ageing, reduced growth and regeneration occur, which lead to organ and skin atrophy characterized by wrinkle formation, reduced tensile strength of the skin, and impaired wound healing. A reduced function of IGF-1 has long been postulated to inhibit cell proliferation and synthesis of extracellular matrix proteins like collagens in ageing organs; however, the exact mechanisms of IGF-1 suppression are still poorly understood. Of note, the gradual increase in oxidative stress is now established during the ageing process and several pathologies associated with ageing and oxidative stress in elderly individuals. Here, we addressed the impact of oxidative stress on IGF-1 function, its signalling, and on cellular key features of skin ageing. The detailed understanding of the IGF-1 action during oxidative stress in skin ageing and other oxidative stress-related pathologies is of high clinical importance as it holds promise for the development of preventive and/or therapeutic strategies aiming at the delay and reversal of declining skin function.

**Results**

Of note, distinct entities of reactive oxygen species modulate IGF-1 function. Hydrogen peroxide (H_2_O_2_) activates IGF-1 signalling by inhibiting distinct phosphatases involved in IGF-1 suppression. By contrast, accumulation of mitochondrial 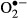 as occurs in ageing skin inactivates IGF-1 signalling. Increasing mitochondrial 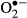 either by chemical means (rotenone treatment) or by genetic silencing of Sod2, the enzyme which normally detoxifies 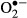
- in murine dermal fibroblasts, leads to the activation and membrane translocation of the two phosphatases PTP1B and PTEN. PTP1B activation and membrane translocation, in fact, lead to its close association with the membrane-anchored IGF-1 receptor; this in turn promotes the dephosphorylation (inactivation) of the IGF-1 receptor (IGF-1R β subunit) with an almost complete abrogation of IGF-1 signalling and subsequent suppressed growth of skin fibroblasts. In addition, PTEN dephosphorylates PIP3 to PIP2 at a later step of IGF-1 signalling, leading to inhibition of AKT phosphorylation (activation), a key step essential for cell growth. Attenuation of 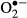-mediated inhibition of IGF-1 signalling and IGF-1 resistance by overexpression of Sod2 further supports the causal contribution of increased 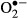 concentrations to IGF-1 resistance. Inhibition of PTP1B and PTEN by specific chemical inhibitors as well as silencing by shRNA *in vitro* significantly rescues IGF-1 resistance even in the presence of higher mitochondrial 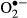. Moreover, deletion of PTEN and inhibition of PTP1B in Sod2-deficient mice also attenuate IGF-1 resistance *in vivo*. Finally, deletion of PTEN rescues the skin phenotype of Sod2-deficient mice.

**Impact**

Our novel finding is that different entities of reactive oxygen species, such as H_2_O_2_ and 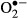, exert opposite effects on the IGF-1 signalling pathway. Our studies provide insight into a previously undescribed 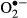-dependent activation of PTP1B and PTEN with subsequent repression of IGF-1 signalling. Targeting these key players may hold substantial promise for the development of novel therapies for age-related pathologies with aberrant IGF-1 signalling such as type 2 diabetes, skin atrophy, osteoporosis, impaired wound healing, neurodegeneration, and cancer.

## Supporting Information

Supplementary Information

Review Process File
