# Supplementary material for: Superoxide anion radicals induce IGF-1 resistance through concomitant activation of PTP1B and PTEN
Source: EMBO Mol Med. 2014 Dec 17;7(1):59–77. doi: 10.15252/emmm.201404082 (PMC4309668; doi:10.15252/emmm.201404082)

Supplementary Information

Table of Contents

Supplementary Methods ………………………….... 2

Supplementary Figure Legends ………………… 6

Supplementary Table ……………………….......... 10

Supplementary Reference ……………………….. 11

Supplementary Figures …..……………………….. 12

Supplementary Methods

Reagents

The monoclonal antibody against ribosomal protein S6 and polyclonal antibodies against insulin-like growth factor-1 receptor β (IGF-1Rβ), AKT, PTEN, Cyclin D1, eIF4G, 4EBP1, Caveolin, HSP90, Sod2 and phosphorylated AKT (S473), and IGF-1Rβ were obtained from Cell Signaling Technology. The polyclonal antibody against PTP1B was from R&D System. Monoclonal antibodies against PP2A were from Cell Signaling (for Western blot) and Millipore (Phosphatase assay). The HRP conjugated actin antibody was obtained from Santa Cruz Biotechnology. Polyclonal antibodies against phospho-tyrosine, phospho-threonine and monoclonal PTEN sepharose bead conjugate were purchased from Cell Signaling Technology. Phospho-serine antibodies were purchased from Millipore. HRP labelled secondary antibodies were from Jackson Immuno Research and Alexa Flour antibodies were obtained from Life Technologies. Protein A/G agarose or magnetic beads were from Thermo Fisher Scientific. Rotenone and other common chemicals were purchased from Sigma. Recombinant mouse IGF-1 was from ProSpec and recombinant human IGF-1 (Increlex) were obtained from Ipsen Pharma. The PTEN inhibitor VO-OHpic was procured from Sigma and the PTP1B inhibitor 3-(3,5-Dibromo-4-hydroxy-benzoyl)-2-ethyl-benzofuran-6-sulfonicacid-(4-(thiazol-2-ylsulfamyl)-phenyl)-amide was purchased from Calbiochem. Restriction endonucleases, antartic phosphatase, T4 DNA ligase and NEB 10-beta electrocompetent cells were purchased from New England Biolabs. Gel extraction kit, nucleotide removal kits, and plasmid isolation kit were purchased from Qiagen.

Silencing by shRNA

The specific shRNA molecules (Supplementary Table 1) were cloned between AgeI and EcoRI sites of a pLKO.1-puro vector. The pLKO.1-puro containing target specific shRNA molecule together with packaging (pMDLg/pRRE, plasmid ID 12260, pRSV-Rev, plasmid ID 12253, Addgene) and the envelop (pMD2.G, plasmid ID 12259, Addgene) plasmids was co-transfected into 293T

cells to produce virus particles as per standard protocol recommended by TRC. Murine dermal fibroblasts ($1x10^6$) were transduced with lentivirus particles at MOI 500µl in presence of polybrene (Hexadimethrine bromide, 1mg/ml culture medium). Twenty-four hours after viral infection, the media was changed and puromycin selection (2µg/ml) was started and continued until resistant colonies appeared in the culture disks. The colonies were then picked and plated with maintenance media (DMEM, with 10% FCS and penicillin-streptomycin) containing puromycin (1µg/ml puromycin). Knockdown efficiency of shRNA clones was analysed by immunoblotting technique.

Immunoprecipitation

MDFs ($1x10^7$) were lysed with 500 µl IP lysis buffer (25mM Tris-HCl pH 7.4, 150mM NaCl, 1mM EDTA, 1% NP-40 and 5% glycerol) containing proteases and phosphatases inhibitors cocktail (Thermo Scientific). The protein concentrations were determined by Bradford assay (Bio-Rad). Primary antibodies (5 µg) (IgG control or IGF-1Rβ, PTEN Sepharose Bead Conjugate, PTP1B, PP2A) were added to 500 µg of protein (precleared with A/G sepharose or magnetic beads) and incubated overnight at 4°C on a rotating platform. Thereafter, except PTEN samples, 20 µl magnetic beads protein A/G (Thermo Scientific) were added to all samples and further incubated for 2h at 4°C on a rotating platform to capture antibody bound protein complexes. PTEN sepharose bead conjugates were directly collected by centrifugation at 1000 rpm for 1 min. The immunoprecipitates bound to magnetic beads were then collected on a magnetic holder. Subsequently, magnetic or sepharose beads were washed three times with IP lysis buffer (Thermo Scientific) and proteins were eluted by boiling in Laemmli buffer containing DTT, and resolved by SDS-PAGE followed by immunoblotting with specific antibodies as previously described (Treiber et al, 2011). The blotted membranes were developed by incubating with LumiGLO (Cell Signaling) using Fusion FX7 Geldoc system (Vilber Lourmat). Densitometric analyses were performed using Fusion Capt software (Vilber Lourmat).

Activity assay of phosphatases

Phosphatases activity assays were performed in immunoprecipitated protein samples. The immunoprecipitation of PTEN, PTP1B and PP2A was performed with specific antibodies as discussed earlier. Immunoprecipitated IGF1Rβ was used to determine the phosphatase activity of IGF1Rβ bound PTP1B. Immunoprecipitated proteins were resuspended in enzyme reaction buffer [1M Tris HCl and 10 mM DTT, pH 7.4-8 in case of PTEN and pNPP Ser/Thr assay buffer (millipore) for PTP1B and PP2A activity assay], and incubated with specific substrates [50 µM PIP3 for PTEN (Echelon), 500 µM Tyrosine phosphopeptide (RRLIEDAEpYAARG) (Calbiochem) for PTP1B) and 500 µM threonine phosphopeptide (KRpTIRR) (Millipore) for PP2A] for 30 min at 37°C. Thereafter, phosphatase reaction was stopped by adding 100 µl malachite green reagent (Millipore). Free phosphate levels were then measured in a mutimode plate reader (Mithras LB940, Berthold technologies) at 620 nm. Absorbance was converted into nmol phosphate per mg protein using a phosphate standard curve.

Quantitative PCR

In brief, total RNA was isolated from murine dermal fibroblasts using the RNeasy kit (Qiagen) as described by the manufacturer. Two µg of RNA per sample was reverse transcribed using illustra Ready-To-Go RT-PCR Beads (GE Healthcare). The quality of total RNA was verified either by Agilent 2100 Bioanalyser (Agilent) or QIAxcel Advance system (Qiagen). The cDNA was amplified in 7300 real time PCR system (Applied Biosystem, Life Technologies) using Power SYBR green mastermix (Applied Biosystems, Life Technologies). Sequences for different primer pairs used for the experiments are listed in the Table 1. Data from real time PCR were analyzed using DataAssist 3.0 Real-Time PCR Analysis Software (Applied Biosystems, Life Technologies).

Superoxide anion induction and quantification

Rotenone, an inhibitor of mitochondrial respiratory chain complex I, was used at different concentrations (1-1000µM) to generate enhanced concentration of

$O_2^{•-}$ in MDFs. $O_2^{•-}$ concentrations were measured in MDFs using $O_2^{•-}$ specific Mitosox staining as previously described (Treiber et al, 2011).

Cytotoxicity assay

The 3-(4,5-Dimethylthiazol-2-yl)-2,5-diphenyltetrazolium bromide (MTT) (Sigma) was used for the quantitation of living metabolically active cells. Mitochondrial dehydrogenases reduce MTT to purple formazan dye (Green et al, 1984). Cytotoxicity was calculated as the percentage of formazan formation in cells treated with different agents compared with mock-treated cells. Conditions were established that > 90% of murine dermal fibroblasts are proven vital.

Cloning and overexpression

The Sod2 ORF was cloned into NheI and BamHI sites of pcDNA3.1+ (Life Technologies). The plasmid sequence was verified through a commercial service (Sequiserve, Germany). The detailed sequences of the primers used for cloning is listed in Supplementary Table 1. MDFs were transiently transfected with either pcDNA3.1+ (Vector control) or with pcDNA3.1-Sod2 (Sod2 OE) using attractene transfection reagent (Qiagen) as per instruction manual.

Murine keratinocyte culture

Primary murine keratinocyte from C57BL/6 mice were purchased from cellntech and maintained in defined epidermal keratinocyte media (Cellntech) under 5% CO_2 and 37°C.

Supplementary Figure Legends

Supplementary Figure 1

Rotenone inhibits the mitochondrial respiratory chain complex I with enhanced generation of superoxide anions in mouse dermal fibroblasts (MDFs). **(A)** Schematic diagram showing the mode of action of rotenone for superoxide anion generation in the mitochondrial respiratory chain. Rotenone-dependent inhibition of complex I of the respiratory chain results in the leakage of electrons which are transferred to molecular oxygen (O_2) to form superoxide anion radicals ($O_2^{\bullet-}$). **(B)** Mitochondrial $O_2^{\bullet-}$ concentrations were measured by the superoxide anion specific MitoSOX dye in living cells. The graph depicts emission fluorescence intensities measured at ~580 nm wavelength range (excitation 396 nm) in MDFs treated with rotenone at the indicated concentrations (10 to 1000 µM) for 30 min. Values are presented as mean ± SEM of percent difference of fluorescence with the non-treated control MDFs set as zero. Comparison was made with one-way ANOVA followed by Bonferroni correction (n=3). Comparison was made for each group with MDFs in the absence of rotenone. **(C)** Cell viability assay was performed with MTT. Values are mean ± SEM of percent viability and comparison was made with two-tailed t-test (n=6). **(D)** Mitochondrial $O_2^{\bullet-}$ levels were measured by the $O_2^{\bullet-}$-specific MitoSOX dye in MDFs transduced with lentivirus particles containing Sod2 shRNA or non-targeting shRNA, comparison was made with, two-tailed t-test. **(E)** in MDFs with transiently transfected control vector or *Sod2* expression vector, as described in Materials and Methods. Values are mean ± SEM of MitoSOX fluorescence in percentage, comparison was made with one-way ANOVA followed by Bonferroni correction (n=6).

Supplementary Figure 2

Enhanced superoxide anion radical concentrations dampen IGF-1 mediated proliferation. To dissect whether rotenone mediated enhanced $O_2^{\bullet-}$ concentrations and H_2O_2 affect IGF-1 dependent proliferation, representative immunostainings of Ki-67 (green) and BrdU (red) in monolayer cultures of non-treated MDFs (control) MDFs treated with 100ng/ml IGF-1 in the presence or

absence of 100μM rotenone for 12h are depicted. Nuclei were stained with DAPI (blue). Scale bars, 20μm.

Supplementary Figure 3

Superoxide anions inhibit IGF-1 signalling and induce partial resistance in murine keratinocytes. Murine keratinocytes stimulated with 100ng/ml IGF-1 or unstimulated keratinocytes in the presence or absence of 350μM and 500μM rotenone for 3h were assessed for expression of the indicated IGF-1 downstream effector proteins in their basal (IGF-1Rβ, AKT, S6) and phosporylated (activated) states (pAKT, pS6) by Western blotting. Representative Western blots out of 2 independent experiments are shown here.

Supplementary Figure 4

(A) Genotyping PCR analyses of the tamoxifen inducible fibroblast specific *Sod2* deficient mice. Representative PCR analyses showing deletion of the *Sod2* gene after tamoxifen administration. Floxed band (f), deleted band (d). The PCR amplified products for both Col(I)α2-CreERT and Sod2 were run in Qiaxcel capillary electrophoresis system (Qiagen) and analysed. The genotyping PCR of Col(I)α2-CreERT and Sod2 were performed two times, one before 4-hydroxy tamoxifen treatment and another after completion of tamoxifen treatment (details in Materials and Methods). M, DNA ladder; lane 1-6, PCR results from genomic DNA of different mice. **(B)** Immunoblotting showing the expression of Sod2 in the skin lysates from fibroblast specific *Sod2* wildtype (control) and fibroblast specific Sod2 deleted mutant mice. Actin was used as loading control. **(C)** Skin lysates from control mice (with normal $O_2^{\bullet -}$ concentrations) and mutant mice (with enhanced $O_2^{\bullet -}$ concentrations in fibroblasts) were prepared 60 min after i.p. injection of 1mg/ml IGF-1 or saline and the expression of pAKT (S473) and AKT was analysed by Western blotting and equilibrated to actin expression levels. A representative Western blot out of 3 independent experiments is shown here. Graph (right panel) depicts densitometric analyses of pAKT/AKT ratio after correction with actin (loading control) for each of the four groups (n=3).

Supplementary Figure 5

Quantitative analyses of PTP1B and PTEN membrane translocation in murine dermal fibroblasts. **(A)** Densitometric analyses of PTEN in cytocsolic and membrane fraction of control, H_2O_2 and rotenone treated MDFs. Values are mean (relative density) ± SEM in percent and comparison was made with two-tailed t-test (n=3), comparison was indicated with line and P value. **(B)** Densitometric analyses of PTP1B in cytosolic and membrane fraction of control, H_2O_2 and rotenone treated MDFs. Values are mean (relative density) ± SEM in percent and comparison was made with two-tailed t-test (n=3), comparison was indicated with line and P value.

Supplementary Figure 6

Rotenone induced posttranslational modifications in PTEN. Overnight serum starved MDFs were treated with rotenone (500µM) or vehicle for 3 hrs. MDFs were then lysed to purify cytosolic and membrane fraction. Equal amount of proteins (50µg) from either cytosolic or membrane fraction of control and rotenone treated MDFs were used for immunoblotting to detect PTEN, caveolin 1 (membrane specific marker), HSP90 (cytoplasm specific marker). Actin was used as a loading control. Immunoprecipitation with PTEN antibody was performed using 400µg of protein from either the cytosolic or membrane fraction. The elution of the immunoprecipitated samples was used for immunoblotting with PTEN, phospho Serine (pSer), phospho Threonine (pThr) and phospho Tyrosine (pTyr) antibodies. The shown experiment has independently repeated two times.

Supplementary Figure 7

Pharmacological inhibition of PTP1B promotes IGF-1R activation in superoxide anion exposed MDFs. MDFs were treated as indicated, cell lysates were immunoprecipitated (IP) with anti-IGF-1Rβ antibodies followed by immunoblotting with anti-pIGF-1Rβ (Tyr 1135,1136) antibodies. Graphs represents densitometric analyses of ratio of pIGF-1Rβ and IGF-1Rβ. Comparison was performed using two-tailed t-test (n=3). Comparison was

indicated with line and P value. The representative experiment shown has independently been repeated three times.

Supplementary Figure 8

(A) Genotyping PCR analyses of the tamoxifen inducible fibroblast specific $Sod2^{-/-}$ and $Sod2^{-/-}$;$PTEN^{+/-}$ deficient mice. Representative PCR analyses depict absence or presence of Col(I)α2-CreERT, double flox band (f) and deleted flox band (d) of Sod2 and flox (f) as well as wildtype (+) band of PTEN. The PCR amplified products for were run in Qiaxcel capillary electrophoresis system (Qiagen) and analysed. M, DNA ladder; lane 1-6, PCR results from genomic DNA of 6 different mice. **(B)** Immunoblotting showing the expression of Sod2 and PTEN in the skin lysates from fibroblast specific *Sod2* wildtype (control) and fibroblast specific Sod2 deleted (mutant) and double mutant (Sod2 homozygous and PTEN heterozygous deleted) mice. Actin was used as loading control.

Supplementary Table 1

Gene	Oligo Sequences	Application
Col(I)α2-CreERT	FP:GACATGTTCAGGGATCGCCAGGCG<br>RP:GACGGAAATCCATCGCTCGACCAG	Genotyping
Sod2	FP:GAGGGGCATCTAGTGGAGAA<br>RP:GAAAGTCACCTCCACACACAGA	Genotyping
PTEN	FP: CAAGCACTCTGCGAACTGAG<br>RP: AAGTTTTTGAAGGCAAGATGC	Genotyping
Col(I)α1	FP:TGGCCAAGAAGACATCCCTGAAGTC<br>RP:GGCAGATACAGATCAAGCATACCTCGG	QPCR
Col(I)α2	FP:CTGGTCTTACTGGGAACTTTGCTGC<br>RP:CCAACAGCACCAGGAGGGCC	QPCR
Col(III)α1	FP:CAAACACGCAAGGCAATGAGACTACC<br>RP:AGGGCCAATGTCCACACCAAATTC	QPCR
Beta-Actin	FP:CCTTCTTGGGTATGGAATCCTGTGG<br>RP:CAGCACTGTGTTGGCATAGAGGTCTTTAC	QPCR
Sod2	FP: AAGCTGGCTAGCATGTTGTGTCGGGCGGCG<br>RP: ACTAGTGGATCCTCACTTCTTGCAAGCTGTGTATCTTTCAG	Cloning (ORF amplification)
PTP1B	Forward Oligo:<br>CCGGCACTGAAGTTAGGAGACGGATCTCGAGATCCGTCTCCTAACTTCAGTGTTTTTG<br>Reverse Oligo:<br>AATTCAAAAACACTGAAGTTAGGAGACGGATCTCGAGATCCGTCTCCTAACTTCAGTG	shRNA
PTEN	Forward Oligo:<br>CCGGCGACTTAGACTTGACCTATATCTCGAGATATAGGTCAAGTCTAAGTCGTTTTTG<br>Reverse Oligo:<br>AATTCAAAAACGACTTAGACTTGACCTATATCTCGAGATATAGGTCAAGTCTAAGTCG	shRNA
Sod2	Forward Oligo:<br>CCGGCCCAAACCTATCGTGTCCATTCTCGAGAATGGACACGATAGGTTTGGGTTTTTG<br>Reverse Oligo:<br>AATTCAAAAACCCAAACCTATCGTGTCCATTCTCGAGAATGGACACGATAGGTTTGGG	shRNA

Supplementary Reference

Green LM, Reade JL, Ware CF (1984) Rapid colorimetric assay for cell viability: application to the quantitation of cytotoxic and growth inhibitory lymphokines. *J Immunol Methods* **70:** 257-268

Groszer M, Erickson R, Scripture-Adams DD, Lesche R, Trumpp A, Zack JA, Kornblum HI, Liu X, Wu H (2001) Negative regulation of neural stem/progenitor cell proliferation by the Pten tumor suppressor gene in vivo. Science 294**:** 2186-2189

Strassburger M, Bloch W, Sulyok S, Schuller J, Keist AF, Schmidt A, Wenk J, Peters T, Wlaschek M, Lenart J et al (2005) Heterozygous deficiency of manganese superoxide dismutase results in severe lipid peroxidation and spontaneous apoptosis in murine myocardium in vivo. Free radical biology & medicine 38**:** 1458-1470

Treiber N, Maity P, Singh K, Kohn M, Keist AF, Ferchiu F, Sante L, Frese S, Bloch W, Kreppel F et al (2011) Accelerated aging phenotype in mice with conditional deficiency for mitochondrial superoxide dismutase in the connective tissue. Aging Cell 10**:** 239-254

Zheng B, Zhang Z, Black CM, de Crombrugghe B, Denton CP (2002) Ligand-dependent genetic recombination in fibroblasts: a potentially powerful technique for investigating gene function in fibrosis. The American journal of pathology 160**:** 1609-1617

Supplementary Figure 1

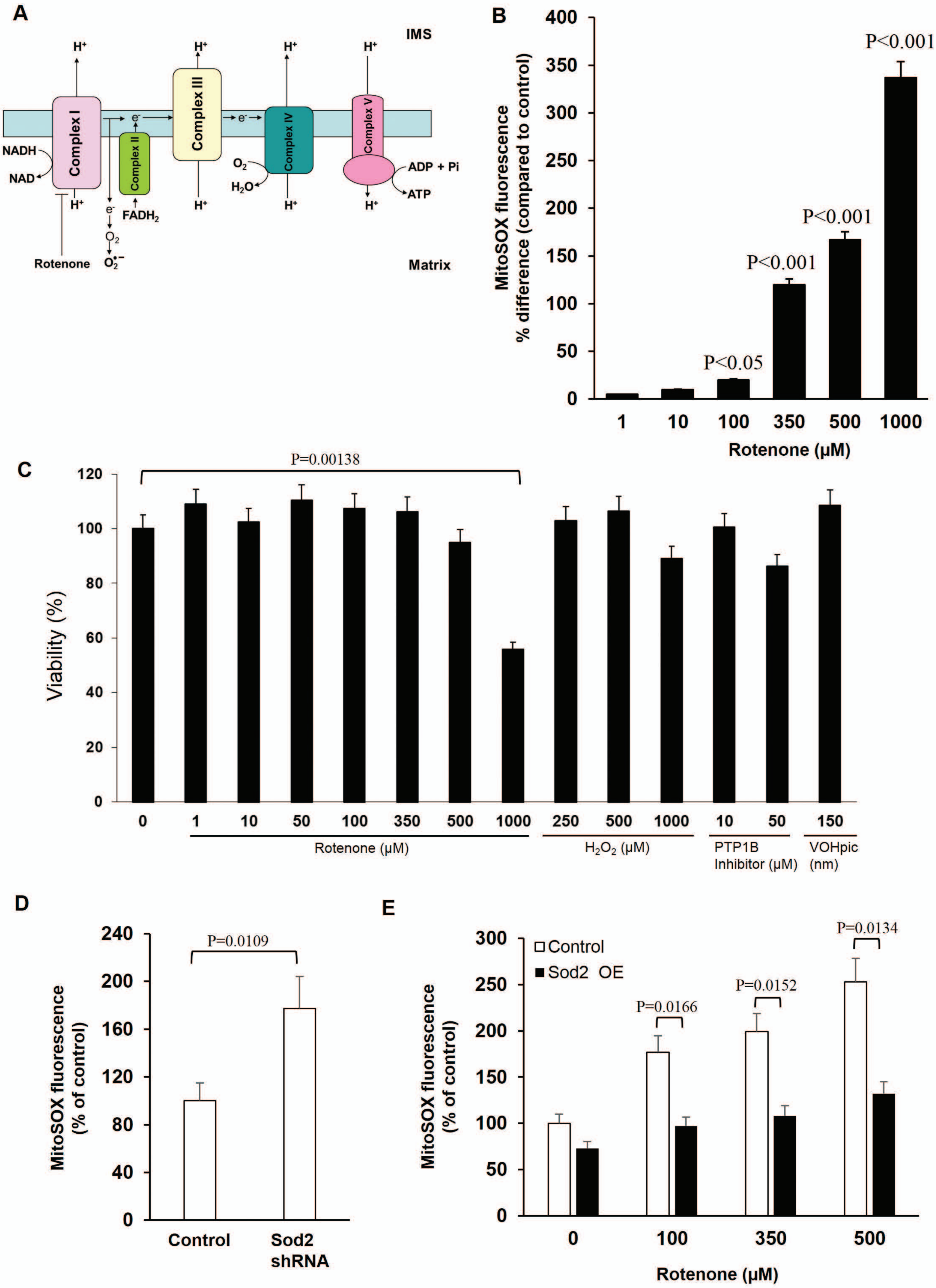

Supplementary Figure 2

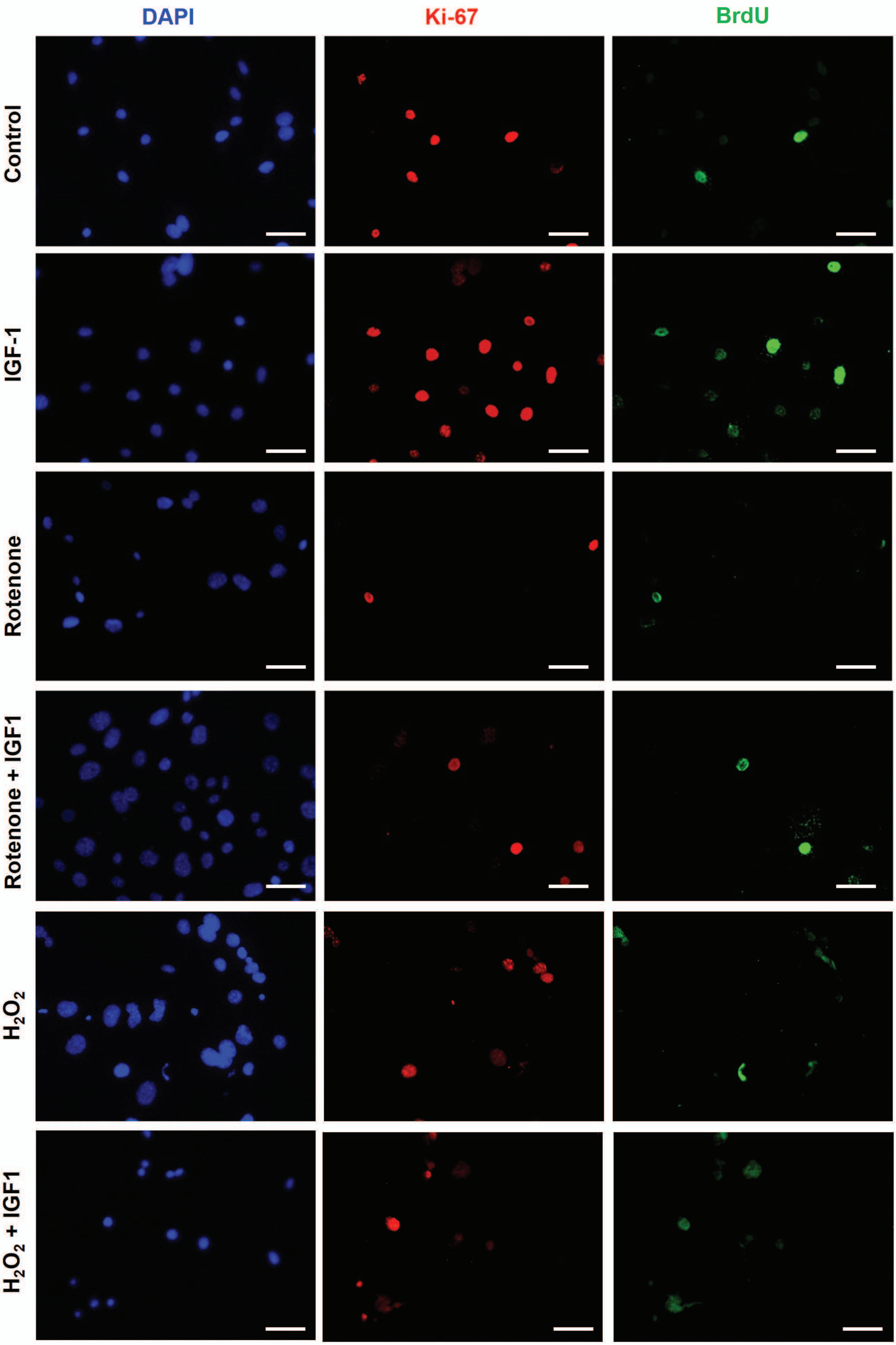

Supplementary Figure 3

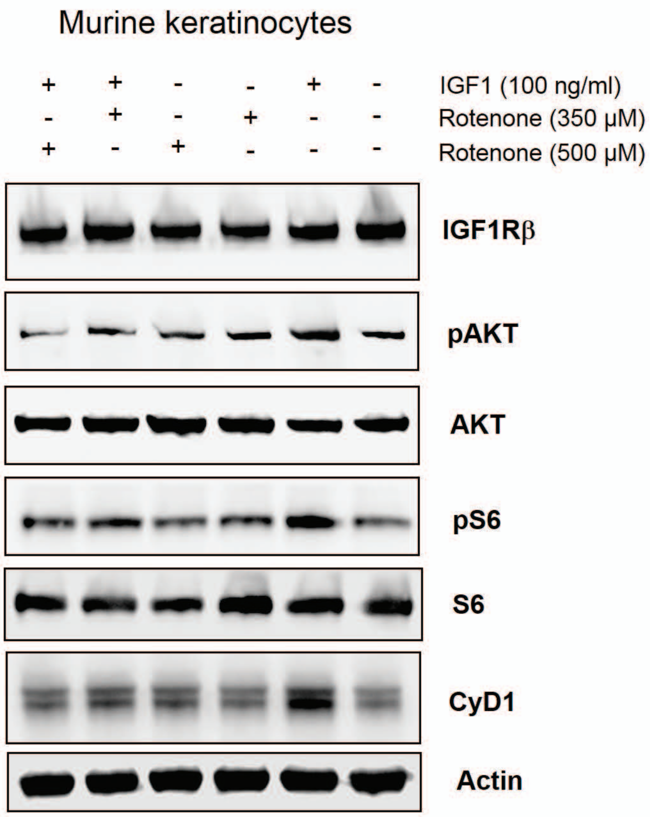

Supplementary Figure 4

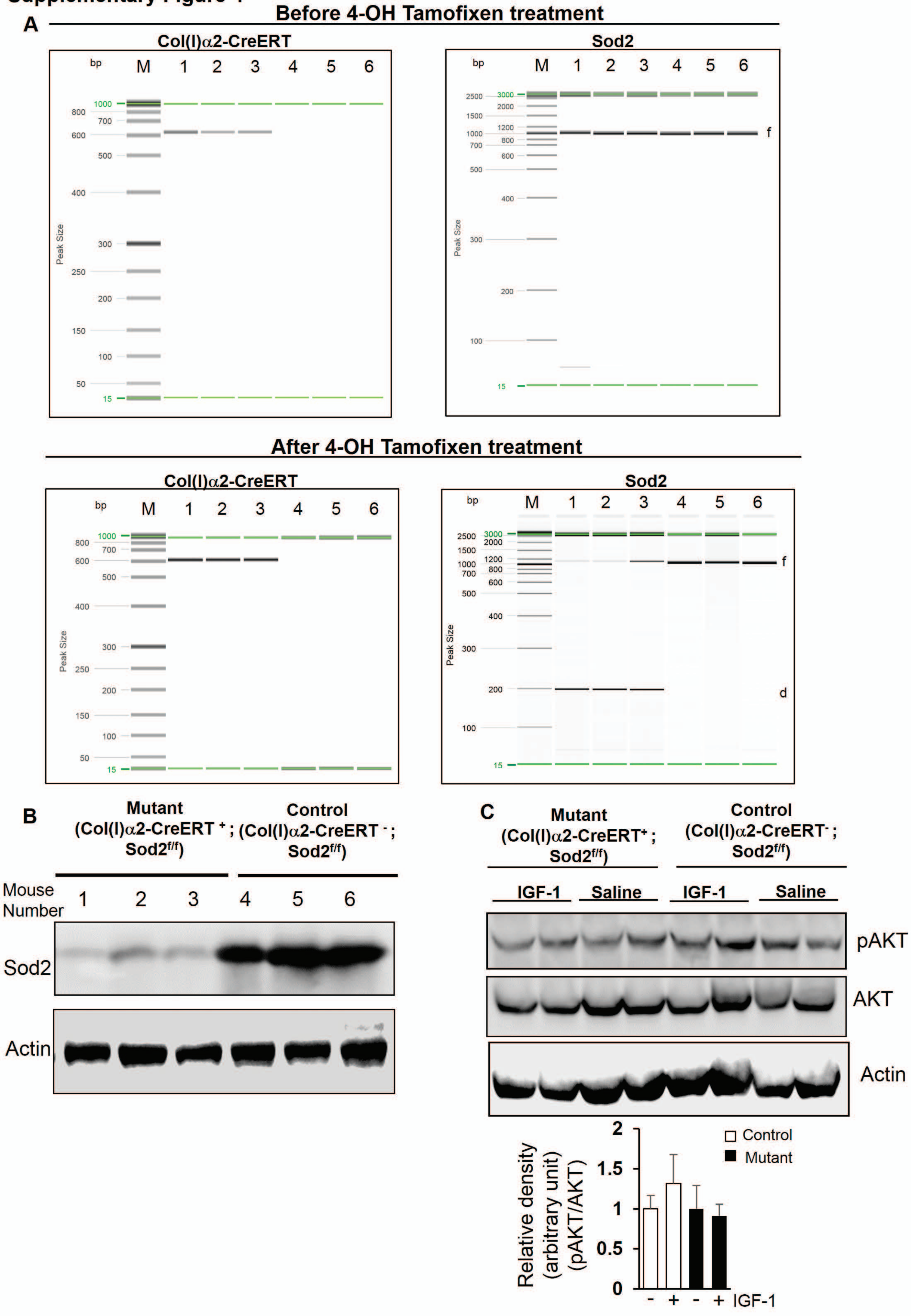

Supplementary Figure 5

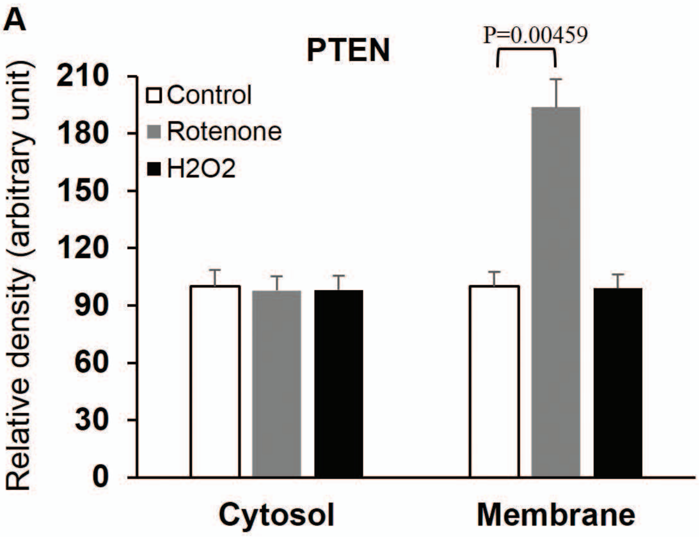

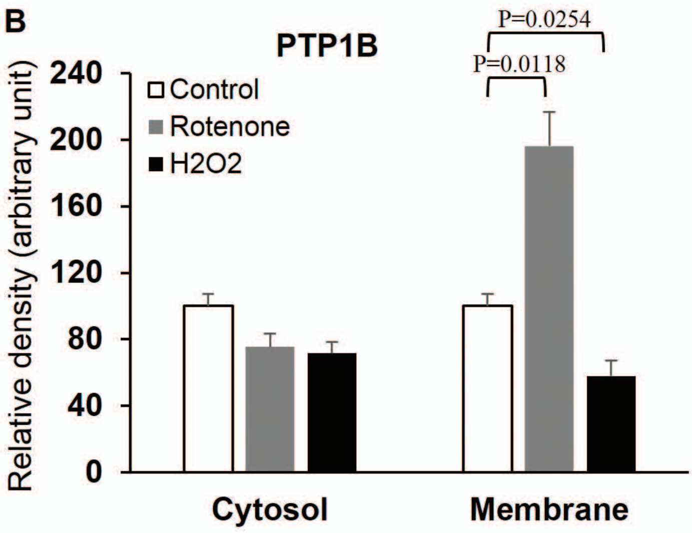

Supplementary Figure 6

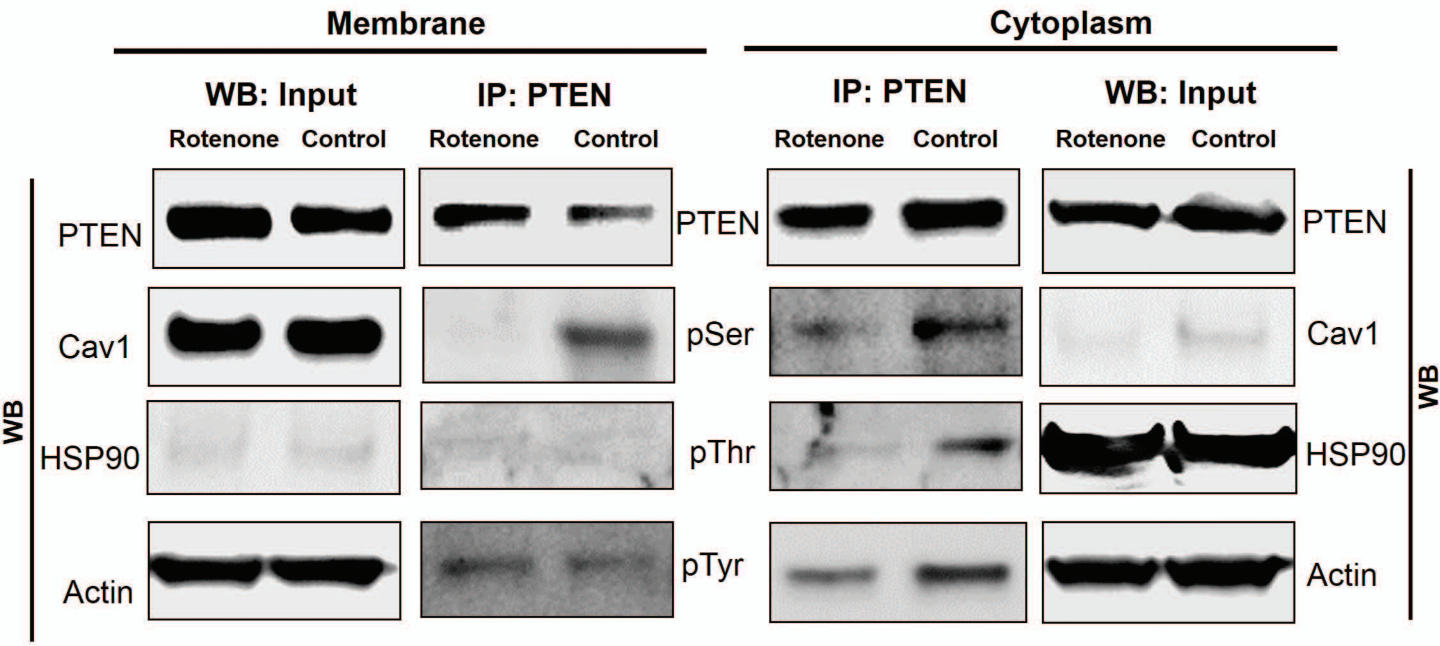

Supplementary Figure 7

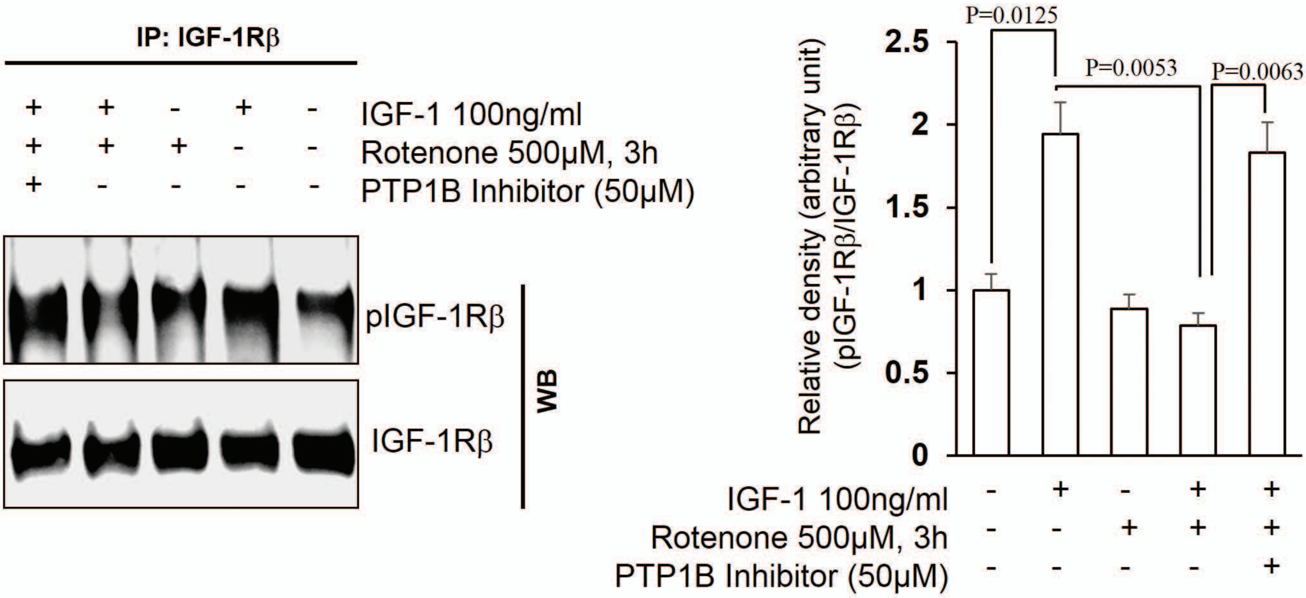

Supplementary Figure 8

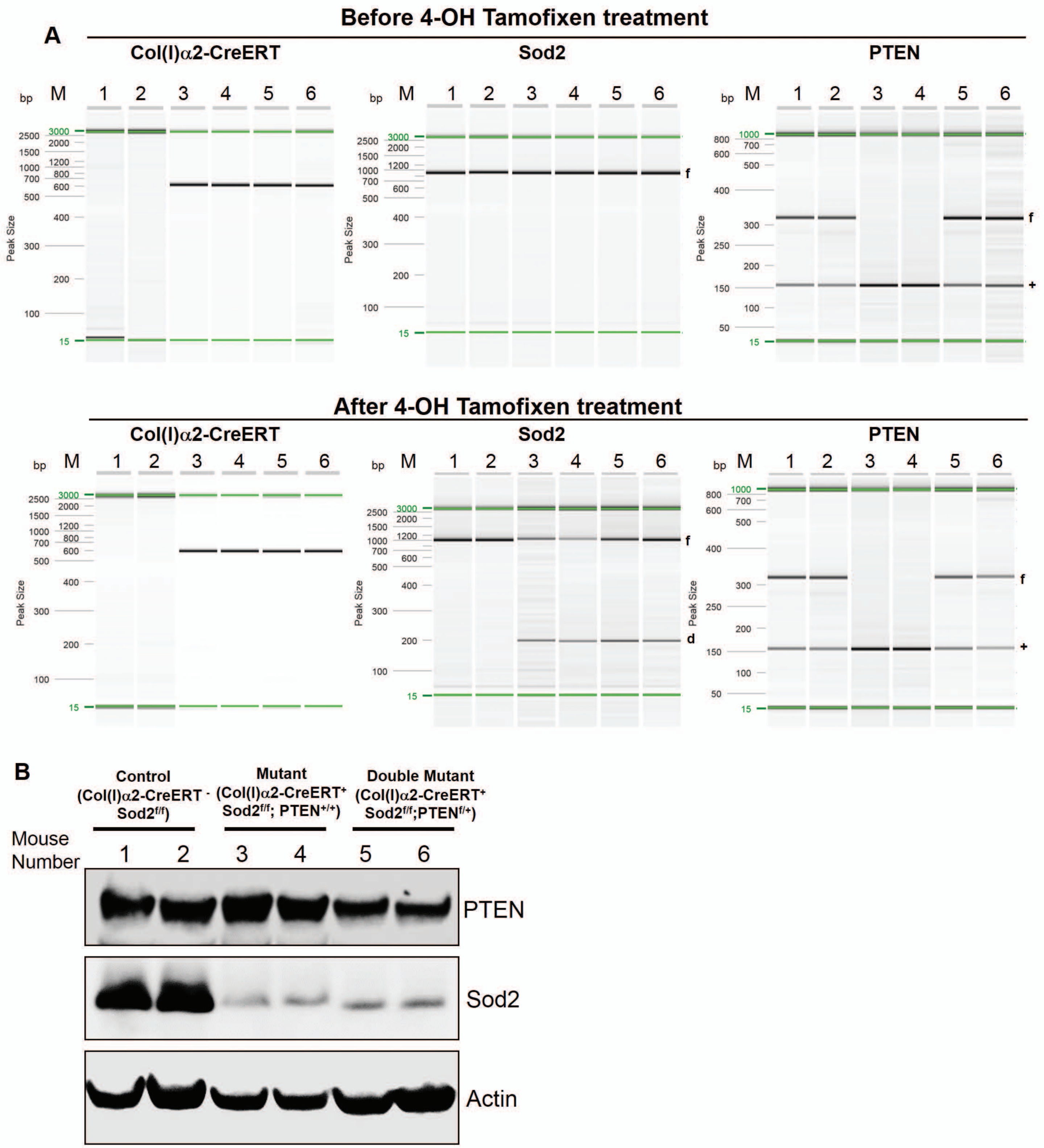

Supplement: Supplementary file 1 [file emmm0007-0059-sd1.pdf]
