# Supplementary material for: Superoxide anion radicals induce IGF-1 resistance through concomitant activation of PTP1B and PTEN
Source: EMBO Mol Med. 2014 Dec 17;7(1):59–77. doi: 10.15252/emmm.201404082 (PMC4309668; doi:10.15252/emmm.201404082)
Supplement: Supplementary file 2 [file emmm0007-0059-sd2.pdf]

Superoxide anion radicals induce IGF-1 resistance through concomitant activation of PTP1B and PTEN

Karmveer Singh, Pallab Maity, Linda Krug, Patrick Meyer, Nicolai Treiber, Tanja Lucas, Abhijit Basu, Stefan Kochanek, Meinhard Wlaschek, Hartmut Geiger and Karin Scharffetter-Kochanek

Corresponding author: Karin Scharffetter-Kochanek, University of Ulm

Review timeline:

Submission date:	17 March 2014
Editorial Decision:	18 April 2014
Revision received:	16 October 2014
Editorial Decision:	30 October 2014
Revision received:	06 November 2014
Editorial Decision:	13 November 2014
Accepted:	13 November 2014

Transaction Report:

(Note: With the exception of the correction of typographical or spelling errors that could be a source of ambiguity, letters and reports are not edited. The original formatting of letters and referee reports may not be reflected in this compilation.)

Editor: Roberto Buccione

1st Editorial Decision 18 April 2014

Thank you for the submission of your manuscript to EMBO Molecular Medicine. We have now heard back from the three Reviewers whom we asked to evaluate your manuscript.
You will see that two Reviewers are more supportive of you work while one is quite negative. All considered, the concerns expressed prevent us from considering publication at this time. I will not dwell into much detail, and just mention a few main points.

Reviewer 1, while globally positive, expresses multiple concerns on the quality and presentation of the data and also points to several instances where clarification is required. In addition s/he would like to know if the IGF-1R activates the MAPK pathway in the dermal fibroblasts. This Reviewer also lists other items that require your attention.

Reviewer 2 is fundamentally negative and challenges the notion that the use of rotenone is an acceptable approach to induce/gauge superoxide production due to the fact that rotenone also has many other effects including on ATP levels, substrate utilization and possibly on other signaling pathways. S/he suggests that exploiting more the Sod2-/- system more would help address this fundamental issue. Finally, this Reviewer also disagrees with the soundness of the model presented.

Reviewer 3 while positively impressed by the work, notes many important issues that need to be addressed to render the manuscript stronger and more compelling and relevant for the community. A relevant one is to verify if homozygote or heterozygote suppression or reversal of PTEN or PT1PB reverses or alleviates the phenotype of homozygote SOD2 deficiency in the conditional mouse model. I agree that this set of experiments is important and, while demanding, would serve to also help address the concerns raised by Reviewer 2 which appear to be well-founded. Finally, I note that Reviewer 3 also points to shortcomings in the quality of the data presented.

Considered all the above, while publication of the paper cannot be considered at this stage, we would be prepared to consider a substantially revised submission, with the understanding that the Reviewers' concerns must be fully addressed, with additional experimental data where appropriate and that acceptance of the manuscript will entail a second round of review.

Since the required revision in this case appears to require a significant amount of time, additional work and experimentation and might be technically challenging, I would understand if you chose to rather seek publication elsewhere at this stage. Should you do so, we would welcome a message to this effect.

I look forward to seeing a revised form of your manuscript as soon as possible.

***** Reviewer's comments *****

Referee #1 (Remarks):

The evolutionary conserved IGF-1 signaling pathway plays an important role in the regulation of longevity, metabolism, tissue homeostasis and cancer. Its regulation depends on the delicate balance between activating kinases and suppressing phosphatases and is still not very well understood. The authors report here that IGF-1 signaling in vitro (Murine dermal fibroblasts (MDFs) and in a murine aging model in vivo is suppressed in response to accumulation of superoxide anions (O2•-) in mitochondria, either by chemical inhibition of complex I or genetic silencing of O2•--dismutating mitochondrial Sod2. The O2•--dependent suppression of IGF-1 signaling resulted in decreased proliferation of murine dermal fibroblasts, influenced translation initiation factors and suppressed the expression of α1(I), α1(III), and α2(I) collagen, hallmarks of skin aging. Enhanced O2•- lead to activation of the phosphatases PTP1B and PTEN which via dephosphorylation of the IGF-1 receptor and phosphatidylinositol 3,4,5-triphosphate dampen IGF-1 signalling. Genetic and pharmacologic inhibition of PTP1B and PTEN abrogated the O2•--induced IGF-1 resistance. The authors identified previously unreported signature events with O2•-, PTP1B and PTEN as promising targets for drug development to prevent IGF-1 resistance related pathologies.

The paper is quite interesting and provides several novel results in mechanisms regulating IGF-I signaling in aging. However, there are some issues that need to be address/clarified to improve the manuscript for publication:

1) Abstract. "The evolutionary conserved IGF-1 signalling pathway is associated with longevity, metabolism, tissue homeostasis and cancer suppression". The first sentence of the Abstract is very misleading. The role of the IGF-I pathway in cancer initiation is quite well established and therefore not associated with cancer suppression. Please rephrase this sentence.
2) Introduction, page 3. There is strong evidence in the literature supporting the notion that IGF-IR-dependent cell proliferation and transformation requires the activation of both the Akt and the MAPK Pathways. This aspect has to be more clearly indicated in the Introduction with additional references.
3) Page 6: "markedly induced pAKT" Please change to induced phosphorylation of Akt...
4) Figure 1: are the experiments shown in Panels A and B performed in SFM or in serum-containing media? Please specify in Figure Legends.
5) The effects on cyclinD1 levels are not great, especially in Panel A. Please add densitometric analysis in Panels A and B for change in Cyclin D1 levels.
6) As correctly shown in Figure 2A, the IGF-IR activates both the MAPK and Akt pathways, which both regulate cyclin D1 levels. Does the IGF-IR activate the MAPK pathway in Murine dermal fibroblasts? If it does, it would be appropriate to show in Fig. 1 and 2 IGF-I-induced ERKs activation with or without Rotenone or H2O2. This is important because the effect of PTP1B on IGF-IR phosphorylation is going to affect not only the Akt pathway but the MAPK pathway as well. If it does not, it would be important to mention it in the Introduction. Please also modify the Synopsis accordingly.

7) Figure 2, Panel C: the differences are not great and the blots are overexposed. Please add densitometric analysis. 4EBP1 blot shows at least two bands: which is the right one?
8) Figure 2, Panel D: is H_2O_2 affecting IGF-I-dependent proliferation?
9) The effect of Rotenone on IGF-IR phosphorylation is only shown in Fig. 4. This is an important piece of evidence and I would suggest showing it already in Fig. 2 B in conjunction with IGF-IR levels.
10) Discussion, Page 13: "Enhanced $O_2^{•-}$ concentrations occur in several physiological and pathological conditions" Please include some examples with references.
11) Discussion, Page 16: One issue that is still somewhat puzzling is "how increased $O_2^{•-}$ concentrations in the mitochondria transfer the signal to the cytoplasm" This issue is touched in the Discussion section but it may deserve a more extended discussion. Is there the possibility that $O_2^{•-}$ may leak from the mitochondria to the cytosol?

Referee #2 (Comments on Novelty/Model System):

as mentioned in my comments, I do not believe the use of rotenone is a good approach. This is fundamental to the manuscript and as such, I cannot be too enthusiastic

Referee #2 (Remarks):

The manuscript by Singh and colleagues explores the role of superoxide anions on IGF-1 signaling. In particular, the authors attempt to distinguish the biological role of superoxide anions versus hydrogen peroxide. While there is a growing literature on how reactive oxygen species (ROS) might function as modulators of signaling pathways, relatively little is known as to how different ROS molecules may function. As such, the general area is of interest. Nonetheless, my major concern is whether the experimental system is as clear and unambiguous as assumed. In large part, the authors use the drug rotenone to induce superoxide anions. This agent is thought to act by inhibiting Complex I of the mitochondria. While it is true that the initial ROS produced is O_2^-, this molecule is rapidly dismutated (with or without SOD) into hydrogen peroxide. Thus, there is plenty of previous literature where rotenone is used by other authors to increase hydrogen peroxide levels in the cell. As such, while the authors compare the effects of exogenous hydrogen peroxide versus rotenone treatment as a system to explore the role of O_2^- versus hydrogen peroxide, I'm not convinced this is accurate or as clearly distinguishable as the authors portray. As such, I'm not convinced the author's conclusions are justifiable.
Along these lines, while the authors attribute the effects of rotenone on superoxide production, it's not clear this is the only thing this mitochondrial agent is doing. What about effects on ATP levels or substrate utilization? Can the authors demonstrate the specificity of their observations? Are other signaling pathways affected by rotenone treatment or is it just the IGF-1 pathway? One way of further solidifying the data would be to exploit the Sod2-/- system more. Can the results of Figure 4D and E be repeated using WT and Sod2 deficient cells? Another way would be to increase the quantitative aspect of the data. In that regard, can the main point of Figure 5 be quantitatively assessed? Given the short half life of superoxide anions and their inability to pass through lipid bilayers, I'm not sure how the model shown in Figure 7 makes sense. Again, this all revolves around my difficulty in accepting the basic premise that the authors have created a pure superoxide anion model by treating cells with rotenone.

Referee #3 (Remarks):

This is an interesting and well written paper from a group with a track record in aging and dermatology. The paper extends previous work on conditional homozygote progeria like syndrome in mice following a collagen Cre mediated deletion. The main premise is the reciprocal effects of $O_2^{•-}$ and H_2O_2, through conversion by SOD2, on the IGF1-PTP1B/PTEN pathway, namely suppression of the pathway by $O_2^{•-}$ and activation by H_2O_2 (through phosphatase inactivation via oxidation of cysteines of the negative regulators PTP1B/PTEN). Both activation and inhibition of the IGF signaling pathway by ROS have been described by others, although there is more focus in

this paper on the O2•- mediated suppression of the IGF signaling pathway via relocation of PTB1B and PTEN.

General comments
The main and new contribution to the field mainly concerns the translocation of PTP1B/PTEN to the cytoplasmic membrane following rotenone treatment to increase O2•- . However, for some reason the authors confine important control data (that is relatively non-quantitative) that relates to the rescue of the phenotype following SOD2 overexpression to supplementary data (suppl Fig 5). This could be better placed in the main part of the paper. Importantly, the group suggest that snRNA mediated suppression of either PTP1B/PTEN reverses the effect of IGF1 signaling suppression caused by upregulation of O2•- . However, the key experiment in my view, and within the capability of the group, is whether homozygote or heterozygote suppression or reversal of Pten or Ptp1b reverses or alleviates the phenotype of homozygote SOD2 deficiency in their conditional mouse model. These data would make a more compelling argument for the in vivo relevance of their data and for publication in EMBO Molecular Medicine. Moreover, in addition, the main data presented do not appear to be all that significant, as most of the effects were of the order of two fold and have been variably analysed statistically with either two way or one way analysis. For this reason also, the in vivo magnitude of the effects over time would be more informative than acute responses to maximal IGF1 stimulation at fixed time points.

Minor comments

1. The mouse model uses a prolonged Cre activation to delete SOD2, and the response to IGF1 injections is only carried out at one time point. The quantification of response is only presented after a short time in Figure 3A and 3B. Consideration should be given to at least another time point and explanation given for poor efficiency and need for continued Cre activation.
2. P4. Sentence beginning; "Also mouse lacking...." Does not make sense.
3. P8 para2 line 3, MDF or MFD?
4. P21 10_days
5. P31Figure 2D legend, "To role..." does not make sense?
6. Fig 2E, over contrast on western blots. Have these been imaged enhanced?

1st Revision - authors' response 16 October 2014

Referee 1

Re:1 Abstract. "The evolutionary conserved IGF-1 signalling pathway is associated with longevity, metabolism, tissue homeostasis and cancer suppression". The first sentence of the Abstract is very misleading. The role of the IGF-I pathway in cancer initiation is quite well established and therefore not associated with cancer suppression. Please rephrase this sentence.

As suggested by Reviewer 1, the word 'suppression' was replaced by the word 'progression' (Page 2, Line 3, Abstract section).

The revised portion(s) is(are) marked with yellow colour.

Revised version of the manuscript, Page 2, line 3 Abstract section:
The evolutionary conserved IGF-1 signalling pathway is associated with longevity, metabolism, tissue homeostasis and cancer progression.

Re:2 Introduction, page 3. There is strong evidence in the literature supporting the notion that IGF-IR-dependent cell proliferation and transformation requires the activation of both the Akt and the MAPK Pathways. This aspect has to be more clearly indicated in the Introduction with additional references.

Following this Reviewer´s suggestion, we have discussed the multifaceted role of MAPK activation by IGF-1 signalling and have added appropriate references in the revised manuscript.

Revised version of the manuscript, Introduction, Page 3, Line 29-33 and Page 4, 1-3, Introduction section:
In addition to the PI3K-AKT axis, IGF-1 signalling was reported to activate the mitogen activated protein kinase (MAPK) pathway thus exerting its prosurvival effect in many though not all cell lines (Parrizas et al, 1997; Peruzzi et al, 1999; Subramaniam et al, 2005). In fact, IGF-1 can also inhibit ERK activation in some cell types, including neurons (Subramaniam et al, 2005). Therefore, the effect of IGF-1 on MAPK and Cyclin D1 expression is not uniform for all cell types and most likely depends on the cell type, the nature, magnitude and duration of the stimulus.

Re:3 Page 6: "markedly induced pAKT" Please change to induced phosphorylation of Akt...

The sentence is changed to 'induced phosphorylation of AKT' in the revised manuscript.

Revised version of the manuscript, Result section, Page 6, Line 20, Result section:
H_2O_2 at moderate and high concentrations ranging from 10 µM to 1000 µM markedly induced phosphorylation of AKT without exerting any effect on total AKT levels (Figure 1A), a finding which is in agreement with published reports (Ushio-Fukai et al, 1999).

Re:4 Figure 1: are the experiments shown in Panels A and B performed in SFM or in serum-containing media? Please specify in Figure Legends.

The experiments shown in Figure 1, Panels A and B are performed in serum free medium. The Figure legend was revised according to this Reviewer´s suggestion.

Revised version of the manuscript, Page 31, Line 8, Figure 1, Legend Section:
(A) To assess differential regulation of downstream key effector proteins of IGF-1 signalling such as activated (phosporylated) AKT (pAKT), total AKT (AKT) and Cyclin D1 levels (CyD1) in MDFs either treated with increasing rotenone concentrations (left panel) or with increasing H_2O_2 concentrations (right panel) for 3 h in serum free DMEM, cell lysates were analysed by Western blotting equilibrated to actin expression levels.

Re:5 The effects on cyclinD1 levels are not great, especially in Panel A. Please add densitometric analysis in Panels A and B for change in Cyclin D1 levels.

Densitometric analyses of Cyclin D1 levels for both panel A and panel B have been added in the revised manuscript (Figure 1A, B).

Revised version of the manuscript, Page 31, Line No. 11-14 and 17-19, Figure Legend Section, Figure 1:
(A) …. The graphs represent the densitometric analyses of Cyclin D1 (CyD1) expression in the presence of different concentrations of rotenone and H_2O_2. The upper band is the hyperphosphorylated Cyclin D1 and the lower band represents the hypophosphorylated Cyclin D1.
(B) … The graphs represent the densitometric analyses of Cyclin D1 (CyD1) expression in the presence of 500µM of rotenone and 500µM of H_2O_2 at the indicated time point.

Re:6 As correctly shown in Figure 2A, the IGF-IR activates both the MAPK and Akt pathways, which both regulate cyclin D1 levels. Does the IGF-IR activate the MAPK pathway in Murine dermal fibroblasts? If it does, it would be appropriate to show in Fig. 1 and 2 IGF-I-induced ERKs activation with or without Rotenone or H2O2. This is important because the effect of PTP1B on IGF-IR phosphorylation is going to affect not only the Akt pathway but the MAPK pathway as well. If it does not, it would be important to mention it in the Introduction. Please also modify the Synopsis accordingly.

We thank this Reviewer for her/his meaningful and constructive suggestions which have led to more in depth insight. We performed the suggested experiment addressing her/his question whether IGF-1

via the IGF-1R activate the MAPK pathway in murine dermal fibroblasts. In fact, as shown in Figure 2C exposure of murine dermal fibroblasts with recombinant murine IGF-1 did not activate (phosphorylate) ERK. Therefore we can conclude that IGF-1 does not activate ERK in murine dermal fibroblasts. Apparently, IGF-1 affects the AKT pathway but not the MAPK pathway in murine dermal fibroblasts. Interestingly, IGF-1 activation of AKT is suppressed by the combined application of IGF-1 and rotenone induced $O_2^{\cdot -}$, suggesting that rotenone, in fact, has impacts on IGF-1 signalling. By contrast, ERK activation was not affected by IGF-1 but reduced by rotenone independent of IGF-1 (Figure 2C).

We followed this reviewer´s suggestion and introduced our observation that IGF-1 did not induce ERK phosphorylation at the level of IGF-1R activation.

Revised version of the manuscript, Page 3, Line 29-33, Page 4, Line 1-3, Introduction Section:
In addition to the PI3K-AKT axis, IGF-1 signalling was reported to activate the mitogen activated protein kinase (MAPK) pathway thus exerting its prosurvival effect in many though not all cell lines (Parrizas et al, 1997; Peruzzi et al, 1999; Subramaniam et al, 2005). In fact, IGF-1 can also inhibit ERK activation in some cell types, including neurons (Subramaniam et al, 2005). Therefore, the effect of IGF-1 on MAPK and Cyclin D1 expression is not uniform for all cell types and most likely depends on the cell type, the nature, magnitude and duration of the stimulus.

Revised version of the manuscript, Page 7, Line 24-33, Page 9, Line 1, Result Section:
IGF-1 has no effect on activation of ERK in MDFs at least at 60 min post stimulation (Figure 2C), although there are reports of ERK activation by IGF-1 in other cell types (Parrizas et al, 1997; Peruzzi et al, 1999). In fact, IGF-1 induces the expression of Cyclin D1 (Figure 2B), but does not induce the activation of ERK (Figure 2C). The expression of Cyclin D1, which is suppressed after IGF-1 treatment in the presence of rotenone (Figure 2B) very much suggests that Cyclin D1 is not regulated by the IGF-1R induced ERK phosphorylation, but rather by IGF-1 induced AKT phosphorylation. The observed rotenone ($O_2^{\cdot -}$) mediated reduction of Cyclin D1 expression is possibly due to the inhibition of other pathways or effectors downstream of IGF-1R (Winston et al, 1996).

The effect of IGF-1 on ERK also has been added to Figure 2C in the revised version of the manuscript including the corresponding Figure legend, Results.

Revised version of the manuscript, Page 32, Line No. 13, Figure Legend Section:
Figure 2 (C) The effect of rotenone induced $O_2^{\cdot -}$ and H_2O_2 on IGF-1 stimulated AKT and ERK activation was studied by Western Blot analyses with actin serving as loading control.

Re:7 Figure 2, Panel C: the differences are not great and the blots are overexposed. Please add densitometric analysis. 4EBP1 blot shows at least two bands: which is the right one?

As recommended by the Reviewer, we have performed additional experiments to generate higher quality Western Blots on 4EBP1 expression. The 4EBP1 blot has been replaced with a new blot using a new antibody. The new 4EBP1 blot shows only one band. Densitometric analyses have been added in the revised figures (Figure 2D).

Re:8 Figure 2, Panel D: is H2O2 affecting IGF-I-dependent proliferation?

H_2O_2 also affects IGF-1 dependent proliferation. The data have been added in Figure 2E of revised manuscript (Figure 2E, Results).

Revised version of the manuscript, Page 32, Line No. 24-27, Figure Legend Section:
To study the role of $O_2^{\cdot -}$ and H_2O_2 on cell proliferation, quantification of Ki-67 (white bars) and BrdU incorporation (black bars) was performed in non-treated MDFs (control), IGF-1 (100 ng/ml), rotenone (100µM), H_2O_2 (100µM) and combined IGF-1/rotenone or IGF-1/H_2O_2 treated MDFs for 12 h.

Revised version of the manuscript, Page 8, Line No. 13-16, Result Section:

H_2O_2 at a concentration of 100 µM suppressed cell proliferation (Figure 2E and Supplementary Figure 2) and IGF-1 only partly attenuated the inhibitory effect of H_2O_2 on basal cell proliferation (Figure 2E and Supplementary Figure 2).

Re:9 The effect of Rotenone on IGF-IR phosphorylation is only shown in Fig. 4. This is an important piece of evidence and I would suggest showing it already in Fig. 2 B in conjunction with IGF-IR levels.

The effect of rotenone on IGF-1R phosphorylation which was originally shown in Figure 4A, is now shifted to Figure 2B in the revised manuscript, as recommended by this Reviewer.

Revised version of the manuscript, Page 7, Line No. 17, Result Section:
Addition of IGF-1 resulted in the activation (phosphorylation) of IGF-1Rb, AKT, ribosomal protein S6 (S6), and increased Cyclin D1 levels in MDFs (Figure 2B).

Revised version of the manuscript, Page 7, Line No. 21-24, Result Section:
Rotenone treatment markedly inhibited IGF-1 mediated IGF-1Rβ phosphorylation (Figure 2B), suggesting that enhanced O_2^- concentrations suppressed the IGF-1/AKT axis at the initial step of IGF-1 mediated IGF-1Rβ activation.

Re:10 Discussion, Page 13: "Enhanced O2•- concentrations occur in several physiological and pathological conditions" Please include some examples with references.

As recommended we included some examples of O_2^- mediated pathophysiological conditions in the Discussion section of revised manuscript.

Revised version of the manuscript, Page 14, Line No. 23-26, Discussion Section:
Enhanced O_2^- concentrations have been reported in several physiological and pathological inflammatory conditions, (Lu et al, 2003; Shishido et al, 1994; Naya et al, 1997; Macarthur et al, 2000; Cheng et al, 1999; Liu et al, 2004; Sindrilaru et al, 2011).

Re:11 Discussion, Page 16: One issue that is still somewhat puzzling is "how increased O2•- concentrations in the mitochondria transfer the signal to the cytoplasm" This issue is touched in the Discussion section but it may deserve a more extended discussion. Is there the possibility that O2•- may leak from the mitochondria to the cytosol?

Indeed this an excellent question. In spite of several and still continual efforts, it is not clear whether O_2^- leaks from the mitochondria to cytosol. A note on the escape of intramitochondrial O_2^- to the cytosol has been added in the revised manuscript.

Revised version of the manuscript, Page 17, Line No. 16-28, Discussion Section:
As to the question how increased $O_2^{\bullet-}$ concentrations in the mitochondria transfer the signal to the cytoplasm, it is possible that enhanced mitochondrial $O_2^{\bullet-}$ concentrations lead to modifications of kinases, which shuttle between the mitochondria and the plasma membrane exerting phosphorylation and activation of PTP1B and PTEN – the nature of these anticipated kinases remains, however, elusive. Alternatively, longer-lived lipid peroxidation intermediates may transmit the $O_2^{\bullet-}$-induced signals from the mitochondria to cytosolic and/or membrane bound PTP1B/PTEN. Notably, $O_2^{\bullet-}$ is able to initiate lipid peroxidation by itself, or after reacting with nitric oxide (NO) to form peroxynitrite ($ONOO^-$) (Bashan et al, 2009; Buetler et al, 2004). A recent report suggests that O_2^- can escape mitochondria and enter the cytosol through mitochondrial voltage dependent anion channel (Lustgarten et al, 2012).

Referee 2

Re:1 Along these lines, while the authors attribute the effects of rotenone on superoxide production, it's not clear this is the only thing this mitochondrial agent is doing. What about effects on ATP levels or substrate utilization? Can the authors demonstrate the specificity of their observations?

The authors agree with this Referee's comment that apart from mitochondrial complex 1 inhibition with accumulation of $O_2^{\cdot-}$ within the mitochondria, rotenone may also exert independent effects as discussed below (Referee 2, Re:2). Several lines of evidence support the conclusion that enhanced $O_2^{\cdot-}$ concentrations are causal and specific for the suppression of IGF-1 signalling. First, we have overexpressed and routed Sod2 to the mitochondria to detoxify $O_2^{\cdot-}$ and thus lowered its concentration in rotenone treated fibroblasts. In fact, Sod2 overexpression in rotenone treated murine dermal fibroblasts protects from rotenone mediated IGF-1 resistance (Figure 6). Thus, even in case rotenone mediates effects independent of intramitochondrial $O_2^{\cdot-}$ accumulation, the rescue of impaired IGF-1 signalling in Sod2 overexpressing rotenone treated fibroblasts strongly indicates that the rotenone–dependent intramitochondrail $O_2^{\cdot-}$ accumulation is at least partly responsible for the suppression of IGF-1 signalling. Secondly, Sod2 overexpression with detoxification of $O_2^{\cdot-}$ inhibited the translocation of PTEN and PTP1B to the plasma membrane which represents a crucial event in the activation of these two phosphatases. Third, we also observed a significant IGF-1 resistance in Sod2 deficient murine dermal fibroblasts, although the IGF-1 resistance was less compared to that of rotenone treated fibroblasts. Nevertheless, the lack of $O_2^{\cdot-}$ detoxification and subsequent enhanced $O_2^{\cdot-}$ concentrations are causal for IGF-1 suppression in Sod2 deficient fibroblasts *in vitro* (Figure 2 F) and similarly in fibroblast specific Sod2 deficient mice *in vivo* (Figure 3A). This Reviewer wondered whether decreased ATP levels could be affected by rotenone and thus may be responsible for the rotenone effect. Rotenone inhibits complex 1 and thus disrupts the oxidative phosphorylation and subsequently ATP generation. In fact, ATP-levels decreased in rotenone treated fibroblasts (data not shown). Even though rotenone expectedly decreased ATP concentrations, our finding that we can rescue IGF-1 signalling in Sod2 overexpressing rotenone treated fibroblasts very much support the conclusion that enhanced $O_2^{\cdot-}$ concentrations in the first instance and not decreased ATP levels disrupt IGF-1 signalling. This notion is also supported by the fact that rotenone inhibition of complex 1 persists in Sod2 overexpressing fibroblasts. We are very grateful to the constructive comments of this reviewer which helped to more explicitly put forward evidence for the specificity of enhanced $O_2^{\cdot-}$ concentrations in conferring IGF-1 resistance. Parts of the above discussion has been included into the revised manuscript in the Discussion section.

Revised version of the manuscript, Page 15, Line No. 26-33, Page 16, Line No. 1-12, Discussion Section:
…. we found that high $O_2^{\bullet-}$ concentrations following rotenone mediated inhibition of mitochondrial complex I or by silencing of $O_2^{\bullet-}$-dismutating mitochondrial Sod2 resulted in IGF-1 resistance. Indeed, we identified enhanced activities and membrane localization of PTP1B and PTEN following high $O_2^{\bullet-}$ load, and interestingly, this effect was abolished by overexpression of Sod2. IGF-1 resistance was also observed in Sod2 deficient fibroblasts with significant accumulation of mitochondrial $O_2^{\bullet-}$, but not of H_2O_2 as proven by ROS specific quantitative techniques (Treiber et al, 2011). Moreover, scavenging of mitochondrial $O_2^{\bullet-}$ by the overexpression of Sod2 in the mitochondria of rotenone treated murine dermal fibroblasts attenuated the observed IGF-1 resistance. Rotenone may not only suppress complex 1 of the electron transfer chain thereby enhancing intramitochondrial $O_2^{\bullet-}$ concentrations. From a previous publication rotenone has been implicated in enhancing the NADPH oxidase in microglial cells of the brain with enhanced $O_2^{\bullet-}$ release into the extracellular space with subsequent neuron degeneration (Gao et al, 2003). Even though we cannot exclude this or other rotenone effects in our study, we provide consistent evidence that the rotenone mediated inhibition of complex 1 with accumulation of high $O_2^{\bullet-}$ concentrations or $O_2^{\bullet-}$ accumulation by silencing of $O_2^{\bullet-}$ dismutating mitochondrial Sod2 resulted in IGF-1 resistance.

Re:2 Are other signalling pathways affected by rotenone treatment or is it just the IGF-1 pathway?

This reviewer addressed the important question of the specificity of rotenone in the regulation of the IGF-1 pathway. From a previous publication rotenone has been implicated in enhancing the NADPH oxidase in microglial cells of the brain with enhanced $O_2^{\bullet-}$ release into the extracellular space with subsequent neuron degeneration (Gao et al, 2003). Even though we cannot exclude this or other rotenone effects in our study, we provide evidence that the rotenone mediated inhibition of complex 1 with accumulation high $O_2^{\bullet-}$ concentrations or by silencing of $O_2^{\bullet-}$ dismutating mitochondrial Sod2 resulted in IGF-1 resistance. In case, NADPH oxidase is induced under rotenone treatment, we would expect enhanced $O_2^{\bullet-}$- release into the extracellular space. This would not be necessarily affected following Sod2 overexpression in rotenone treated fibroblasts, while Sod2 overexpression and dismutation of mitochondrial $O_2^{\bullet-}$ in rotenone treated fibroblasts rescues

the supressed IGF-1 signalling. Suppression of IGF-1 signalling explicitly occurs in rotenone treated fibroblasts. As Sod2 overexpression lowers intramitochondrial $O_2^{\bullet-}$- concentrations, but most likely does not primarily affect enhanced release of $O_2^{\bullet-}$ from fibroblasts into the extracellular space, it is unlikely that enhanced NADPH oxidase following rotenone treatment is predominantly responsible for the observed IGF-1 resistance. However, a combined action of enhanced intramitochondrial and extracellular $O_2^{\bullet-}$ concentrations cannot be fully excluded by our experiments.

In addition, we found that rotenone suppress ERK activation. This most likely is independent of the IGF-1 pathway as IGF-1 did not induce ERK activation (Figure 2 C).

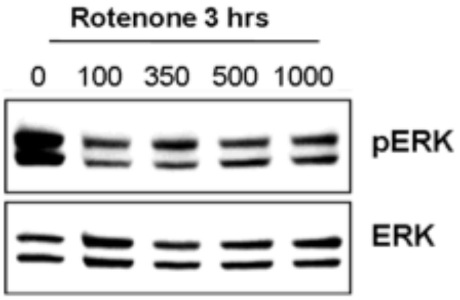

Collectively, we cannot exclude effects of rotenone on additional signalling pathways, however, our data that enhanced $O_2^{\bullet-}$ concentrations via activation of PTPB1 and PTEN dampen IGF-1 signalling are consistent.

Following this reviewer´s constructive criticism, we have adapted and broadened our discussion and do not claim that rotenone or enhanced $O_2^{\bullet-}$ concentrations exclusively affect IGF-1 signalling.

Revised version of the manuscript, Page 16, Line 3-12, Discussion Section:
Rotenone may not only suppress complex 1 of the electron transfer chain thereby enhancing intramitochondrial $O_2^{\bullet-}$ concentrations. From a previous publication rotenone has been implicated in enhancing the NADPH oxidase in microglial cells of the brain with enhanced $O_2^{\bullet-}$ release into the extracellular space with subsequent neuron degeneration (Gao et al, 2003). Even though we cannot exclude this or other rotenone effects in our study, we provide consistent evidence that the rotenone mediated inhibition of complex 1 with accumulation of high $O_2^{\bullet-}$ concentrations or $O_2^{\bullet-}$ accumulation by silencing of $O_2^{\bullet-}$ dismutating mitochondrial Sod2 resulted in IGF-1 resistance.

Revised version of the manuscript, Page 18, Line 21-24 Discussion Section
In addition, it will be exciting to further explore whether - in addition to the IGF-1 dampening - enhanced $O_2^{\bullet-}$ concentrations may affect other receptor tyrosine kinases involved in tissue homeostasis.

Re:3 One way of further solidifying the data would be to exploit the Sod2-/- system more. Can the results of Figure 4D and E be repeated using WT and Sod2 deficient cells? Another way would be to increase the quantitative aspect of the data. In that regard, can the main point of Figure 5 be quantitatively assessed?

We followed this reviewer´s recommendation to exploit the $Sod2^{-/-}$ system more to further support the rotenone data. We were able to provide *in vivo* evidence that in the case of a homozygous Sod2 deficiency in fibroblasts in the skin, IGF-1 signalling is significantly reduced (Figure 3A). The beauty of this experiment is that IGF-1 signalling at the level of AKT phosphorylation is specifically reduced in the dermal fibroblast-rich compartment, but not in the subcutaneous layer where the Sod2 expression is expected not to be reduced. In addition, stimulated by Reviewer 2 and 3, we were able to provide evidence that the strong atrophy/aging phenotype of the skin in fibroblast specific Sod2 deficiency *in vivo* can be rescued at least in part by the concomitantly occurring heterozygous PTEN deficiency. These data suggest that Sod2 deficiency with the accumulation of high $O_2^{\bullet-}$

concentrations with their activating effect on PTEN can be rescued in fibroblast specific Sod2$^{-/-}$/PTEN$^{+/-}$ mice (Figure 8). Of note, IGF-1 injection in the Sod2$^{-/-}$/Pten$^{+/-}$ mice result in significantly higher IGF-1 signalling when compared to IGF-1 injection in Sod2$^{-/-}$ mice (Figure 8C). The membrane translocation of PTP1B and PTEN was quantitatively assessed and added in the Supplementary Figure 5 of revised manuscript.

Re:4 Given the short half life of superoxide anions and their inability to pass through lipid bilayers, I'm not sure how the model shown in Figure 7 makes sense.

This Reviewer critically discuss the ability of intramitochondrial $O_2^{\bullet-}$ to pass the lipid membrane. This view has been broadly accepted, while indirect recent evidence point to possibility that intramitochondrial $O_2^{\bullet-}$ may at least in part leak from the mitochondria into the cytosol through mitochondrial voltage dependent anion channels (see also Reviewer 1; Re: 11). These recent insights and alternatives for the signal transfer of high intramitochondrial $O_2^{\bullet-}$ concentrations into the cytosol have been discussed in our manuscript.

Revised version of the manuscript, Page 17, Line 16-28, Discussion Section
As to the question how increased $O_2^{\bullet-}$ concentrations in the mitochondria transfer the signal to the cytoplasm, it is possible that enhanced mitochondrial $O_2^{\bullet-}$ concentrations lead to modifications of kinases, which shuttle between the mitochondria and the plasma membrane exerting phosphorylation and activation of PTP1B and PTEN – the nature of these anticipated kinases remains, however, elusive. Alternatively, longer-lived lipid peroxidation intermediates may transmit the $O_2^{\bullet-}$-induced signals from the mitochondria to cytosolic and/or membrane bound PTP1B/PTEN. Notably, $O_2^{\bullet-}$ is able to initiate lipid peroxidation by itself, or after reacting with nitric oxide (NO) to form peroxynitrite ($ONOO^-$) (Bashan et al, 2009; Buetler et al, 2004). A recent report suggests that O_2^- can escape mitochondria and enter the cytosol through mitochondrial voltage dependent anion channel (Lustgarten et al, 2012).

However, all these above discussed possibilities of how the intramitochondrial $O_2^{\bullet-}$ signal may be transferred into the cytosol are currently somewhat indirect or speculative, therefore we have followed the suggestion forwarded from Reviewer 2, and more cautiously used stippled lines and a question mark to indicate that the mechanism of this signal transfer are not fully understood (summary sketch, Figure 9).

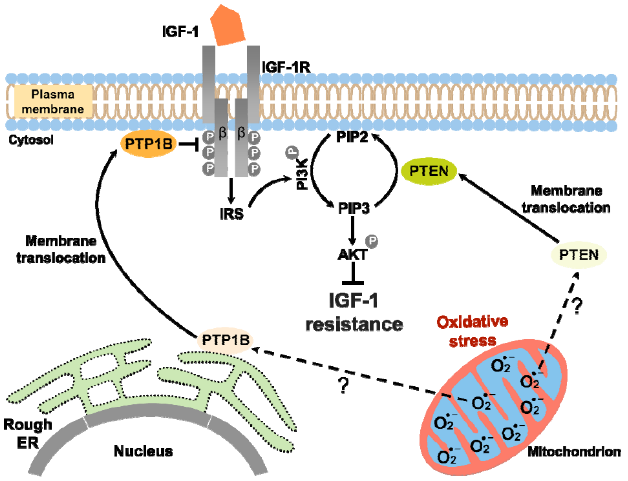

Figure 9. Summary Sketch of the Events Leading to IGF-1 resistance.

Note the signal transfer from intramitochondrial $O_2^{\bullet-}$- signal transfer into the cytosol is depicted now by a stippled line and a questionmark to indicate that the underlying mechanism is not fully elucidated.

Re:5 Again, this all revolves around my difficulty in accepting the basic premise that the authors have created a pure superoxide anion model by treating cells with rotenone.

This comment is centered around the theme already addressed by this Reviewer in Question 1.), 2.), 4.) We appreciate this Reviewer´s critical though constructive comments, and we accept that results deriving from the mere application of rotenone would be difficult to interpret and would not allow to draw solid conclusions. Accepting this difficulty, we have put major effort in complementing and solidifying the rotenone data. First, we have routed and enhanced Sod2 activity by a Sod2 overexpressing vector of rotenone treated murine fibroblasts and were able to rescue the IGF-1

© EMBO

resistance. Along this line, many of the rotenone effects such as lower phosphorylation of AKT, membrane translocation of PTP1B, PTEN can be significantly restored towards wildtype by overexpression of Sod2. Secondly, in the fibroblast specific Sod2 deficient mice – due to enhanced superoxide anion concentrations - we saw a reduced collagen deposition and skin atrophy, which can be partly rescued when the fibroblast specific Sod2 deficient mice are crossed to a fibroblast specific PTEN heterozygous deficient mice. In this $Sod2^{-/-}/PTEN^{+/-}$ mice we could partly rescue the atrophy of the skin, supporting the view that O_2^- exert a stimulatory effect on PTEN thereby dampening IGF-1 signalling as shown with reduced numbers of cells showing Akt phosphorylation or phosphorylation of the downstream effector SK6 kinase indicative of active IGF-1 signalling.
We discussed alternative effector mechanisms of rotenone action (see our answer to this Reviewer 2, Re:1, Re:2). We do hope that by adding complementary evidence as suggested by this Reviewer we have strengthened our conclusions and in addition managed to tone down too exclusice conclusions. This may hopefully stimulate exciting research in the short perspective.

Referee 3

Re:1 However, for some reason the authors confine important control data (that is relatively non-quantitative) that relates to the rescue of the phenotype following SOD2 overexpression to supplementary data (suppl Fig 5). This could be better placed in the main part of the paper.

We agree to this Reviewer´s suggestion that the data on SOD2 overexpression in rotenone treated fibroblasts which led to a complete rescue of IGF-1 dampening in IGF-1/rotenone treated mice should be placed in the main manuscript. These data, in fact, constitute an important control and allow the conclusion that enhanced concentrations of mitochondrial O_2^- are causal for the observed IGF-1 resistance. Therefore, as suggested by this Reviewer, we moved this important control to Figure 6 in the revised version of our manuscript.

Re:2 However, the key experiment in my view, and within the capability of the group, is whether homozygote or heterozygote suppression or reversal of Pten or Ptp1b reverses or alleviates the phenotype of homozygote SOD2 deficiency in their conditional mouse model.

We are grateful to this Reviewer for his valid suggestion, though it took several months to have the suggested mouse model ready for investigation. We followed the suggestion of this Reviewer and crossed fibroblast (connective tissue) specific Sod2 deficient mice with fibroblast specific PTEN heterozygous deficient mice and studied whether an alleviation or reversal of the phenotype (skin atrophy) of the fibroblast specific $Sod2^{-/-}$ mice occurred in mice with $Sod2^{-/-}$ / $Pten^{+/-}$ deficient fibroblasts. Interestingly, we observed that heterozygous deficiency of PTEN significantly protected the skin phenotype of Sod2 deficient mice. Fibroblasts specific homozygous Sod2 deficiency resulted in skin atrophy with severe reduction in the vertical thickness of epidermis, dermis, subcutaneous and panniculus carnosus layer of skin. Heterozygous deletion of PTEN in homozygous Sod2 deficient mice significantly rescued the skin atrophy compared with Sod2 homozygous deficient mice. In addition to this, heterozygous deletion of PTEN as well as inhibition of PTP1B using specific inhibitor also attenuate the impaired IGF-1 signalling *in vivo* in the Sod2 homozygous deficient mice.

Revised version of the manuscript, Page 5, Line No. 27-31, Introduction Section:
This was confirmed *in vivo* by the rescue of the skin aging phenotype and IGF-1 signalling in fibroblast specific Sod2 deficient mice, where PTEN gene was heterozygously deleted in Sod2 deficient mice. Similarly, pharmacologic PTP1B inhibition in Sod2 deficient mice showed partial reversal of the impaired IGF-1 signalling.

Revised version of the manuscript, Page 12, Line No. 20-33, Page 13, Line No. 1-9 Result Section:
To further explore whether our *in vitro* observations may also have relevance *in vivo* for tissue homeostasis, we used a genetic approach. Deletion of Sod2 in fibroblasts of the connective tissue rich skin resulted in skin atrophy, a key feature of aging, with reduced thickness of all the skin layers (Figure 8A and B). Heterozygous deficiency of PTEN in the Sod2 deficient fibroblasts as achieved by crossing PTEN floxed mice with Col(I)a2-CreERT transgenic and Sod2 floxed mice ($Col(I)a2CreERT^{+};Sod2^{f/f}$) followed by activation of CreERT with tamoxifen treatment

(Supplementary Figure 8A and B), rescued skin atrophy/aging in the double mutant mice (Col(I)a2-CreERT^{+};Sod2$^{f/f}$;PTEN$^{f/+}$) compared with Sod2 mutant mice (Col(I)a2CreERT^{+};Sod2$^{f/f}$;PTEN$^{+/+}$) (Figure 8A and B). Of note, genetic heterozygous deficiency of PTEN with reduced PTEN expression or inhibition of PTP1B by specific inhibitor also attenuated the IGF-1 resistance *in vivo* in a Sod2 deficient murine aging model (Figure 8C and D). Accordingly, heterozygous PTEN deletion rescued IGF-1 stimulated phosphorylation of AKT in the skin of fibroblasts specific Sod2 deficient mice (Figure 8C and D). Similarly, continuous inhibition of PTP1B with its specific inhibitor at a dose of 15 mg/kg for 5 days also rescued IGF-1 induced phosphorylation of AKT in the skin of fibroblasts specific Sod2 deficient mice (Figure 8C and D). These data show that enhanced $O_2^{.-}$- concentrations in dermal fibroblasts in the skin *in situ* - via PTEN and PTP1B activation - dampen the IGF-1 signalling also *in vivo*, and most likely contribute to skin aging/atrophy.

Revised version of the manuscript, Page 17, Line No. 3-5, Discussion Section:
Importantly, partial deletion of PTEN and inhibition of PTP1B in Sod2 deficient mice also rescued the IGF-1 resistance and the atrophy/aging skin phenotype.

Re:3 Moreover, in addition, the main data presented do not appear to be all that significant, as most of the effects were of the order of two fold and have been variably analysed statistically with either two way or one way analysis. For this reason also, the in vivo magnitude of the effects over time would be more informative than acute responses to maximal IGF1 stimulation at fixed time points.

We followed the Reviewer's recommendation and monitored the effects of fibroblasts specific Sod2 deficiency in a murine aging model over time. As shown in Figure 8A and B, in the case of the double mutant mice (Sod2$^{-/-}$; PTEN$^{+/-}$), it was possible to almost completely rescue the skin atrophy/aging phenotype which was observed in the mutant mice (Sod2$^{-/-}$). These data, in fact, indicate that heterozygous PTEN deficiency does play a role in rescuing the atrophy phenotype. Also in the double mutant mice (Sod2$^{-/-}$; PTEN$^{+/-}$) and in the mutant mice which were treated with the PTP1B inhibitor *in vivo* (Sod2$^{-/-}$ + PTP1B inhibitor) a strong rescue of IGF-1 signalling in terms of phosphorylated AKT (pAKT) levels was observed when compared to the pAKT levels of mutant mice (Sod2$^{-/-}$) (Figure 8C and D). In addition to the long term effects without IGF-1 injection in the mutant and double mutant mice, we have also focused on the role of enhanced $O_2^{.-}$- concentrations in PTEN and PTP1B activation after IGF-1 injection to forward strong proof-of-principle experiments that, in fact, enhanced $O_2^{.-}$- concentrations via PTEN and PTP1B activation suppress IGF-1 signalling and lead to a skin atrophy/aging phenotype also *in vivo*. We think that these data, the generation of which has been inspired by Reviewer 3, as such are exciting and previously unreported. In this experimental proof-of-principle setting, we exploited the beauty of controlled IGF-1 injections. This experimental design allowed us to easily monitor IGF-1 signalling and decipher the *in vivo* role of enhanced $O2^{.-}$- concentrations and its causal relationship to PTEN and PTP1B activation, subsequent suppression of IGF-1 signalling and functional consequences such as skin atrophy and aging. The Reviewer suggested to follow the effect of IGF-1 signalling not only at a fixed time point. Therefore we have added data of the 60 min time point after IGF-1 i.p. injection of genetically defined mice in the revised manuscript (Supplementary Figure 4C). Of note, the maximum IGF-1 stimulation with phosphorylation of AKT is observed already after 15 min of i.p. IGF-1 injection, the magnitude of response of IGF-1 stimulation reduces thereafter and almost no difference between the saline and IGF-1 injected groups was detected after 60 min of stimulation. We added these data, as the phenotypic response of rescuing skin atrophy in the setting of enhanced $O_2^{.-}$- concentrations and diminished PTEN or PTP1B activity may be particularly interesting for the development of short term preventive or therapeutic strategies.

Revised version of the manuscript, Page 9, Line No. 19-22, Result Section:
The maximum stimulatory effect of IGF-1 was found 15 min after injection, which was no longer detectable at 60 min after IGF-1 injection (Figure 3B and Supplementary Figure 4C).

Revised version of the manuscript, Page 46, Line No. 27-32, Page 47, Line No. 1-2, Figure legend Section:
(Supplementary Figure 3C) Skin lysates from control mice (with normal $O_2^{.-}$ concentrations) and mutant mice (with enhanced $O_2^{.-}$ concentrations in fibroblasts) were prepared 60 min after i.p. injection of 1mg/ml IGF-1 or saline and the expression of pAKT (S473) and AKT was analysed by

Western blotting and equilibrated to actin expression levels. A representative Western blot out of 3 independent experiments is shown here. Graph (right panel) depicts densitometric analyses of pAKT/AKT ratio after correction with actin (loading control) for each of the four groups.

Minor comments

Re:1 The mouse model uses a prolonged Cre activation to delete SOD2, and the response to IGF1 injections is only carried out at one time point. The quantification of response is only presented after a short time in Figure 3A and 3B. Consideration should be given to at least another time point and explanation given for poor efficiency and need for continued Cre activation.

We observed that the highest deletion efficiency of Col(I)a2-CreERT can be achieved when CreERT induction is continued for at least 40-50 days after birth. Several reasons led us to continue with the 4-OH tamoxifen treatment for this time period directly after birth. First, it is crucial to keep a steady state concentration of 4-OH tamoxifen for the required recombination in fibroblasts in mice. Second, *in vivo* tamoxifen-inducible Cre mediated recombination is not 100% efficient compared to promoters driving constitutively active Cre mediated recombination. This consistently results in a mosaic effect in terms of CreERT mediated recombination. The survival of few fibroblasts without recombination may be enhanced by no or less availability of 4-OH tamoxifen. These non-recombined fibroblasts will also proliferate and may dilute effects of recombined fibroblasts. Third, during the initial postnatal phase of 40-50 days fibroblast proliferation and collagen synthesis is much higher compared to later phases. As the collagen promoter drives the Cre recombinase, it is essential to exploit this phase. Fourth, new fibroblasts with no deletion/recombination may also originate from mesenchymal progenitor cells of the skin, where the CreERT is not expressed. In order to activate the CreERT in these newly formed fibroblasts, continued 4-OH tamoxifen treatment is necessary.

We followed this Reviewer´s recommendation to at least provide a second time point after IGF-1 injection. In addition to the 15 min time point, we now added data of Akt phosphorylation 60 min after IGF-1 stimulation in the revised manuscript.

Revised version of the manuscript, Page 9, Line No. 19-22, Result Section:
The maximum stimulatory effect of IGF-1 was found 15 min after injection, which was no longer detectable at 60 min after IGF-1 injection (Figure 3B and Supplementary Figure 4C).

Revised version of the manuscript, Page 46, Line No. 27-32, Page 47, Line No. 1-2, Figure legend Section:
(Supplementary Figure 3C) Skin lysates from control mice (with normal O_2^- concentrations) and mutant mice (with enhanced O_2^- concentrations in fibroblasts) were prepared 60 min after i.p. injection of 1mg/ml IGF-1 or saline and the expression of pAKT (S473) and AKT was analysed by Western blotting and equilibrated to actin expression levels. A representative Western blot out of 3 independent experiments is shown here. Graph (right panel) depicts densitometric analyses of pAKT/AKT ratio after correction with actin (loading control) for each of the four groups.

Re:2 P4. Sentence beginning; "Also mouse lacking...." Does not make sense.

According to the Reviewer´s advice, the sentence has been modified in the revised manuscript.

Revised version of the manuscript, Page 4, Line No. 27-28, Introduction Section:
In addition, it was reported that mice deficient of glutathione peroxidase (Gpx), the key enzyme responsible for H_2O_2 detoxification, were distinctly protected from high fat-induced insulin resistance (Loh et al, 2009).

Re:3 P8 para2 line 3, MDF or MFD?

It is murine dermal fibroblasts (MDF), the 'MFD' in the original manuscript was a typographical error and has been corrected in the revised manuscript.

Revised version of the manuscript, Page 8, Line No. 19, Result Section:
These MDFs revealed a marked reduction in pAKT compared to Sod2 competent murine MDFs (Figure 2E, left panel).

Re:4 P21 10_days
In the revised manuscript, a space has been added between '10' and 'days'

Revised version of the manuscript, Page 44, Line No. 7, Materials and methods Section:
To initiate the deletion of Sod2, newborn mice (10 days old) were orally fed with 4-OH tamoxifen (Sigma) suspended in 0.5% methylcellulose and 0.1% Tween 80 (Sigma) for 5 alternate days.

Re:5 P31 Figure 2D legend, "To role..." does not make sense?
In the revised manuscript, this sentence was corrected.

Revised version of the manuscript, Page 32, Line No. 14, Figure legend Section, Figure 2D:
(D) To dissect whether enhanced $O_2^{\cdot-}$ concentrations affect IGF-1 dependent effectors involved…

Re: 6 Fig 2E, over contrast on western blots. Have these been imaged enhanced?

In figure 2E, due to very low level of phosphorylated AKT (pAKT), the membrane was exposed for a long time. Other Western blots were exposed for short time.

2nd Editorial Decision 30 October 2014

Thank you for the submission of your revised manuscript to EMBO Molecular Medicine. We have now received the enclosed reports from the Reviewers who were asked to re-assess it.

As you will see, while they are now globally supportive, Reviewer 2 is still concerned that the concentration of rotenone used was quite high and wonders whether the concentration is correct as stated, and if it is, would like a clear explanation of why this was done. I agree that is an important point that needs clarification and based on your reply, we will decide whether to send back to the Reviewer for final approval.

Should your manuscript be accepted for publication, I will be asking of you a number of amendments as listed below. If you wish, you can directly do so with the next final revision to save time:

Please submit your revised manuscript within two weeks. I look forward to seeing a revised form of your manuscript as soon as possible.

***** Reviewer's comments *****

Referee #1 (Remarks):

The authors have carefully addressed the concerns raised in the previous review cycle. The paper has been considerably improved.

Referee #2 (Remarks):

Overall, the authors have addressed my concerns. The addition of new genetic approaches have, in my mind, solidified the results. I do have one remaining concern. The concentration of rotenone used was several hundred micromolar. Most published experimental paradigms use closer to 1 micromolar. Are the rotenone concentrations used correct as stated? If they are, why are the authors

using a dose that appears to two orders of magnitude higher than needed to inhibit Complex I? Some rationale is needed to explain this large variation from what is customary.

Referee #3 (Comments on Novelty/Model System):

The model system is greatly improved by the introduction of the genetic cross with pten+/- and PTB1B inhibitors.

Referee #3 (Remarks):

The authors are to be congratulated on timely modifications of the manuscript in light of the comments. In particular it appears the Pten+/- reduction of the Sod2-/- phenotype in the skin provides a substantial improvement in the paper and its conclusions. The in vitro observations are therefore substantiated by the in vivo model, and the time dependent responses reported (even if the effects are potentially transient) and the other minor comments addressed. Further analysis and questions remain for the authors, but the body of work is substantial and the conclusions are in keeping with the data.
There are no other modifications and comments to be added by this referee.

2nd Revision - authors' response 06 November 2014

Referee 1

The authors have carefully addressed the concerns raised in the previous review cycle. The paper has been considerably improved.

The authors cordially thank Referee 1 for his constructive comments and suggestion to improve the manuscript.

Referee 2

Overall, the authors have addressed my concerns. The addition of new genetic approaches have, in my mind, solidified the results. I do have one remaining concern. The concentration of rotenone used was several hundred micromolar. Most published experimental paradigms use closer to 1 micromolar. Are the rotenone concentrations used correct as stated? If they are, why are the authors using a dose that appears to two orders of magnitude higher than needed to inhibit Complex I? Some rationale is needed to explain this large variation from what is customary.

Referee 2 raised an important question. The rotenone concentrations, which were used in our experiments were based on a kinetic of different rotenone concentrations on murine dermal fibroblasts (MDF) and the formation of superoxide anion, which was monitored by the emission of fluorescence from the superoxide anion specific MitoSOX. We have included the kinetics of superoxide anion generation depending on different rotenone concentrations in Supplementary Figure 1B. Supplementary Figure 1B depicts that a significant intramitochondrial accumulation of superoxide anions is achieved with rotenone concentrations ranging from 350 to 1000 µM. Previously, using MitoSOX fluorescence, we found that Sod2 deficient murine dermal fibroblasts (MDF) displayed a more than 2 fold increase in superoxide anion concentrations compared with Sod2 competent murine dermal fibroblasts (Treiber et al, 2011). In order to achieve a comparable accumulation of intramitochiondrial superoxide anions in control murine dermal fibroblasts, we have used ~500µM of rotenone (Supplementary Figure 1B). Our main intention was to generate the intramitochondrial superoxide anion concentration that was found in Sod2 mutant MDF to study the effect of enhanced superoxide anion concentrations on the IGF-1 signalling. Although rotenone may transiently inhibit complex I at lower concentrations ranging from 1µM to 25µM in some cell types as stated by Referee 2, there are many reports that have employed higher rotenone concentrations ranging from 50-300µM to long term maintain enhanced superoxide concentrations (Furukawa et al., 2004, Sim et al., 2005, Ahn et al., 2008, Siddiqui et al., 2011, Srivastava et al., 2014). In addition to cell type specific differences in rotenone concentration requirement to generate superoxide

anions, we think that rotenone dissolved in chloroform as done in our experiments (avoiding DMSO formation which potentially scavenge reactive oxygen species), may decrease the bioavailability of rotenone when compared to experimental settings with rotenone not dissolved in chloroform.

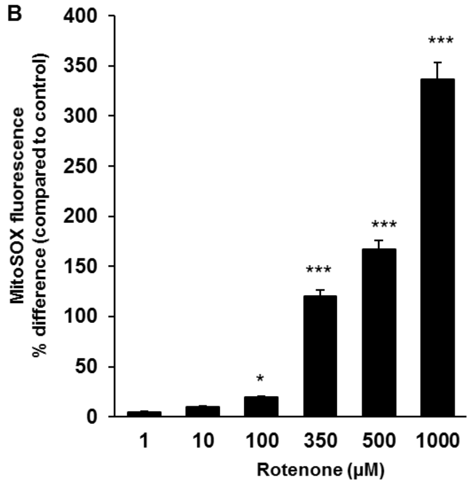

Supplementary Figure 1B: Mitochondrial O_2^{-} concentrations were measured by the superoxide anion specific MitoSOX dye in living cells. The graph depicts emission fluorescence intensities measured at ~580 nm wavelength range (excitation 396 nm) in MDFs treated with rotenone at the indicated concentrations (10 to 1000 μM) for 30 min. Values are presented as mean ± SEM of percent difference of fluorescence with the non-treated control MDFs set as zero. Comparison was made with one-way ANOVA followed by Bonferroni correction. * $P < 0.05$, *** $P < 0.001$, compared with MDFs in the absence of rotenone.

References

Treiber N, Maity P, Singh K, Kohn M, Keist AF, Ferchiu F, Sante L, Frese S, Bloch W, Kreppel F et al (2011) Accelerated aging phenotype in mice with conditional deficiency for mitochondrial superoxide dismutase in the connective tissue. Aging Cell 10: 239-254.

Furukawa S, Fujita T, Shimabukuro M, Iwaki M, Yamada Y, Nakajima Y, Nakayama O, Makishima M, Matsuda M, Shimomura I (2004) Increased oxidative stress in obesity and its impact on metabolic syndrome. J Clin Invest 114: 1752-1761.

Sim S, Yong TS, Park SJ, Im KI, Kong Y, Ryu JS, Min DY, Shin MH (2005) NADPH oxidase-derived reactive oxygen species-mediated activation of ERK1/2 is required for apoptosis of human neutrophils induced by Entamoeba histolytica. Journal of immunology 174: 4279-4288.

Ahn BH, Kim HS, Song S, Lee IH, Liu J, Vassilopoulos A, Deng CX, Finkel T (2008) A role for the mitochondrial deacetylase Sirt3 in regulating energy homeostasis. Proc Natl Acad Sci U S A 105: 14447-14452.

Siddiqui MA, Saquib Q, Ahamed M, Ahmad J, Al-Khedhairy AA, Abou-Tarboush FM, Musarrat J (2011) Effect of Trans-resveratrol on Rotenone-induced Cytotoxicity in Human Breast Adenocarcinoma Cells. Toxicology international 18: 105-110.

Srivastava N, Kollipara RK, Singh DK, Sudderth J, Hu Z, Nguyen H, Wang S, Humphries CG, Carstens R, Huffman KE et al (2014) Inhibition of Cancer Cell Proliferation by PPARgamma Is Mediated by a Metabolic Switch that Increases Reactive Oxygen Species Levels. Cell metabolism 20: 650-661.

Referee 3

(Comments on Novelty/Model System):
The model system is greatly improved by the introduction of the genetic cross with pten+/- and PTB1B inhibitors.

(Remarks):
The authors are to be congratulated on timely modifications of the manuscript in light of the comments. In particular it appears the Pten+/- reduction of the Sod2-/- phenotype in the skin provides a substantial improvement in the paper and its conclusions. The in vitro observations are therefore substantiated by the in vivo model, and the time dependent responses reported (even if the effects are potentially transient) and the other minor comments addressed. Further analysis and questions remain for the authors, but the body of work is substantial and the conclusions are in keeping with the data.

There are no other modifications and comments to be added by this referee.

The authors appreciate Referee 3 for his excellent comments and suggestion on necessity of *in vivo* models and validation of the *in vitro* finding in the *in vivo* systems. This has improved our manuscript substantially.

3rd Editorial Decision 13 November 2014

We are pleased to inform you that your manuscript is accepted for publication and is now being sent to our publisher to be included in the next available issue of EMBO Molecular Medicine.

© EMBO
