# Supplementary figures and images for: Superoxide anion radicals induce IGF-1 resistance through concomitant activation of PTP1B and PTEN
Source: EMBO Mol Med. 2014 Dec 17;7(1):59–77. doi: 10.15252/emmm.201404082 (PMC4309668; doi:10.15252/emmm.201404082)

**Figure 1**

**A**

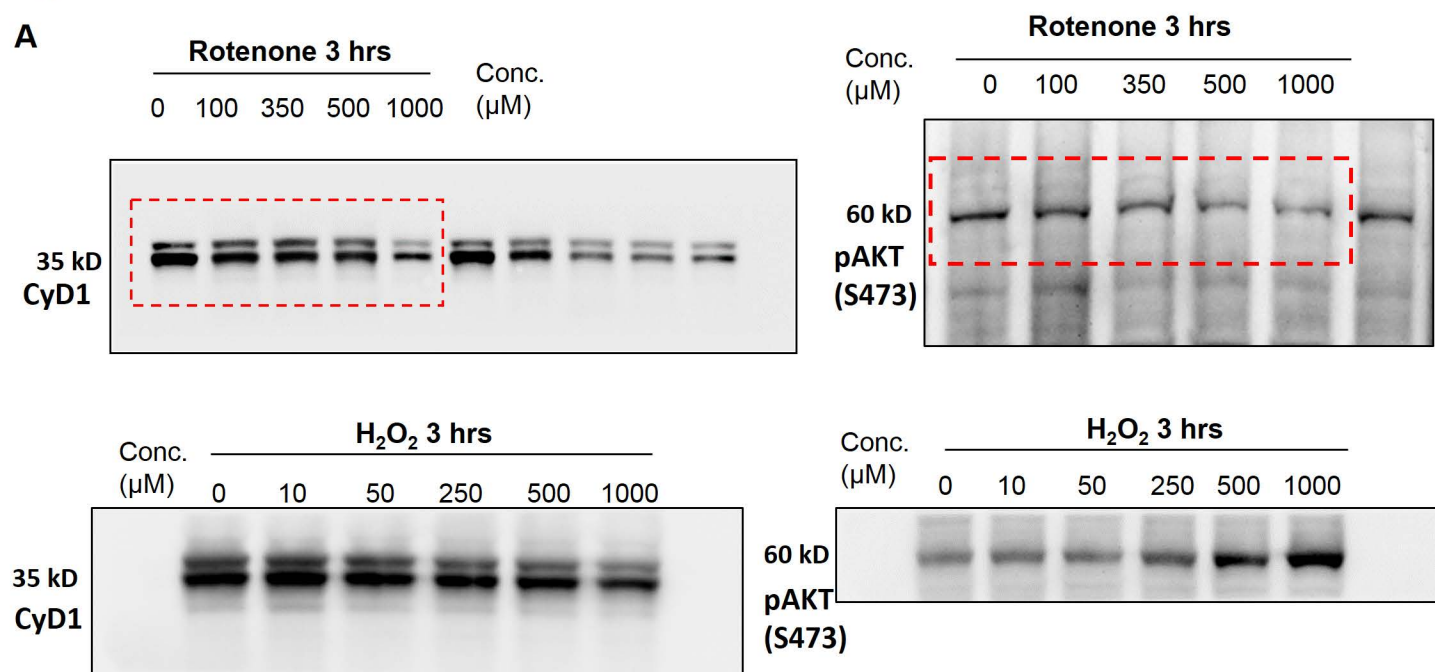

**B**

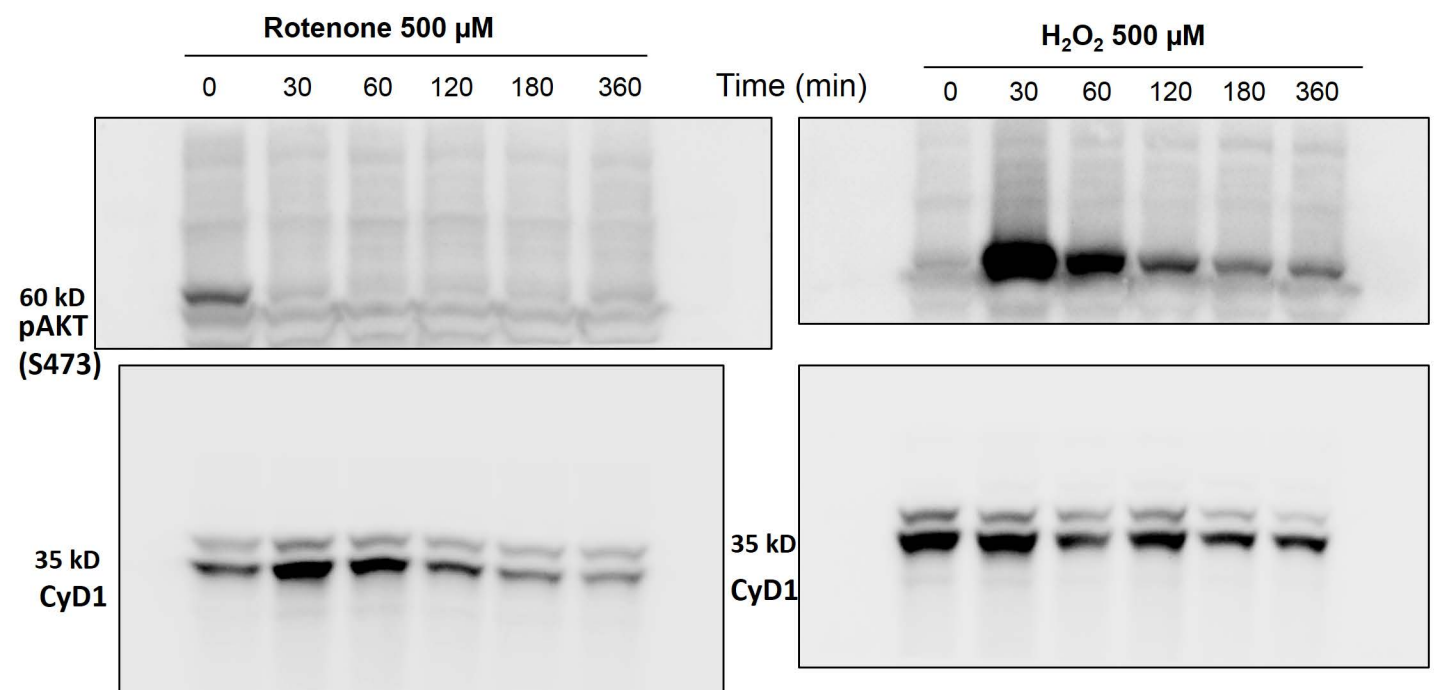

Supplement: Supplementary file 3 [file emmm0007-0059-sd3.pdf]

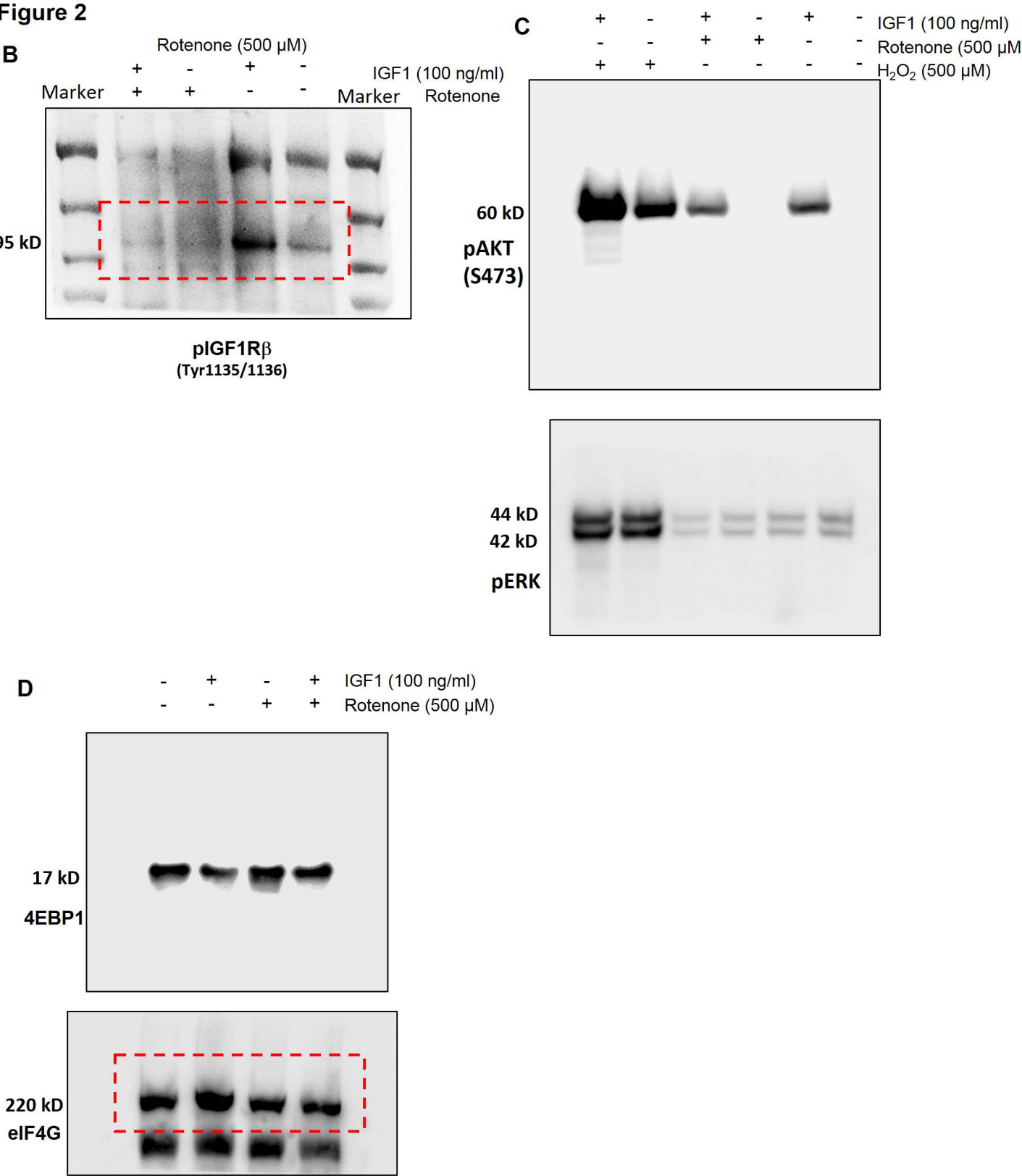

Supplement: Supplementary file 4 [file emmm0007-0059-sd4.pdf]

Figure 3

B

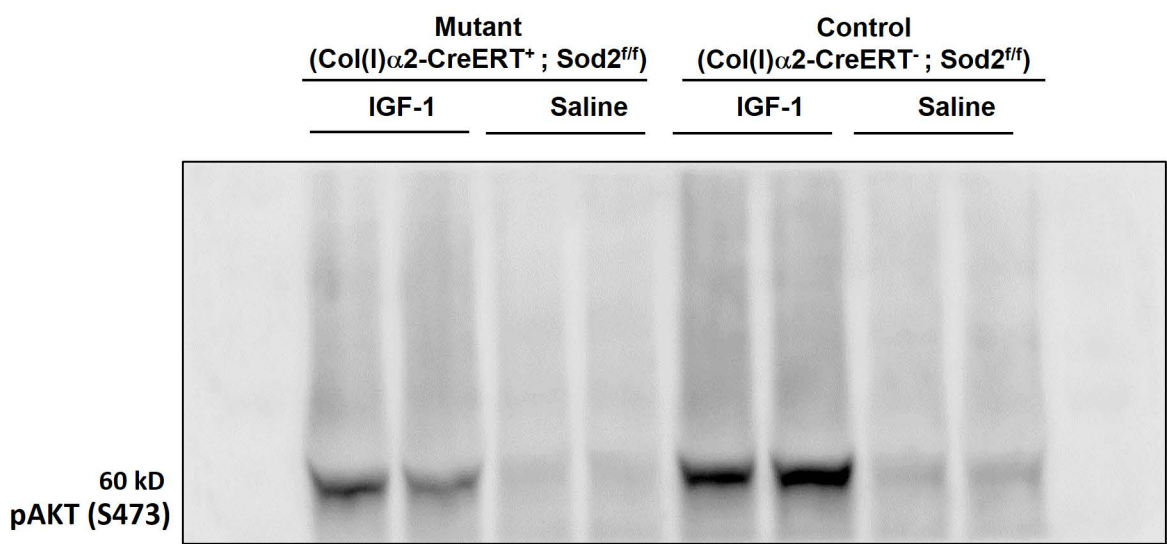

Supplement: Supplementary file 5 [file emmm0007-0059-sd5.pdf]

**Figure 5**

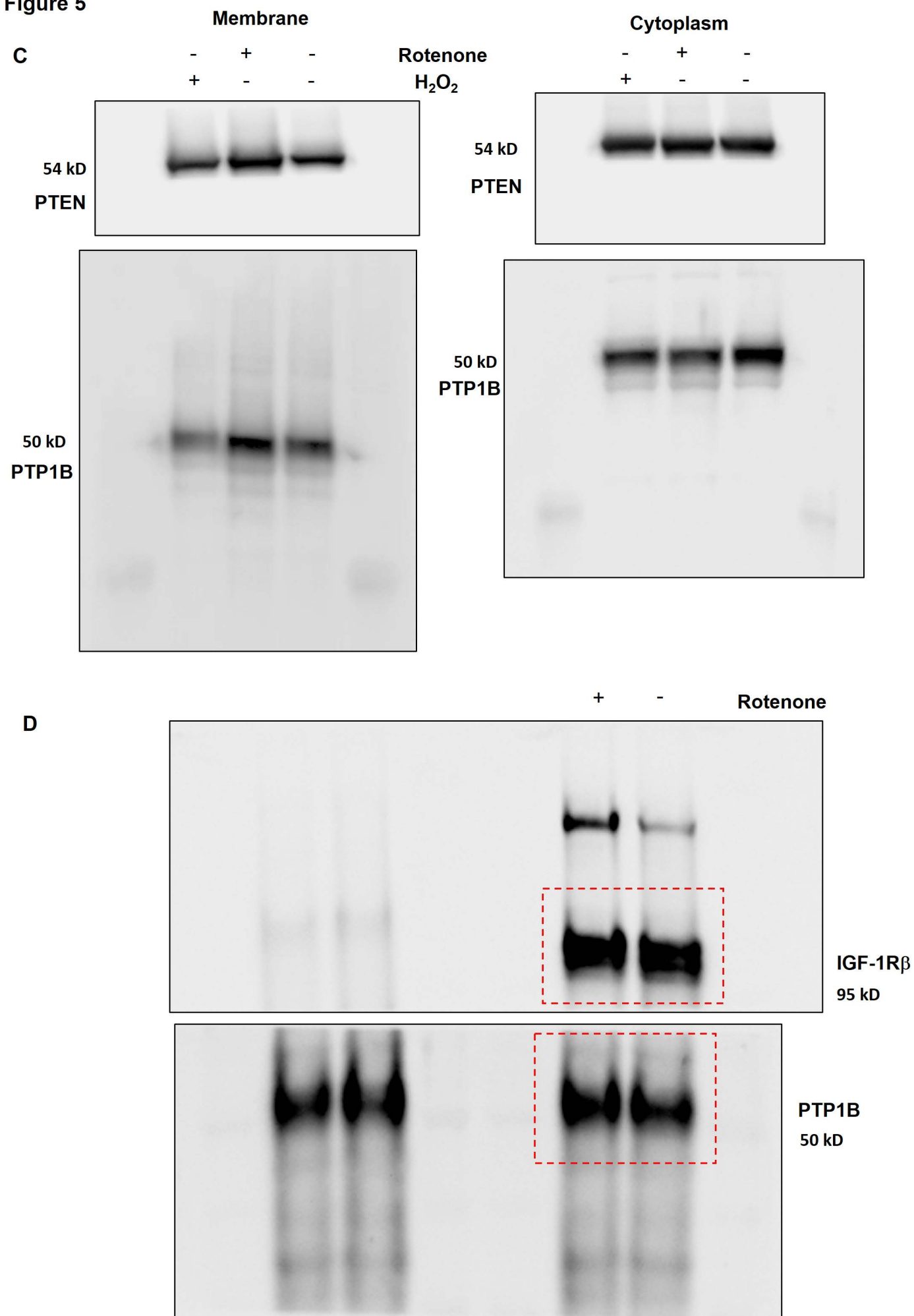

Supplement: Supplementary file 6 [file emmm0007-0059-sd6.pdf]

**Figure 6**

**C**

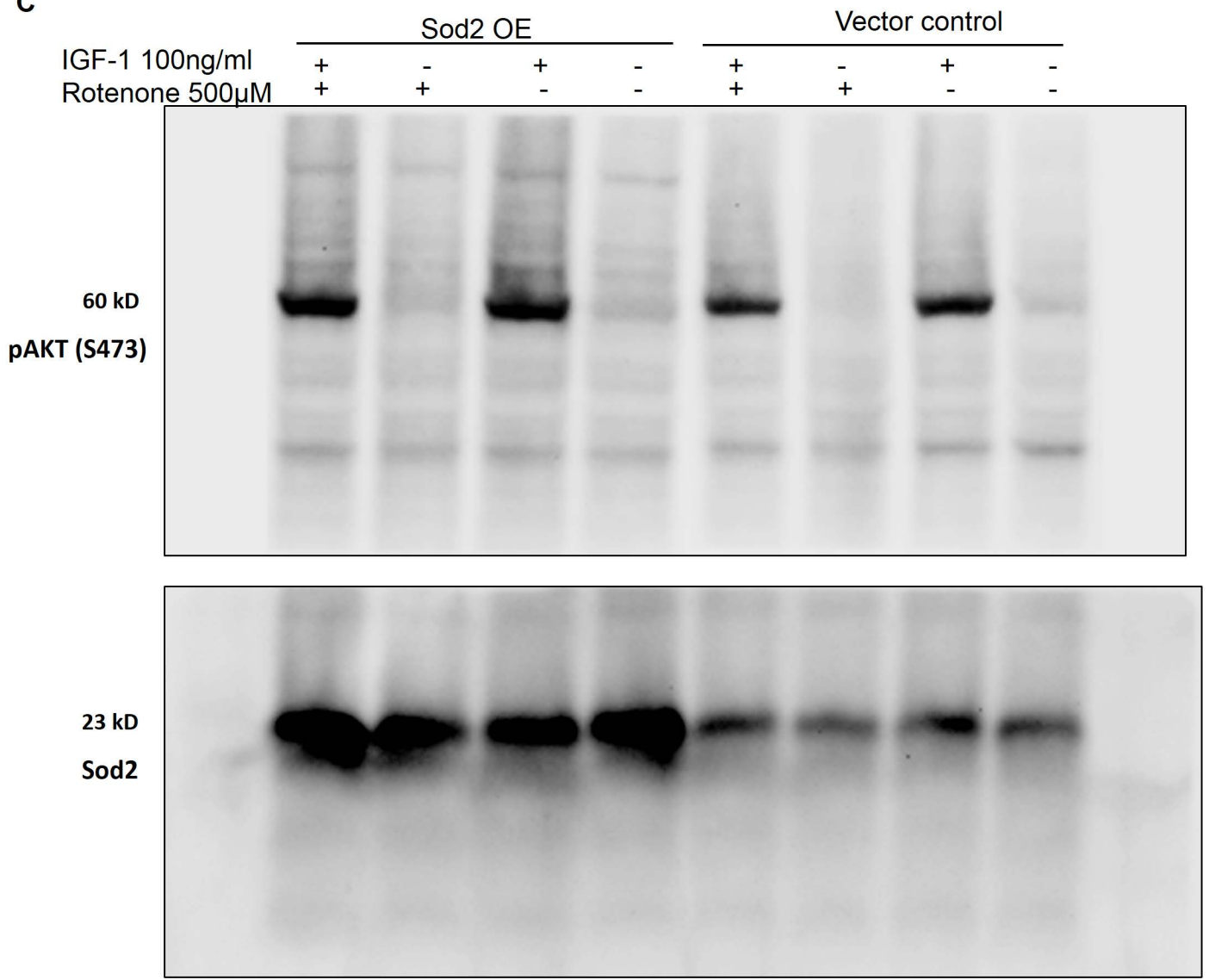

Supplement: Supplementary file 7 [file emmm0007-0059-sd7.pdf]

Figure 7

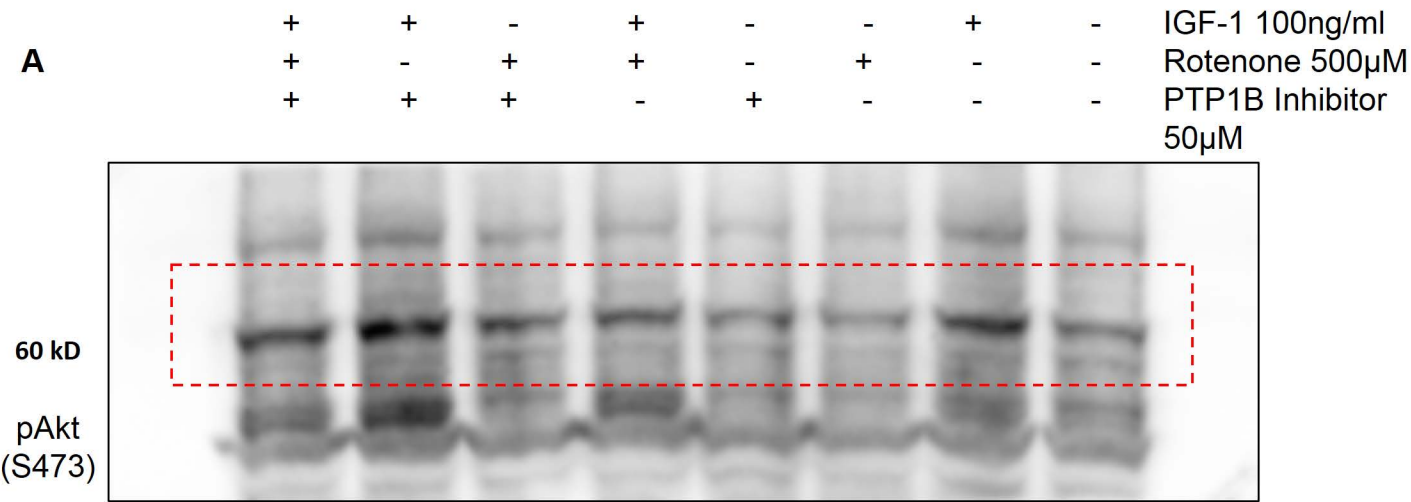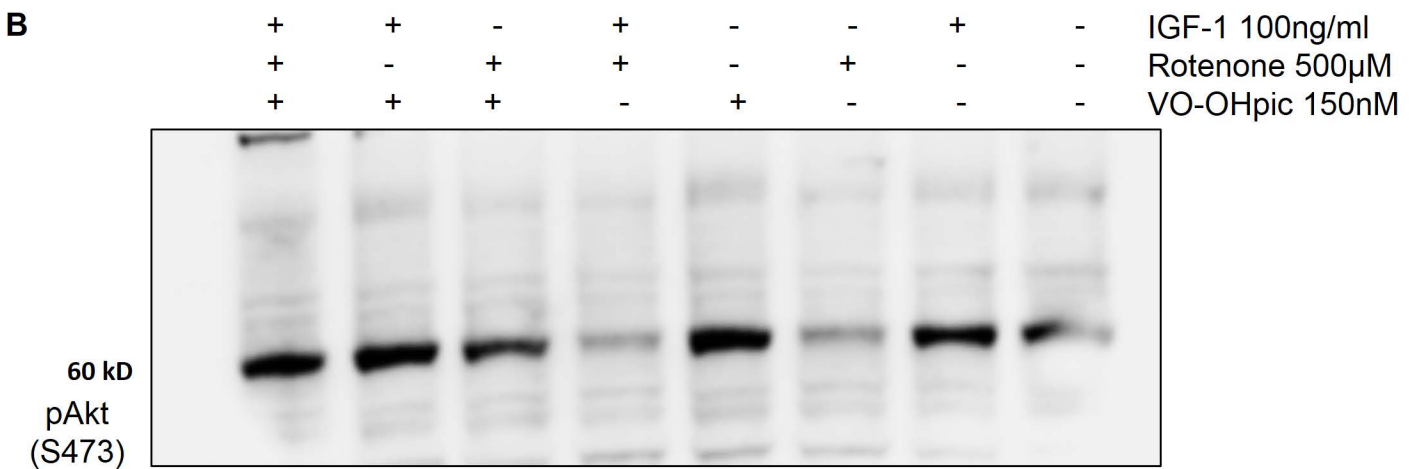

Supplement: Supplementary file 8 [file emmm0007-0059-sd8.pdf]

**Figure 8**

**D**

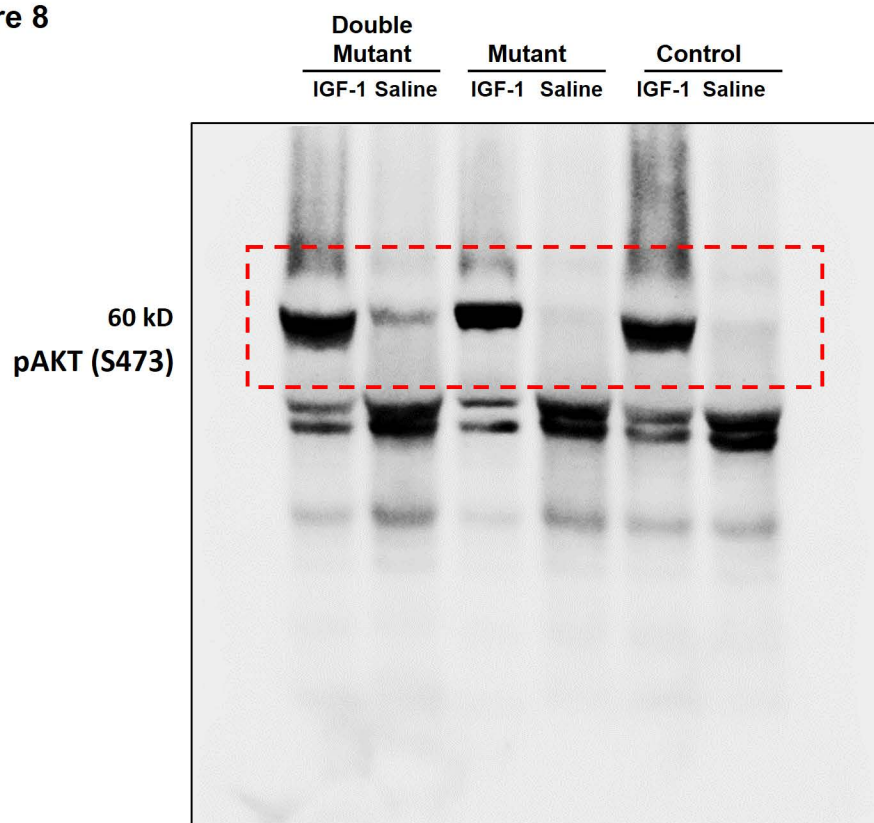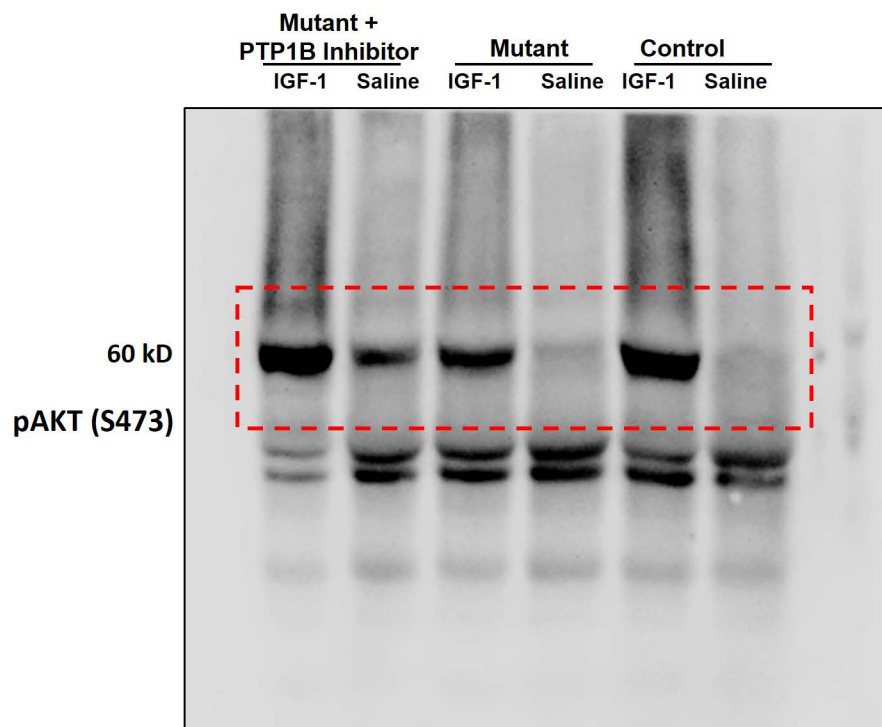

Supplement: Supplementary file 9 [file emmm0007-0059-sd9.pdf]
